# Physiological Roles of the Dual Phosphate Transporter Systems in Low and High Phosphate Conditions and in Capsule Maintenance of *Streptococcus pneumoniae* D39

**DOI:** 10.3389/fcimb.2016.00063

**Published:** 2016-06-20

**Authors:** Jiaqi J. Zheng, Dhriti Sinha, Kyle J. Wayne, Malcolm E. Winkler

**Affiliations:** Department of Biology, Indiana University BloomingtonBloomington, IN, USA

**Keywords:** PhoU, PnpRS two-component system (TCS), PnpR~P phosphorylation, Pst1 and Pst2 P_i_ ABC transporters, NptA Na^+^/P_i_ co-transporter

## Abstract

Unlike most bacteria, *Streptococcus pneumoniae* (pneumococcus) has two evolutionarily distinct ABC transporters (Pst1 and Pst2) for inorganic phosphate (P_i_) uptake. The genes encoding a two-component regulator (PnpRS) are located immediately upstream of the *pst1* operon. Both the *pst1* and *pst2* operons encode putative PhoU-family regulators (PhoU1 and PhoU2) at their ends. This study addresses why *S. pneumoniae* contains dual P_i_ uptake systems and the regulation and contribution of the Pst1 and Pst2 systems in conditions of high (mM) P_i_ amount and low (μM) P_i_ amount. We show that in unencapsulated mutants, both *pst1* and *pst2* can be deleted, and P_i_ is taken up by a third Na^+^/P_i_ co-transporter, designated as NptA. In contrast, either *pst1* or *pst2* is unexpectedly required for the growth of capsule producing strains. We used a combination of mutational analysis, transcript level determinations by qRT-PCR and RNA-Seq, assays for cellular PnpR~P amounts by SDS-PAGE, and pulse-P_i_ uptake experiments to study the regulation of P_i_ uptake. In high P_i_ medium, PhoU2 serves as the master negative regulator of Pst2 transporter function and PnpR~P levels (post-transcriptionally). Δ*phoU2* mutants have high PnpR~P levels and induction of the *pst1* operon, poor growth, and sensitivity to antibiotics, possibly due to high P_i_ accumulation. In low P_i_ medium, Pst2 is still active, but PnpR~P amount and *pst1* operon levels increase. Together, these results support a model in which pneumococcus maintains high P_i_ transport in high and low P_i_ conditions that is required for optimal capsule biosynthesis.

## Introduction

**Figure 1:**
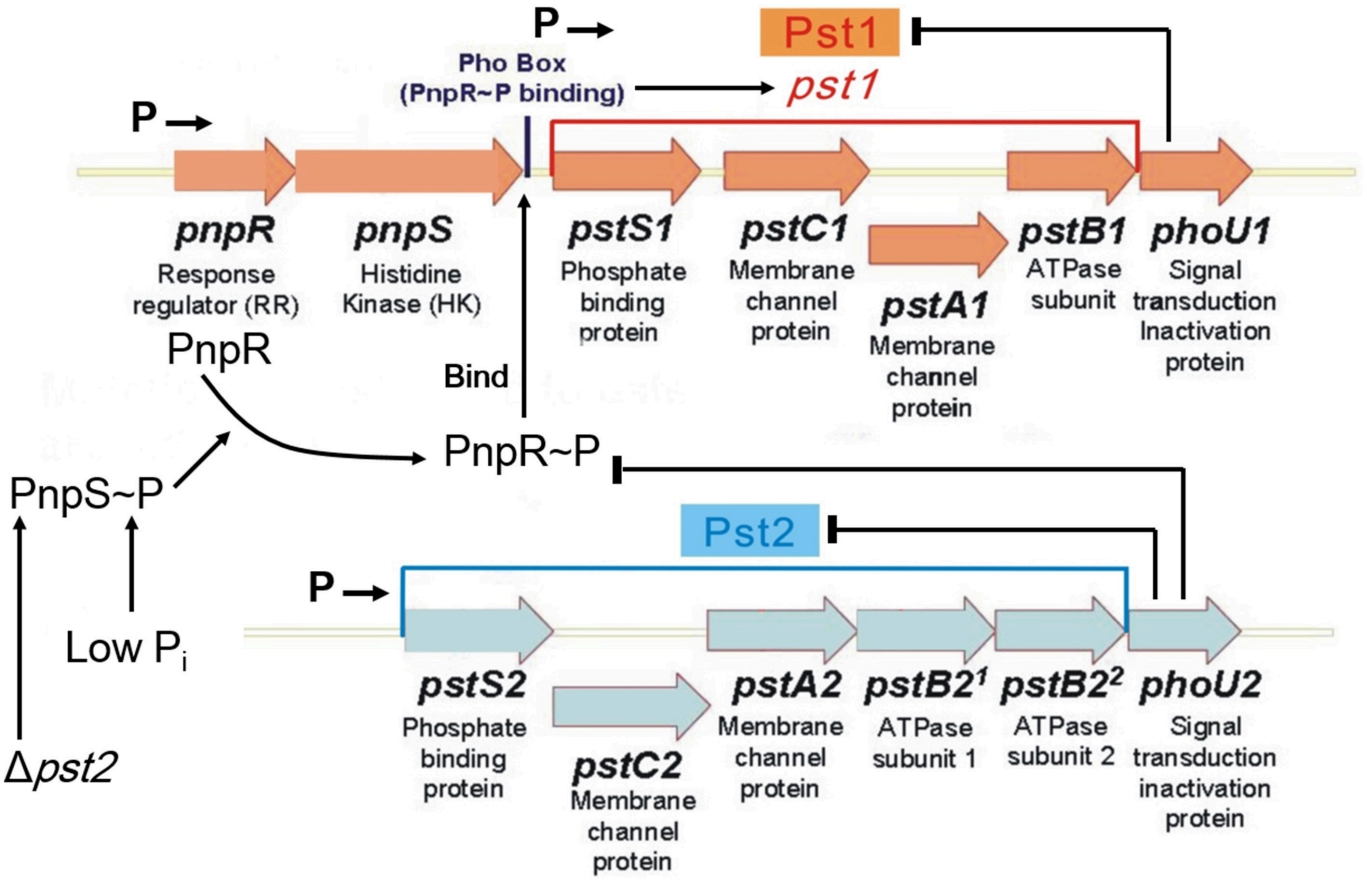
**Model for regulation of the dual Pst1 and Pst2 ABC P_i_ transporters in *Streptococcus pneumoniae* D39**. Genomic sequencing and transcriptome analyses show that *pnpRS, pst1-phoU1*, and *pst2-phoU2* are organized into three distinct operons that are transcribed from separate promoters, indicated by Ps followed by arrows (see text). The Pst2 ABC P_i_ transporter is constitutively expressed in both high and low P_i_ conditions. PhoU2 inhibits phosphorylation of the PnpR response regulator (RR) and negatively regulates the transport activity of Pst2. In the absence of PhoU2, PnpR is phosphorylated by the PnpS histidine kinase (HK). PnpR~P binds to the Pho box upstream of the *pst1-phoU1* operon and activates transcription and expression of the genes encoding the Pst1 transporter and PhoU1 regulator; however, PnpR~P does not autoregulate its own (*pnpRS*) transcription. In contrast to PhoU2, PhoU1 negatively regulates the transport activity of Pst1, but does not regulate phosphorylation of PnpR. In the absence of the Pst2 transporter or in low P_i_ conditions, the PnpRS two component system (TCS) activates the expression of the genes encoding the Pst1 transporter and PhoU1 regulator. Either the Pst1 or Pst2 P_i_ transporter is required in encapsulated cells. Therefore, the regulated Pst1 and constitutive Pst2 P_i_ transport systems may act as a redundant failsafe to ensure that capsule biosynthesis is maintained during variations in P_i_ conditions. See the text for additional details.

Phosphorus is an essential element in all cells because of its structural and metabolic roles in nearly all biological processes, including the composition of nucleic acids, phospholipids, and energy intermediates. A preferred source of phosphorous for bacterial cells is environmental inorganic orthophosphate (${\text{PO}}_{4}^{-}$; P_i_). The mechanism of extracellular P_i_ uptake has been studied intensively in *Escherichia coli* and *Bacillus subtilis* as model organisms (Hulett, 1993; Takemaru et al., 1996; Wanner, 1996; Qi et al., 1997; Lamarche et al., 2008; Hsieh and Wanner, 2010; Botella et al., 2011, 2014), and recently in other bacterial species (Braibant et al., 1996; Gonin et al., 2000; Zaborina et al., 2008; Rifat et al., 2009; Shi and Zhang, 2010; Burut-Archanai et al., 2011; Cheng et al., 2012; Wang et al., 2013; de Almeida et al., 2015; Lubin et al., 2016). Generally, bacterial high-affinity P_i_ uptake systems consist of an ATP-binding cassette (ABC) transporter, designated as Pst (for phosphate-specific transporter), which contains at least four component subunits: an extracellular P_i_ binding protein (PstS), two transmembrane channel proteins (PstCA), and a cytoplasmic ATPase (PstB) (see Figure 1; Hsieh and Wanner, 2010). The expression of most bacterial Pst transporters is regulated at the transcriptional level by a two-component regulatory system (TCS), which has different designations in different bacteria (Hulett, 1993; Novak et al., 1999; Throup et al., 2000; Howell et al., 2006; Glover et al., 2007). Many bacteria also regulate P_i_ uptake by an ancillary negative regulatory protein, designated PhoU (Steed and Wanner, 1993; Botella et al., 2011, 2014; de Almeida et al., 2015; Lubin et al., 2016).

In *E. coli* and related enterobacteria, the histidine kinase (HK) and response regulator (RR) that mediate P_i_ transport are designated as PhoR and PhoB, respectively, and the regulation of P_i_ uptake involves a PhoU regulator (Hsieh and Wanner, 2010; Gardner et al., 2014, 2015). Briefly, when [P_i_] > 4.0 μM, the expression of the *phoB-phoR* regulator and *pst* transporter operons is inhibited by PhoU by a mechanism described below (Hsieh and Wanner, 2010). When the [P_i_] is depleted to < 4.0 μM, PhoU releases inhibition of the PhoR HK and the PstB subunit of the transporter, allowing autophosphorylation of the PhoR HK, phosphoryl transfer to the PhoB RR, and activation of transcription by PhoB~P of operons in the phosphate (pho) regulon, including the *pst* transporter, the *phoB-phoR* regulator, and other operons involved in the uptake and assimilation of phosphorous-containing compounds (Wanner, 1996; Hsieh and Wanner, 2010). PhoB~P activates transcription by binding to the Pho box sequence upstream from the promoters of the regulon operons, including *phoB-phoR*, which provides autoregulation of the TCS proteins (Wanner, 1996; Martin, 2004; Lubin et al., 2016). Usually, the sequence of the consensus Pho box is different between Gram-negative (e.g., *E. coli*) and Gram-positive (e.g., *B. subtilis*) bacteria (Martin, 2004). Since the Pst transporter is not activated in *E. coli* at high P_i_ concentrations, this system is considered as a high-affinity transporter that works predominantly at low P_i_ concentrations (Wanner, 1996). PhoB/R, the Pst transporter, and members of the Pho regulon are important for virulence in many pathogenic Gram-negative bacteria, including *E. coli, Vibrio cholerae, Proteus mirabilis*, and *Pseudomonas aeruginosa* (Jacobsen et al., 2008; Lamarche et al., 2008; Zaborina et al., 2008; Pratt et al., 2010; Chekabab et al., 2014a,b). In *P. aeruginosa*, the PstS P_i_ binding protein also plays roles in adhesion and a P_i_-independent role in biofilm formation (Zaborina et al., 2008; Neznansky et al., 2014; Shah et al., 2014).

PhoU is a negative regulator of Pho regulon expression in *E. coli* and many other bacteria (Muda et al., 1992; Wanner, 1996; Hsieh and Wanner, 2010; Gardner et al., 2014; de Almeida et al., 2015). Although, PhoU is an important regulator in many bacteria, it is notably absent from certain Gram-positive bacteria, including *B. subtilis* (Qi et al., 1997; Moreno-Letelier et al., 2011). *phoU* deletion in *E. coli, P. aeruginosa*, and *Mycobacterium marinum* leads to growth defects (Steed and Wanner, 1993; Wanner, 1996; Rice et al., 2009; Wang et al., 2013; de Almeida et al., 2015). In *E. coli*, this growth defect is reversed by deletion of the *pst* transporter operon or the *phoBR* TCS operon (Steed and Wanner, 1993; Wanner, 1996). These observations suggest that the growth defect of *phoU* mutants is caused by unregulated function of the Pst transport system, leading to excess P_i_ accumulation (Wanner, 1996; Rice et al., 2009). Δ*phoU* mutants also accumulate increased amounts of poly-orthophosphate (poly-P_i_) in *E. coli, M. marinum, P. aeruginosa*, and *Caulobacter crescentus* (Morohoshi et al., 2002; Hirota et al., 2013; Wang et al., 2013; de Almeida et al., 2015; Lubin et al., 2016). Poly-P_i_ also accumulates in *E. coli* K-12 cells in stationary phase in high P_i_ medium, and this accumulation is correlated with inhibition of biofilm formation mediated by PhoB~P RR phosphorylation with acetyl-phosphate acting as donor (Grillo-Puertas et al., 2016). The rate of P_i_ uptake was reported to increase in an *E. coli* Δ*phoU* in one study (Rice et al., 2009), but not in another (Steed and Wanner, 1993). Besides defective growth, Δ*phoU* mutants exhibit higher sensitivity to a diverse range of antibiotics in *E. coli, Mycobacterium tuberculosis, M. marinum*, and *P. aeruginosa* (Li and Zhang, 2007; Shi and Zhang, 2010; Wang et al., 2013; de Almeida et al., 2015) and a defect in mutagenic DNA break repair in *E. coli* (Gibson et al., 2015).

Recent papers demonstrate that *E. coli* PhoU interacts with the PAS domain of the PhoR HK and with the PstB ATPase protein, in support of the dual inhibition of Pho regulon transcription and Pst mediated transport in *E. coli* (Gardner et al., 2014, 2015). Three crystal structures of PhoU-like proteins have been reported from *Aquifex aeolicus, Thermotoga maritima*, and *P. aeruginosa* (Liu et al., 2005; Oganesyan et al., 2005; Lee et al., 2014), showing that PhoU consists of two symmetric, three-alpha-helix bundles. However, these PhoU proteins showed several quaternary structures in crystals, including monomer, dimer, or hexamer packing (Liu et al., 2005; Oganesyan et al., 2005; Lee et al., 2014). Gel filtration shows that purified *E. coli* PhoU forms a dimer in solution (Gardner et al., 2014). In addition, divalent cation binding of magnesium and manganese is required for *E. coli* PhoU binding to the cytoplasmic side of the inner membrane and may play a role in formation of a ternary regulatory complex containing PhoU, PhoR, and PstB (Gardner et al., 2014). On the other hand, a recent study suggests that a different paradigm operates in *C. crescentus*, where PhoU does not modulate PhoR HK activity directly (Lubin et al., 2016). Instead, PhoU may negatively regulate the activity of the Pst transporter in response to P_i_ availability in *C. crescentus* (Lubin et al., 2016).

Besides Pst ABC transporters, P_i_ is taken up by symporter secondary transport systems. In *E. coli*, two additional P_i_ transporters, PitA and PitB, have been identified that are symporters of divalent cations, such as Mg^2+^ and Ca^2+^ (van Veen et al., 1994; Wanner, 1996; Harris et al., 2001; Jackson et al., 2008). PitA and PitB have been considered as low-affinity P_i_ transporters that predominantly function in high P_i_ environments (Wanner, 1996; Hsieh and Wanner, 2010). Moreover, expression of *pitA* is induced by Zn^2+^ addition (Jackson et al., 2008), suggesting that PitA may act primarily as a metal ion transporter instead of a P_i_ transporter (Beard et al., 2000).

Unlike *E. coli, B. subtilis*, or *C. crescentus*, which contain only one Pst transporter, *Streptococcus pneumoniae* (pneumococcus) encodes two evolutionarily distinct P_i_ ABC pump transporters, Pst1 and Pst2 (Figure 1; Lanie et al., 2007; Moreno-Letelier et al., 2011). The multigene *pst1* and *pst2* operons are located at different locations in the pneumococcus chromosome (Lanie et al., 2007). Only one *phoBR*-like TCS, *pnpRS*, encoding the PnpR RR and the PnpS HK, is encoded upstream of the *pst1* operon, and both the *pst1* and *pst2* transporter operons encode separate PhoU-family regulators, designated PhoU1 and PhoU2 (Figure 1). The PnpRS TCS, Pst1 transporter, and PhoU1 were initially studied in unencapsulated *S. pneumoniae* laboratory strain R6x (Novak et al., 1999), before the discovery of the second Pst2 transporter and PhoU2 regulator. This work indicated that *pnpRS* operon expression was not regulated by P_i_ amount, and that mutants deficient in the PstB1 ATPase subunit seemed to show decreased P_i_ uptake in certain growth media (Novak et al., 1999). Subsequent work suggests that upregulation of *pst1* operon expression is correlated with increased β-lactam antibiotic resistance in low-affinity *pbp2x* mutants and some clinical isolates of *S. pneumoniae* (Soualhine et al., 2005; Engel et al., 2014).

*S. pneumoniae* is a common commensal bacterium that primarily colonizes the human nasopharynx (Donkor, 2013; Chao et al., 2014; Hakansson et al., 2015; Short and Diavatopoulos, 2015), but can become an opportunistic pathogen, causing several serious respiratory and invasive diseases (Henriques-Normark and Tuomanen, 2013; Vernatter and Pirofski, 2013; Ferreira and Gordon, 2015; Gratz et al., 2015; Oliver and Swords, 2015). Therefore, the Pst1 and Pst2 transporters must mediate P_i_ acquisition from several niches with vastly different P_i_ concentrations in human hosts (Orihuela et al., 2001; Wilson, 2005, 2008). Signature-tagged mutagenesis (STM) screens and a study of the role of the PnpR RR (also called RR04) indicated that the *pnpRS, pst1*, and *pst2* operons are all required for full pneumococcal virulence (Polissi et al., 1998; Throup et al., 2000; Hava and Camilli, 2002; McCluskey et al., 2004; Paterson et al., 2006; Trihn et al., 2013). Consistent with these earlier studies, a recent Tn-Seq study showed that PhoU1 is important for nasopharynx colonization, whereas PhoU2 is important for lung infection (van Opijnen and Camilli, 2012).

In this report, we studied the transcriptional and functional regulation of the pneumococcal Pst1 and Pst2 P_i_ transporters under growth conditions containing high (≈18 mM) or low (≈100 μM) concentrations of P_i_. Our results show that *pst2* operon transcription is constitutive, but Pst2 transporter activity is negatively regulated by PhoU2. In addition, PhoU2 negatively regulates PnpR RR phosphorylation and transcription of the *pst1* operon at high concentrations of P_i_. Therefore, PhoU2 resembles *E. coli* PhoU in that it functions in regulating the level of RR phosphorylation, besides modulating Pst2 transporter activity. In contrast, PhoU1 resembles *C. crescentus* PhoU in that it only regulates Pst1 transporter activity and does not modulate PnpRS TCS function. Our results also indicate that encapsulated *S. pneumoniae* requires the function of Pst1 or Pst2 for growth, whereas a symporter, named NptA, can provide sufficient P_i_ to allow the growth of unencapsulated mutants in high P_i_ conditions.

## Materials and methods

### Bacterial strains and growth conditions

Strains used in this study are listed in Table S1. Encapsulated strains were derived from parent strain IU1781 (D39 *cps*^+^ *rpsL1*), and unencapsulated strains were derived from parent strains IU1945 (D39 Δ*cps*), IU1824 (D39 Δ*cps rpsL1*), and IU3309 (D39 Δ*cps2E rpsL1*), which are derivatives of serotype 2 *S. pneumoniae* strain D39 IU1690 (Lanie et al., 2007). Strains containing antibiotic markers were constructed by transformation of competent pneumococcal cells with linear DNA amplicons synthesized by overlapping fusion PCR (Ramos-Montanez et al., 2008; Tsui et al., 2010). Strains containing markerless alleles in native chromosomal loci were constructed using allele replacement via the Pc-[*kan-rpsL*^+^] (Janus cassette; Sung et al., 2001). Primers used to synthesize different amplicons are listed in Table S2. All constructs were confirmed by DNA sequencing of chromosomal regions corresponding to the amplicon region used for transformation. Bacteria were grown on plates containing trypticase soy agar II (modified; Becton-Dickinson) and 5% (vol/vol) defibrinated sheep blood (TSAII-BA). Plates were incubated at 37°C in an atmosphere of 5% CO_2_. For selections of transformants, TSAII-BA plates contained 250 μg/mL kanamycin, 0.3 μg/mL erythromycin, or 250 μg/mL streptomycin. Strains were cultured statically in Becton-Dickinson brain heart infusion (BHI) broth at 37°C in an atmosphere of 5% CO_2_, and growth was monitored by OD_620_ as described before (Tsui et al., 2016). Transformants were single-colony-isolated on TSAII-BA plates containing antibiotics twice before growth in antibiotic-containing BHI broth for storage (Tsui et al., 2016). All mutant constructs were confirmed by DNA sequencing of chromosomal regions corresponding to the amplicon region used for transformation.

Static growth in BHI broth, which contains ≈18 mM P_i_, was used as a high P_i_ condition. For growth curves, strains were inoculated into 3 mL of BHI broth, serially diluted, and grown overnight. The next day, cultures with OD_620_ = 0.1–0.3 were diluted into 5 mL of fresh BHI broth to OD_620_ ≈ 0.002, and growth was monitored hourly. C+Y medium (Lacks and Hotchkiss, 1960) was used for studies of moderately low P_i_ condition. We determined that C+Y broth (no added P_i_) already contains ≈1.5 mM P_i_ (see Results). A modified chemically defined medium (mCDM) (Carvalho et al., 2013) was used for moderate and low P_i_ conditions. To optimize growth, the concentrations of choline-HCl and all amino acids amounts were increased by 1000-fold and tyrosine was added to 100 mg/L compared to the CDM recipe in Carvalho et al. (2013). In addition, 40 mM MOPS buffer was added to mCDM, which was adjusted to a final pH = 7.4 with 10 M NaOH. mCDM contains 36.4 mM P_i_(Carvalho et al., 2013). mCDM with no P_i_ was made by omitting KH_2_PO_4_ and K_2_HPO_4_ and adding KCl to 50.8 mM. mCDM media with 2 mM or 1 mM P_i_ was made by mixing mCDM and mCDM lacking P_i_ in a ratio of 2–34.4 or 1–35.4, respectively. mCDM with 1 mM P_i_ was diluted 10 or 100-fold with mCDM lacking P_i_ make mCDM with 100 μM and 10 μM P_i_, respectively. For growth in mCDM, 3 mL overnight cultures were grown as described above, and the next day, cultures with OD_620_ = 0.1–0.3 were centrifuged (5125 × g, 5 min, 25°C), washed with 3 mL mCDM lacking P_i_ twice, and resuspended in 3 mL mCDM lacking P_i_. Cells were then diluted in 5 mL of mCDM with 2, 1 mM, 100, 10 μM, or no P_i_ to OD≈0.005 and growth of static cultures in an atmosphere of 5% CO_2_ was monitored hourly at OD_620._

### Antibiotic disk diffusion assays

Overnight cultures were diluted and grown in 5 mL of BHI to OD_620_ ≈ 0.1. 100 μL of cultures were mixed with 3 mL of nutrient-broth soft agar [0.8% (w/v) nutrient broth and 0.7% (w/v) Bacto Agar (Difco)] and poured onto TSAII-BA plates. After 15 min, antibiotics disks were placed at the middle of plates, which were incubated at 37°C in an atmosphere of 5% CO_2_ overnight for 16 h. Diameters of zones of growth inhibition were measured with a ruler, and *P*-values were calculated by unpaired *t*-test in GraphPad Prism. Antibiotic disks were from Becton, Dickinson Co.: cefotaxime (30 μg); cefazolin (30 μg); cefamandole (30 μg); ceftazidime (30 μg); amdinocillin (10 μg); vancomycin (30 μg); gentamicin (120 μg); and tetracycline (30 μg).

### RNA preparation, qRT-PCR, and RNA-seq analyses

To study high P_i_ conditions, overnight cultures were diluted and grown in 5 mL of BHI to OD ≈0.15. Cells were collected by centrifuging at 16,000 × g for 5 min at 4°C. 1 mL of RNApro solution (MP Biomedicals) was added to resuspend cell pellets. The suspension was transferred to a Lysing Matrix B tube (MP Biomedicals), which was shaken 3X in FastPrep homogenizer (6.0 M/s for 40 s each). Cell debris and lysing matrix were removed by centrifugation at 16,000 × *g* for 5 min at 4°C. 700 μL of supernates was transferred to a new microcentrifuge tube and incubated at room temperature for 5 min. 300 μL of chloroform was then added followed by incubation at room temperature for 5 min. Mixtures were centrifuged at 16,000 × *g* for 5 min at 4°C. 280 μL of the upper, aqueous phase was collected and mixed with 140 μL of 100% Ethanol in a new microcentrifuge tube for RNA precipitation. RNA purification was done using miRNeasy minikit (Qiagen), including on-column treatment with DNase I (Qiagen), following the manufacturer's instructions. 5 μg of purified RNA was treated by DNase from a DNA-free DNA removal kit (Ambion). 125 ng of treated RNA was used to synthesize cDNA by a qScript Felex cDNA synthesis kit (Quanta Biosciences). Synthesized cDNA was diluted 1:6 in water and then serially diluted 1:5 in water three more times. qRT-PCR reactions contained 10 μL of 2 × Brilliant III Ultra-Fast SYBR Green QPCR Master Mix (Agilent), 2 μL of each 2 μM primers (Table S3), 0.3 μL of a 1:500 dilution of ROX reference dye, and 6 μL of diluted cDNA. Samples were run in an MX3000P thermocycler (Stratagene) with Program MxPro v. 3.0. Transcript amounts were normalized to *gyrA* mRNA amount and compared with transcript amounts of the wild-type parent strain by unpaired *t*-test in GraphPad Prism (Kazmierczak et al., 2009).

To study low P_i_ conditions, bacteria from overnight BHI broth cultures were washed, diluted, and grown in 5 mL of mCDM medium containing 36.4 mM P_i_ to OD_620_ ≈ 0.15. Cells were collected by centrifugation, washed twice with 5 mL mCDM lacking P_i_, and resuspended in mCDM containing 36.4 mM P_i_ or 10 μM P_i_. Cultures were incubated at 37°C in an atmosphere of 5% CO_2_ for 30 min. Lysis, RNA extraction, purification, and qRT-PCR reactions were performed as described above, except that 16S rRNA was used to normalize transcript amounts, because *gyrA* was down-regulated under low P_i_ condition. All transcript amounts were compared with the wild-type parent strain grown in mCDM containing 36.4 mM.

RNA samples for RNA-Seq analyses were prepared from 30 mL cultures as described previously (Hoover et al., 2015) cDNA library construction, single-end, 100 bp-sequencing on a HiSeq 2000 sequencer (Illumina), and bioinformatic analyses were performed as described in Hoover et al. (2015). False-discovery rates (FDR) were calculated using Benjamini and Hochberg's algorithm (Benjamini and Hochberg, 1995) and a gene or region was defined as differentially expressed if it had an up- or down-fold change of 1.8 with a FDR < 0.05. RNA-Seq data were deposited in the NCBI GEO database under accession number GSE80637.

### Phos-tag SDS-PAGE and western blot

Phos-tag SDS-PAGE and standard Western blotting were carried out as described previously (Wayne et al., 2010, 2012; Tsui et al., 2014). To study high P_i_ conditions, overnight BHI broth cultures were diluted and grown up to OD_620_ ≈ 0.2 in 30 mL of BHI. Cells expressing PnpR-L-FLAG^3^ were lysed using a FastPrep homogenizer, and cell lysates were resolved by Phos-tag SDS-PAGE at 4°C (Wayne et al., 2012). Cells expressing PstS2-HA were lysed by the same method, but resolved by standard SDS-PAGE (Tsui et al., 2014). PnpR-L-FLAG^3^ and PstS2-HA were detected by Western blotting as described previously (Tsui et al., 2014) using anti-FLAG or anti-HA antibody as primary antibody. Chemiluminescent signal in protein bands was quantitated by using an IVIS imaging system as described in Wayne et al. (2010).

To study low P_i_ conditions, strains were grown in 30 mL of mCDM to OD_620_ ≈ 0.2 as described above. Cells were collected by centrifugation and washed twice with 30 mL of mCDM lacking P_i_. Cell pellets were resuspended in 30 mL of mCDM containing 36.4 mM P_i_ or lacking P_i_ and incubated statically at 37°C in an atmosphere of 5% CO_2_ for 40 min. Proteins samples were extracted and Phos-tag SDS-PAGE was performed as described above.

### Qualitative quellung assay for capsule

Overnight cultures were diluted and grown in 5 mL of BHI to OD≈0.15. 1 μL of culture was mixed with 1 μL of Type 2 pneumococcal antiserum (Statens Serum Institut) on a glass slide. A cover slip was placed on top of the mixture, which was viewed immediately with a 100X objective by a phase-contrast microscope. Cells surrounded by capsule appear enlarged or swollen.

### P_i_ concentration determination

The P_i_ amount in C+Y broth was determined by the colorimetric method described in Katewa and Katyare (2003). Briefly, standards were prepared by dilution of a KH_2_PO_4_ stock to give final P_i_ concentrations of 2.5, 5.0, 10.0, 20.0, and 40.0 μM P_i_ in 2.4 mL of water in glass tubes. 0.8 mL of 3N H_2_SO_4_ was added to each standard tube. 0.4 mL of 2.5% (w/v) ammonium molybdate (prepared in 3N H_2_SO_4_) was added in each tube. Last, 0.4 mL of reducing agents (20 mg of hydrazine sulfate and 20 mg of ascorbic acid dissolved in 1 mL of 0.1N H_2_SO_4_) was added to each tube. After 2 h at room temperature, A_820_ was determined and plotted to generate a standard curve. C+Y broth (no added P_i_) was diluted 100X with water, P_i_ content was assayed as described above, and P_i_ concentration was determined from the standard curve.

### P_i_ uptake assays

To study high P_i_ conditions, strains were grown in 5 mL of BHI broth to OD_600_ ≈ 0.2. Cells were centrifuged at 5125 × *g* for 5 min at room temperature, washed twice with 5 mL of mCDM lacking P_i_, and resuspended at room temperature in 5 mL of mCDM lacking P_i_. K_2_H^32^PO_4_ (10^7^–10^8^ dpm; 8500–9120 Ci/mmole; Perkin Elmer) was added to a final concentration of 1 mM at *t*=0, and 100 μL of cells was collected by vacuum filtration (0.22 μm GSWP; 13 mm diameter; Millipore) at 1, 2, 4, 6, 10 min after addition of ^32^P. Filters were washed 3X with 3 mL of room-temperature 1 × PBS (Ambion). Washed filters were transferred individually into 20-mL glass scintillation vials to which 5 mL of a biodegradable counting cocktail was added. Dpm of each sample was determined using a TRI-CARB 2100TR Liquid Scintillation Counter (Perkin Elmer), P_i_ amount incorporated at each time point was calculated.

To study low P_i_ condition, strains were grown to OD_600_ ≈ 0.2 in mCDM containing 36.4 mM Pi as described above. Cells were centrifuged at 5125 × g for 5 min at room temperature, and washed twice with 5 mL of mCDM lacking P_i_. Cell pellets were resuspended in 5 mL of mCDM lacking P_i_ and incubated 1 h at 37°C in an atmosphere of 5% CO_2_. 200 μM of K_2_H^32^PO_4_ (10^7^–10^8^ dpm) was added at *t* = 0, and samples were withdrawn, filtered, and counted as described above.

## Results

### Δ*phoU2* mutants show a growth defect and increased sensitivity to a range of antibiotics that is reversed by inactivation of Pst2 transport

**Figure 2:**
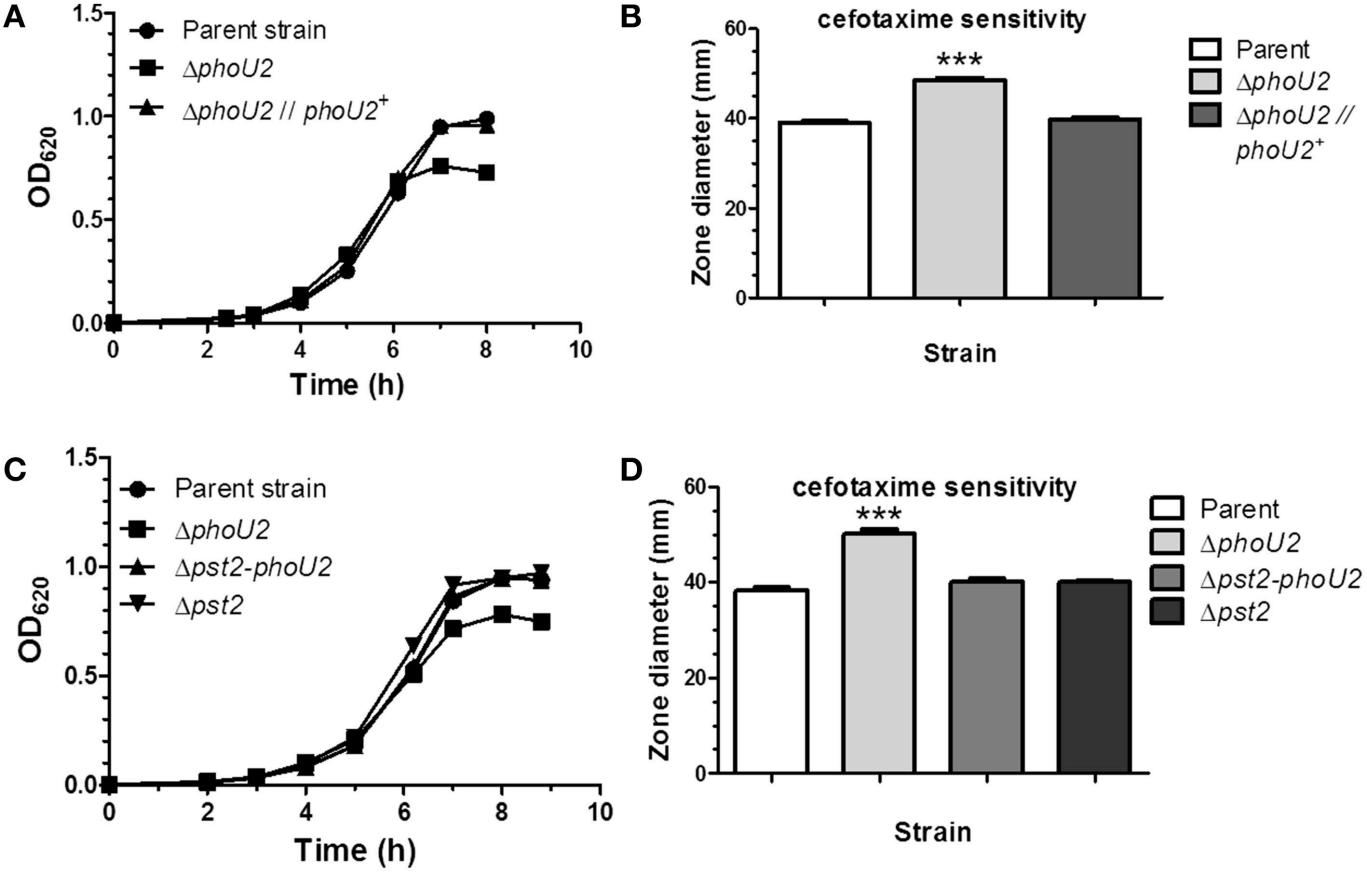
**Deletion of *phoU2* leads to a lower growth yield and increased β-lactam antibiotic sensitivity that are reversed by a Δ*pst2* mutation. (A)** Representative growth curves in BHI broth (≈18 mM P_i_) of encapsulated parent strain (IU1781), a Δ*phoU2* mutant (IU6375), and a Δ*phoU2* mutant complemented by ectopic expression of PhoU2^+^ (IU6397). Strains were grown as described in Materials and Methods. A linear scale for OD_620_ is used to emphasize differences in growth yields. Growth yields and rates are quantitated for multiple determinations in Table S4. **(B)** Cefotaxime sensitivity assays of encapsulated parent strain (IU1781), a Δ*phoU2* mutant (IU6375), and a PhoU2^+^-complemented Δ*phoU2* mutant (IU6397). Cefotaxime disk sensitivity assays of bacteria grown in BHI broth were performed as described in Material and Methods. *P*-values were calculated by unpaired *t*-tests relative to the parent strain using GraphPad Prism; (*n* ≥ 3); ^***^P < 0.001. Increased sensitivity to other β-lactam antibiotics, vancomycin, gentamicin, and tetracycline of a Δ*phoU2* mutant compared to its isogenic parent strain is shown in Table S5. **(C)** Representative growth curves of encapsulated parent strain (IU1781) and Δ*phoU2* (IU6375), Δ*pst2-phoU2* (IU6550), and Δ*pst2* (IU6610) mutants in BHI broth. **(D)** Cefotaxime sensitivity assays for encapsulated parent strain (IU1781), and Δ*phoU2* (IU6375), Δ*pst2-phoU2* (IU6550), and Δ*pst2* (IU6610) mutants. (*n* ≥ 3); ^***^P < 0.001.

The Pst transporter is not needed for growth of *E. coli* at high P_i_ concentrations >4 μM, and Δ*phoU* mutations lead to a severe growth defect that is reversed by inactivation of the Pst transporter (Steed and Wanner, 1993; Rice et al., 2009). To determine the roles of the Pst1 and Pst2 transporters and their regulation, we constructed a series of markerless deletion mutants [Δ*phoU2* (IU6375); Δ*phoU1* (IU6377); Δ*pnpRS* (IU6381); Δ*pst2* (IU6610); Δ*pst1* (IU6638)] in *S. pneumoniae* serotype 2 strain D39, which is encapsulated and virulent (Lanie et al., 2007; Figure 1, Table S1). Mutants were first grown in BHI broth, which contains a high P_i_ concentration (≈18 mM). Only the Δ*phoU2* mutant showed a significant decrease in growth yield compared to the parent and other mutants (Figure 2A, Figure S1A, Table S4). Growth yield was restored when the Δ*phoU2* mutation was complemented by an ectopic copy of the *phoU2*^+^ gene expressed from the P_*ftsA*_ promoter at the *bgaA* site (IU6397) (Figure 2A, Table S4). Similar results were obtained in mutants in unencapsulated D39 derivative strain K579 and E579 (data not shown).

Inactivation of *phoU* in *E. coli* generates higher sensitivity to various kinds of antibiotics than the parent strain (Li and Zhang, 2007). Since the pneumococcal Δ*phoU2* mutant shows a similar growth defect as the Δ*phoU* mutant in *E. coli* (Figure 2A, Figure S1A), we tested antibiotic sensitivity to several antibiotics. Of the Pho regulon mutants tested in the encapsulated strain, only the Δ*phoU2* mutant showed increased sensitivity to β-lactams and other classes of antibiotics, including glycopeptides and protein synthesis inhibitors, on plates that contain relatively high P_i_ content (Figure 2B, Figure S1B, and Table S5).

Previous work in *E. coli* (Steed and Wanner, 1993; Wanner, 1996; Rice et al., 2009; Hsieh and Wanner, 2010) and *C. crescentus*, in which PhoU is essential for growth (Lubin et al., 2016), indicated that the reduced growth of *phoU* deletion or depletion mutants could be reversed by inactivation of the Pst pump. This reversal was interpreted to mean that PhoU negatively regulates the Pst transporter itself, and in its absence, deleterious phosphate compounds accumulate that disrupt growth and metabolism. In the pneumococcal encapsulated D39 genetic background, a Δ*pst2-phoU2* deletion mutant lacking the Pst2 transporter and PhoU2 regulator grew similar to the parent strain and did not show increased sensitivity to antibiotics caused by the absence of PhoU2 alone (Figures 2C,D, Table S4). Likewise, single deletions of genes encoding each component of the Pst2 transporter restored growth yield of a Δ*phoU2* mutant back to wild-type (data not shown). Western blot analysis showed that the Δ*phoU2* mutation did not change the cellular amount of the PstS2 transporter subunit fused to the HA epitope tag (Figure S3), and by inference the amount of the Pst2 transporter. We conclude that the decreased growth yield and antibiotic sensitivity of encapsulated pneumococcus Δ*phoU2* mutants in high P_i_ conditions are dependent on function of the Pst2 transporter, consistent with negative regulation of the Pst2 transporter by PhoU2.

### PhoU2 negatively regulates transcription activation of the *pst1* operon by the PnpRS TCS in high P_i_ conditions

As noted in the Introduction, PhoU negatively regulates the PhoBR TCS in *E. coli*, but not in *C. crescentus* (Hsieh and Wanner, 2010; Lubin et al., 2016). Consequently, we tested whether Δ*phoU1* or Δ*phoU2* deletions affected transcript levels of the *pst1* or *pst2* operons under high P_i_ conditions (see Figure 1). We first performed RNA-Seq analyses of Δ*phoU2::kanrpsL*^+^ and Δ*phoU2*::*kanrpsL*^+^ Δ*phoU1*::P_c_-*erm* mutants growing in early-middle exponential phase in BHI broth, which contains a high (18 mM) concentration of P_i_ (Table S6). In both strains, only transcript amounts of the *pst1* transporter operon, including *phoU1* in the single mutant, were strongly induced (≈22X). The transcript amounts of the *pnpRS* regulator and *pst2* transporter operons were not induced, and the number of other genes in the Pho regulon appears to be limited in *S. pneumoniae* D39 (Table S6). Notably, pneumococcus encodes neither an alkaline phosphatase (*phoA*) nor a pathway for synthesis of teichuronic acids lacking phosphate (Wanner, 1996; Botella et al., 2011, 2014). Besides the strong induction of *pst1* operon transcription, there were small (2-4X) changes in the relative amounts of only a handful of other transcripts, including some corresponding to metabolic and stress-responsive genes, possibly reflecting the defective growth of these *phoU2* mutants. In both mutants, one of the stronger responses was a decrease in the relative transcript amounts of the genes encoding the glycerol facilitator (GlpF) and glycerol kinase (GlpK). A putative, somewhat degenerate Pho box is located -125 bp upstream of the *glpK* reading frame. Together, these results suggested that PhoU2 negatively regulates *pst1* operon expression under high P_i_ conditions, whereas the *pnpRS* and *pst2* operons are constitutively expressed.

These conclusions were confirmed by qRT-PCR analysis of combinations of markerless deletion mutations in the *pst* and *pnpRS* genes (Table 1). RNA-Seq transcriptome analysis indicates that the *pnpRS, pst1-phoU1*, and *pst2-phoU2* operons are separately transcribed (Table S6, Figure 1, Figures S2A,B). Hence, we quantitated the relative amounts of the *pnpR, pstS1*, and *pstS2* transcripts normalized to *gyrA* transcript amount by qRT-PCR to represent *pnpRS, pst1-phoU1*, and *pst2-phoU2* operon expression (see Materials and Methods; Wayne et al., 2012). Consistent with the RNA-Seq results, the Δ*phoU2* mutations caused ≈16X increase in *pst1-phoU1* operon transcript amount, but no change in expression of *pst2* or *pnpRS* operon (Table 1, line 2). In contrast, a Δ*phoU1* mutation did not cause a significant change in the relative amounts of transcript from any of the three operons (Table 1, line 3). No increase in *pst1-phoU1* transcript amount in the Δ*phoU2* mutant was detected in complementation experiments in which a wild-type copy of *phoU2*^+^ was expressed from an ectopic site (Table 1, line 4).

**Table 1:** **Relative transcript amounts of the *pst1, pst2*, and *pnpRS* operons in regulatory mutants^a^**.

**Strains^b^**	**Relative transcript amount of *pstS1*^c^**	**Relative transcript amount of *pstS2*^d^**	**Relative transcript amount of *pnpR*^e^**
1. Parent strain (IU1781)	≡1	≡1	≡1
2. Δ*phoU2* (IU6375)	+16.0 ± 1.8 (*n* = 4) (^***^)^f^	+1.2 ± 0.6 (*n* = 2) (ns)	−1.2 (*n* = 1)
3.Δ*phoU1* (IU6377)	+1.6 ± 0.4 (*n* = 4) (ns)	+1.3 ± 0.4 (*n* = 2) (ns)	+1.1 (*n* = 1)
4. Δ*phoU2*//*phoU2^+^* (IU6397)	+1.3 ± 0.3 (*n* = 3) (ns)	ND^g^	ND
4. Δ*pnpR* (IU6379)	−4.8±0.2 (*n* = 2) (^**^)	−1.1±0.1 (*n* = 2) (ns)	ND
6. Δ*pnpS* (IU6496)	−2.4±0.4 (*n* = 2) (^**^)	−1.4±0.1 (*n* = 2) (ns)	ND
7. Δ*pnpRS* (IU6381)	−2.0 ± 0.6 (*n* = 2) (^*^)	−1.1±0.2 (*n* = 2) (ns)	ND
8. Δ*phoU2* Δ*pnpR* (IU6573)	−4.4±1.1 (*n* = 4) (^***^)	ND	ND
9. Δ*phoU2* Δ*pnpS* (IU6595)	−2.1±0.3 (*n* = 4) (^**^)	ND	ND
10. Δ*phoU2* Δ*pnpRS* (IU6575)	−2.5±0.6 (*n* = 5) (^**^)	ND	ND
11. Δ*phoU2* Δ*phoU1* (IU6499)	+19.6 ± 1.6 (*n* = 5) (^***^)	−1.4±0.1(*n* = 2) (ns)	+1.0 (*n* = 1)
12. Δ*pst2-phoU2* (IU6550)	+43.8 ± 4.2 (*n* = 4) (^***^)	−	+1.1 ± 0.0 (*n* = 3) (ns)
13. Δ*pst2* (IU6610)	+44.8 ± 1.5 (*n* = 4) (^***^)	−	ND
14. Δ*pst2-phoU2* Δ*phoU1* (IU6612)	+44.8 ± 5.5 (*n* = 3) (^**^)	−	ND
15. Δ*pst1* (IU6638)	−	+1.0 (*n* = 1)	ND

a *RNA preparation and qRT-PCR were performed as described in Materials and Methods*.

b *Strains were markerless deletion mutants derived from encapsulated parent strain IU1781*.

c *Relative pstS1 gene transcript amount was used to represent pst1 operon expression*.

d *Relative pstS2 gene transcript amount was used to represent pst2 operon expression*.

e *Relative pnpR gene transcript amount was used to represent pnpRS operon expression*.

f ^***^P < 0.001; ^**^P < 0.01; ^*^P < 0.05; ns, not significant. P-values were calculated by an unpaired t-test in GraphPad Prism. P-value is not available when n = 1.

g *ND, not determined*.

**Figure 3:**
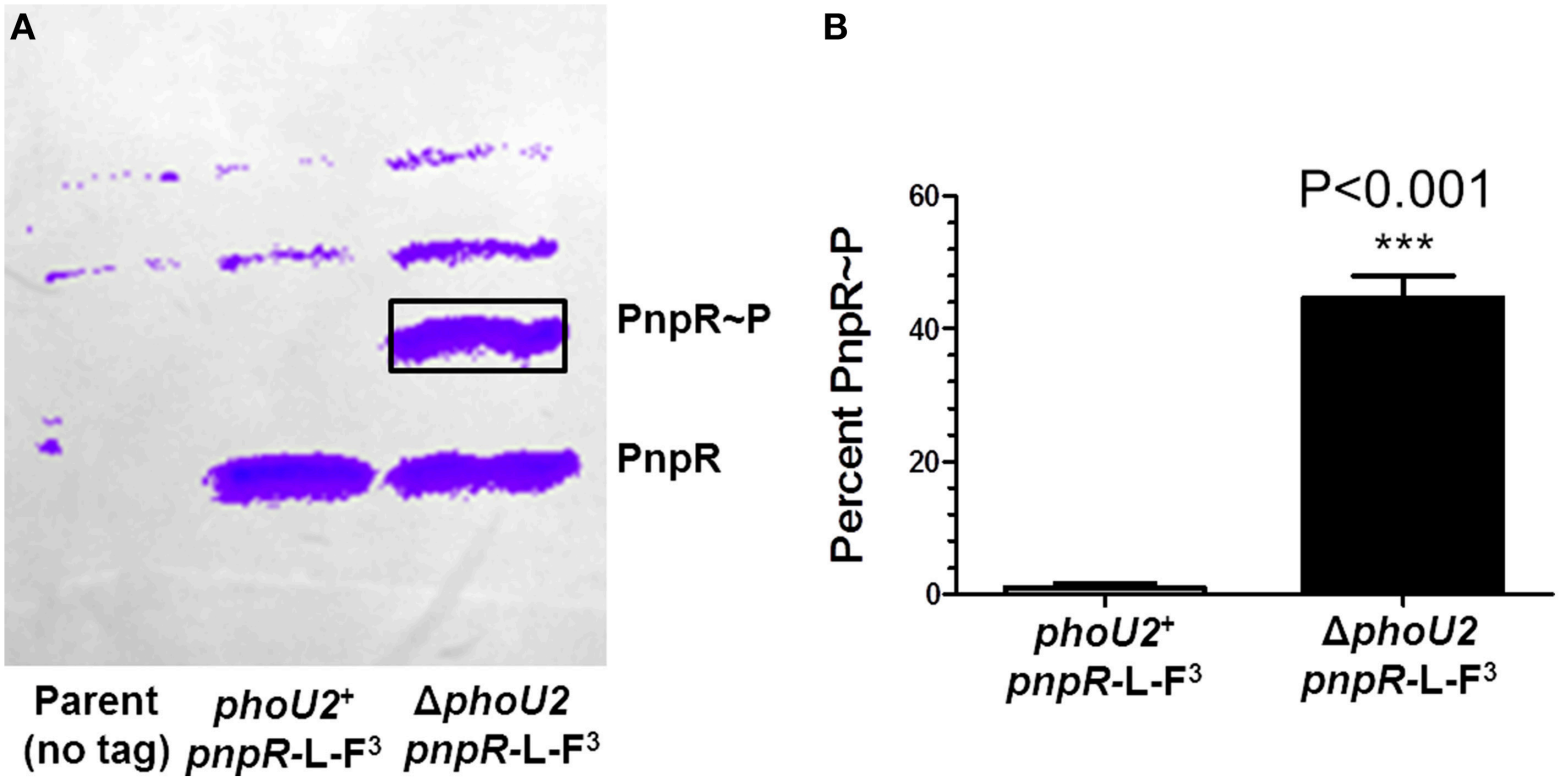
**Approximately 45% of PnpR is phosphorylated (PnpR~P) in a Δ*phoU2 mutant*. (A)** Representative Phos-tag SDS-PAGE of the encapsulated parent strain not expressing a FLAG-tagged protein (IU1781, lane 1) and of the *pnpR*-L-FLAG^3^ (IU6689, lane 2), and Δ*phoU2 pnpR*-L-FLAG^3^ (IU6687, lane 3) mutants growing exponentially in BHI broth. The gel was Western blotted with anti-FLAG antibody as described in Materials and Methods. The top two bands in all lanes are nonspecific. The upper anti-FLAG-specific band corresponds to phosphorylated PnpR~P, and the lower band corresponds to unphosphorylated PnpR. Control experiments show that the upper band is heat-sensitive, as expected for PnpR~P (Figure S4). **(B)** Quantification of 3 independent Phos-tag SDS-PAGE experiments. Less than 1% of PnpR was phosphorylated in the *phoU2*^+^ strain, whereas ≈45% of PnpR is phosphorylated (PnpR~P) in the Δ*phoU2* mutant. *P*-value was determined by an unpaired *t*-test in GraphPad Prism; ^***^P < 0.001.

We next determined that the increased transcription of the *pst1-phoU1* operon in the Δ*phoU2* mutant is mediated by the PnpRS TCS through increased phosphorylation of the PnpR~P RR. Δ*pnpR*, Δ*pnpS*, or Δ*pnpRS* mutants showed slightly reduced *pst1-phoU1* operon transcript amounts compared to the parent strain, with no change in *pst2-phoU2* operon expression (Table 1, lines 5–7). Likewise, Δ*pnpR* Δ*phoU2*, Δ*pnpS* Δ*phoU2*, and Δ*pnpRS* Δ*phoU2* double mutants showed reduced *pst1-phoU1* operon relative transcript amounts (Table 1, lines 8–10), instead of the sizable increase observed for the Δ*phoU2* single mutant containing an active PnpRS TCS (Table 1, line 2). To confirm directly that PhoU2 acts as a negative regulator of PnpRS function under high P_i_ conditions, we performed Phos-tag SDS PAGE analysis to determine PnpR~P RR phosphorylation levels (see Materials and Methods; Figure 3). In these experiments, we fused three tandem copies of the FLAG epitope tag to the C-terminus of the PnpR RR regulator expressed from its normal chromosomal locus (Figure 3, Table S1). The PnpR-L-FLAG^3^ RR induced *pst1-phoU1* transcription to a similar extent as wild-type (untagged) PnpR^+^ in a Δ*phoU2* mutant (data not shown). In the *phoU2*^+^ strain, essentially no PnpR~P (< 1%) was detected in cells growing exponentially in high-P_i_ BHI broth (Figure 3). In contrast, the Δ*phoU2* mutant contained ≈45% PnpR~P, which accounts for the high (≈16X) increase in *pst1-phoU1* transcript detected (Table 1, line 2). We conclude that PhoU2 negatively regulates PnpR~P amounts and transcription of the *pst1-phoU1* operon, but does not regulate transcription of the *pnpRS* or *pst2-phoU2* operon, which are constitutively expressed.

**Figure 4:**
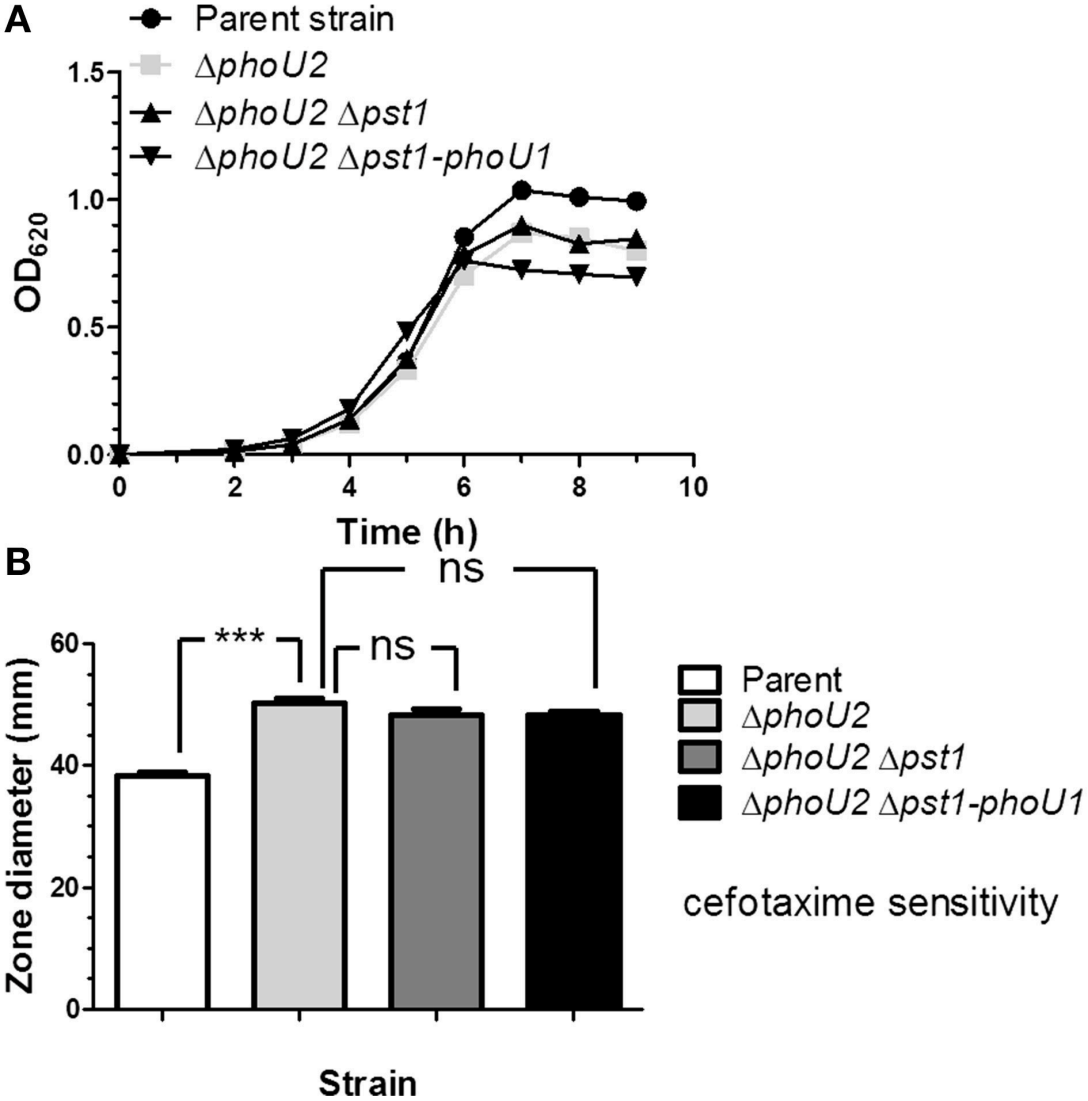
**Deletion of *pst1* or *pst1-phoU1* in a Δ*phoU2* mutant does not recover growth yield or antibiotic sensitivity. (A)** Representative growth curves of encapsulated parent strain (IU1781), and Δ*phoU2* (IU6375), Δ*phoU2* Δ*pst1* (IU6664), and Δ*phoU2* Δ*pst1-phoU1* (IU6854) mutants in BHI broth. Growth yields and rates are quantitated in Table S4. **(B)** Cefotaxime sensitivity assays for encapsulated parent strain (IU1781), and Δ*phoU2* (IU6375), Δ*phoU2* Δ*pst1* (IU6664), and Δ*phoU2* Δ*pst1-phoU1* (IU6854) mutants. (*n* ≥ 3); ^***^P < 0.001. See Materials and Methods, the legend to Figure 2, and the text for additional details.

Consistent with this interpretation, a putative PnpR~P binding site (Pho-box) sequence (TTTACACAATCTTTACA; Martin, 2004) is located 92 bp upstream of *pstS1* reading frame gene (Figure 1), but no recognizable Pho-box sequences can be found upstream of the *pnpRS* or *pst2-phoU2* operon. Finally, we tested whether induction of *pst1-phoU1* operon expression contributes to the growth defect and antibiotic sensitivity of a Δ*phoU2* mutant (Figure 2). A Δ*phoU2* Δ*pst1* or Δ*phoU2* Δ*pst1-phoU1* double mutant showed the same decrease in growth yield (Figure 4A, Table S4) and antibiotic sensitivity (Figure 4B) as the Δ*phoU2* single mutant, indicating that these defects were caused primarily by misregulation of the Pst2 transporter in high P_i_ conditions, instead of induced expression of the Pst1 transporter.

### Transcription of the *pst1* operon is further induced by the absence of the Pst2 transporter under high P_i_ conditions

**Figure 5:**
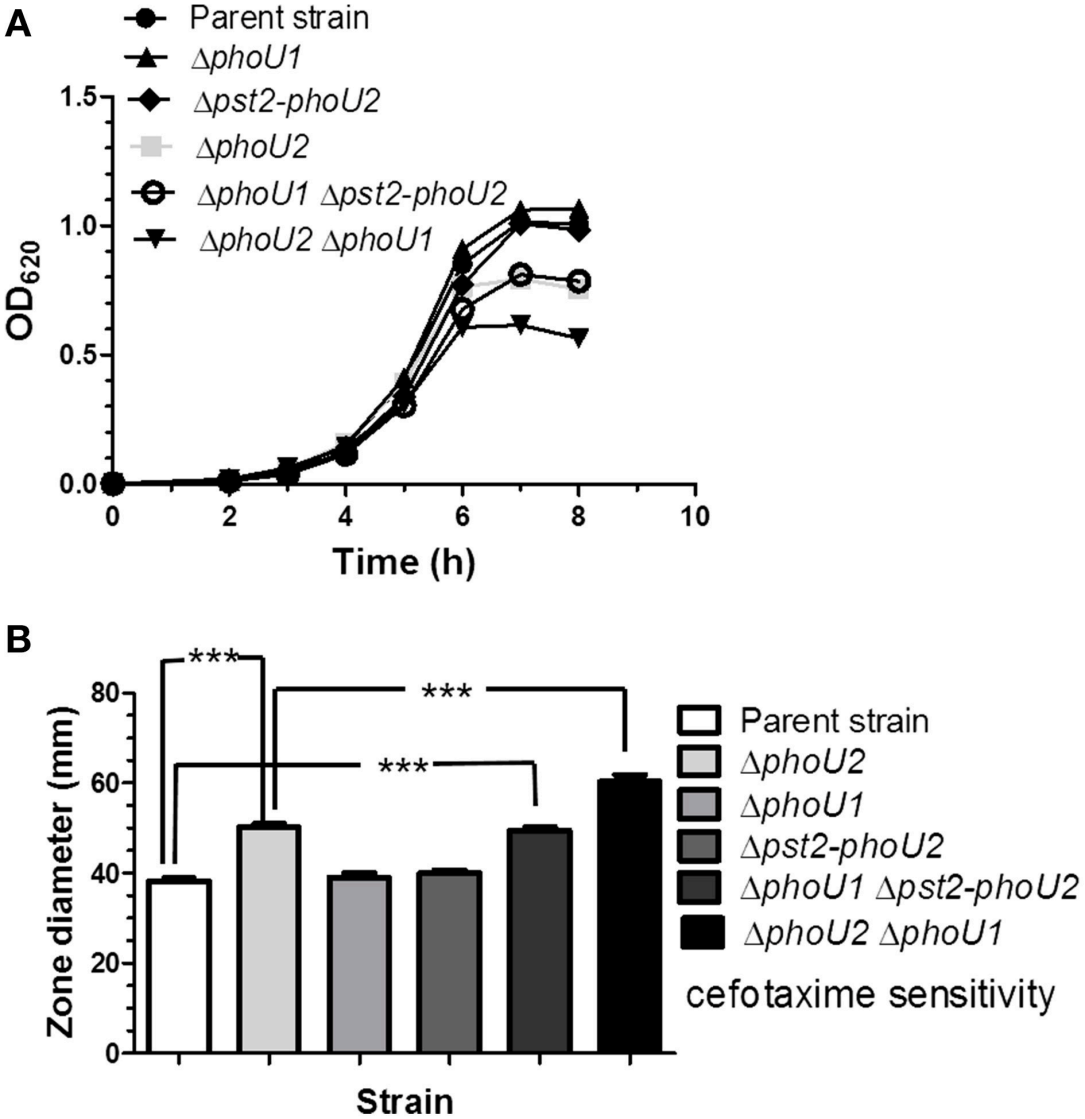
**A Δ*phoU1* Δ*pst2-phoU2* double mutant phenocopies a Δ*phoU2* single mutant for decreased growth yield and increased cefotaxime sensitivity, which are even greater in a Δ*phoU1* Δ*phoU2* double mutant. (A)** Representative growth curves of encapsulated parent strain (IU1781) and Δ*phoU2* (IU6375), Δ*phoU1* (IU6377), Δ*pst2-phoU2* (IU6550), Δ*phoU1* Δ*pst2-phoU2* (IU6612), and Δ*phoU2* Δ*phoU1* (IU6499) mutants in BHI broth. (*n* ≥ 3) Growth yields and rates are quantitated in Table S4. **(B)** Cefotaxime sensitivity assays for encapsulated parent strain (IU1781) and Δ*phoU2* (IU6375), Δ*phoU1* (IU6377), Δ*pst2-phoU2* (IU6550), Δ*phoU1* Δ*pst2-phoU2* (IU6612), and Δ*phoU2* Δ*phoU1* (IU6499) mutants. (*n* ≥ 3); ^***^P < 0.001. See Materials and Methods, the legend to Figure 2, and the text for additional details.

We next tested whether absence of the Pst2 transporter affects *pst1* operon expression in bacteria growing in high P_i_-BHI broth. Surprisingly, relative transcript amounts from the *pst1* operon increased by ≈44 fold in the Δ*pst2 phoU2*^+^, Δ*pst2-phoU2*, and Δ*pst2-phoU2* Δ*phoU1* mutant compared to the parent strain (Table 1, lines 12, 13, and 14). By contrast, *pst2* operon transcription is unchanged in a Δ*pst1 phoU1*^+^ mutant compared to the parent (Table 1, line 15). The similar induction in the Δ*pst2 phoU2*^+^ and Δ*pst2-phoU2* mutants (Table 1, lines 12 and 13) implies that negative regulation of the PnpRS TCS by PhoU2 depends on a functional Pst2 transporter system. Moreover, the similarity of *pst1* induction in the Δ*pst2-phoU2* Δ*phoU1* and other mutants implies that PhoU1 does not directly regulate *pst1* transcription. This conclusion was supported by the similar induction of *pst1* operon transcription in the Δ*phoU2 phoU1*^+^ and Δ*phoU2* Δ*phoU1* mutants (Table 1, lines 2 and 11). Instead, the growth characteristics of these mutants imply that PhoU1 negatively regulates the activity of the Pst1 transporter, in parallel to the negative regulation of the activity of Pst2 by PhoU2 (Figure 5A, Table S4). In BHI broth, the parent, Δ*phoU1*, and Δ*pst2-phoU2* mutant show similar growth and antibiotic sensitivity (Figure 5; Table S4). In contrast, the Δ*phoU2* and Δ*phoU1* Δ*pst2-phoU2* mutants showed reduced growth yield and increased antibiotic sensitivity (Figure 5, Table S4), consistent with increased P_i_ accumulation caused by misregulation of the Pst2 and Pst1 transporters, respectively. This interpretation was further supported by the reduced growth rate and yield and increased antibiotic sensitivity of the Δ*phoU2* Δ*phoU1* mutant compared to the Δ*phoU2* and Δ*pst2-phoU2* Δ*phoU1* mutants (Figure 5; Table S4).

### Either the Pst1 or Pst2 transporter is required in the encapsulated, but not in the unencapsulated, D39 strain

*E. coli* encodes an alternate low-affinity P_i_ uptake (Pit) system that functions in the absence of the high-affinity Pst transporter in high P_i_ conditions (Wanner, 1996; Harris et al., 2001; Hsieh and Wanner, 2010). However, we were unable to construct Δ*pst2* Δ*pst1* mutants in the encapsulated strain of D39 (Table 2, top two sections), where the Δ*nptA* amplicon was used as a positive control in the transformations. Likewise, we were unable to construct a Δ*pst2* Δ*pnpRS* double mutant, where induction of the Pst1 transporter was negated by the absence of the PnpRS TCS (Table 1, top section; Figure 1). As expected, the *cps*^+^ Δ*pnpRS*-*pst1* mutant could be constructed (IU6133), where the Pst2 transporter is functional.

**Table 2:** **The Δ*pst1* Δ*pst2* double mutant cannot be constructed in encapsulated strain D39^a^**.

**Recipient strain^b^**	**Amplicon^c^**	**Number of colonies on transformation plates after ≈20 h^d^**
Δ*pst2 cps*^+^ (encapsulated)	Δ*nptA* (control)	100–150 (*n* = 3)
	Δ*pnpRS*	0–10 (*n* = 3)
	Δ*pst1*	0–4 (*n* = 3)
	Δ*pst1-phoU1*	0 (*n* = 3)
	No DNA (control)	0 (*n* = 3)
Δ*pst1 cps*^+^ (encapsulated)	Δ*nptA* (control)	100–150 (*n* = 3)
	Δ*pst2*	0 (*n* = 3)
	Δ*pst2-phoU2*	0 (*n* = 3)
	No DNA (control)	0 (*n* = 3)
Δ*pst2 cps* mutants (unencapsulated)	Δ*nptA* (control)	100–150 (*n* = 3)
	Δ*pnpRS*	250–300 (*n* = 3)
	Δ*pst1*	100–150 (*n* = 3)
	Δ*pst1-phoU1*	250–300 (*n* = 3)
	No DNA (control)	0 (*n* = 3)
Δ*pst1 cps* mutants (unencapsulated)	Δ*nptA* (control)	100–150 (*n* = 3)
	Δ*pst2*	100–150 (*n* = 3)
	Δ*pst2-phoU2*	150–200 (*n* = 3)
	No DNA (control)	0 (*n* = 3)

a *Transformations were performed as described in Materials and Methods*.

b *Transformations were performed into multiple cps^+^ strains [IU1690 (D39); IU1781 (D39 rpsL1)] and cps mutants [IU1824 (D39 Δcps rpsL1); IU1945 (D39 Δcps); IU3309 (D39 Δcps2E rpsL1)] with similar results. D39 Δcps2E rpsL1 Δpst1 Δpst2 mutants could not be repaired back to cps^+^ (data not shown)*.

c *Amplicons were synthesized as described in Materials and Methods (see Table S2). Amplicons used for transformations contained the P_c_-kanrpsL^+^ or P_c_-erm antibiotic cassette for selection. Transformations with the ΔnptA amplicon or without DNA were the positive or negative control, respectively*.

d *≤ 10 colonies on plates indicates accumulation of unencapsulated suppressor mutants (see text)*.

In all Δ*pst2* Δ*pst1 cps*^+^ transformations, several colonies (< 10) appeared upon prolonged incubation (Table 2, top two sections). These suppressor colonies had a rougher appearance than the smooth colonies of D39 *cps*^+^ strains (data not shown), suggesting that capsule production was lost or reduced in these Δ*pst2* Δ*pst1* transformants. The Quellung test for serotype 2 capsule (see Materials and Methods) confirmed that a suppressor strain (IU6413) had lost its capsule (data not shown). This result suggested that unlike in *cps*^+^ strains, we would be able to construct Δ*cps* Δ*pst1* Δ*pst2* mutants in the D39 genetic background. This hypothesis was confirmed by transformation experiments (Table 2, bottom two sections), and growth experiments showing that Δ*cps* Δ*pst1* Δ*pst2* triple mutants grew comparably to the Δ*cps* single mutant in BHI broth (Figure S5). Thus, we conclude that either the Pst1 or Pst2 P_i_ transporter must be functional in encapsulated D39 strains and that the Δ*pst1* Δ*pst2 cps*^+^ double mutant is not viable.

### NptA is the third P_i_ uptake system that functions in Δ*cps* Δ*pst1* Δ*pst2* mutants

Normal growth of the Δ*cps* Δ*pst1* Δ*pst2* mutant in high P_i_ medium (Figure 5) implies that sufficient P_i_ is being taken up by a third uptake system. BLAST searches did not reveal a close pneumococcal homolog of the Pit symporters of *E. coli* and *B. subtilis*. During these searches, we found another candidate gene, *spd*_*0443*, which encodes a putative Na^+^/P_i_-cotransporter II-like protein. Spd_0443 homologs have been shown to act as P_i_ transporters in mammalian intestines and kidneys (Katai et al., 1999) and in certain bacterial species, such as *V. cholerae* and *Vibrio vulnificus* (Lebens et al., 2002; Staley and Harwood, 2014). Consistent with a role in P_i_ uptake, we could not delete *spd_0443* in a Δ*cps* Δ*pst1* Δ*pst2* mutant in high P_i_ medium (Table 3), but we could delete *spd_0443* in the Δ*pst1* or Δ*pst2* single mutant (Table 2). Thus, the Spd_0443 Na^+^/P_i_-cotransporter likely acts as a third Pi uptake system in *S. pneumoniae*. Because the iron transporter in *S. pneumoniae* is already named “Pit,” we renamed Spd_0443 as NptA (Na^+^-dependent phosphate transporter A), similar to *Vibrio* species (Lebens et al., 2002).

**Table 3:** **NptA (Na^+^/Pi co-transporter) is a third P_i_ uptake system**.

**Recipient strain^a^**	**Amplicon^b^**	**Number of colonies on transformation plates after ≈20 h**
Δ*pst1 cps*	Δ*pnpR* (control)	150–200 (*n* = 3)
	Δ*nptA*	100–150 (*n* = 3)
	No DNA (control)	0 (*n* = 3)
Δ*pst2 cps*	Δ*pnpR* (control)	150–200 (*n* = 3)
	Δ*nptA*	100–150 (*n* = 3)
	No DNA (control)	0 (*n* = 3)
Δ*pst1* Δ*pst2 cps*	Δ*pnpR* (control)	150–200 (*n* = 3)
	Δ*nptA*	0 (*n* = 3)
	No DNA (control)	0 (*n* = 3)

a *Transformations were performed as described in Materials and Methods. Transformations were performed into two cps mutants [IU1824 (D39 Δcps rpsL1); IU3309 (D39 Δcps2E rpsL1)] with similar results*.

b *Amplicons were synthesized as described in Materials and Methods (see Table S2). Amplicons used for transformations contained the P_c_-kanrpsL^+^ or P_c_-erm antibiotic cassette for selection. Transformations with the ΔpnpR amplicon or without DNA were the positive and negative control, respectively*.

### Δ*pst1-phoU1* or Δ*pst2-phoU2* deletion has no effect on growth under low P_i_ conditions

**Figure 6:**
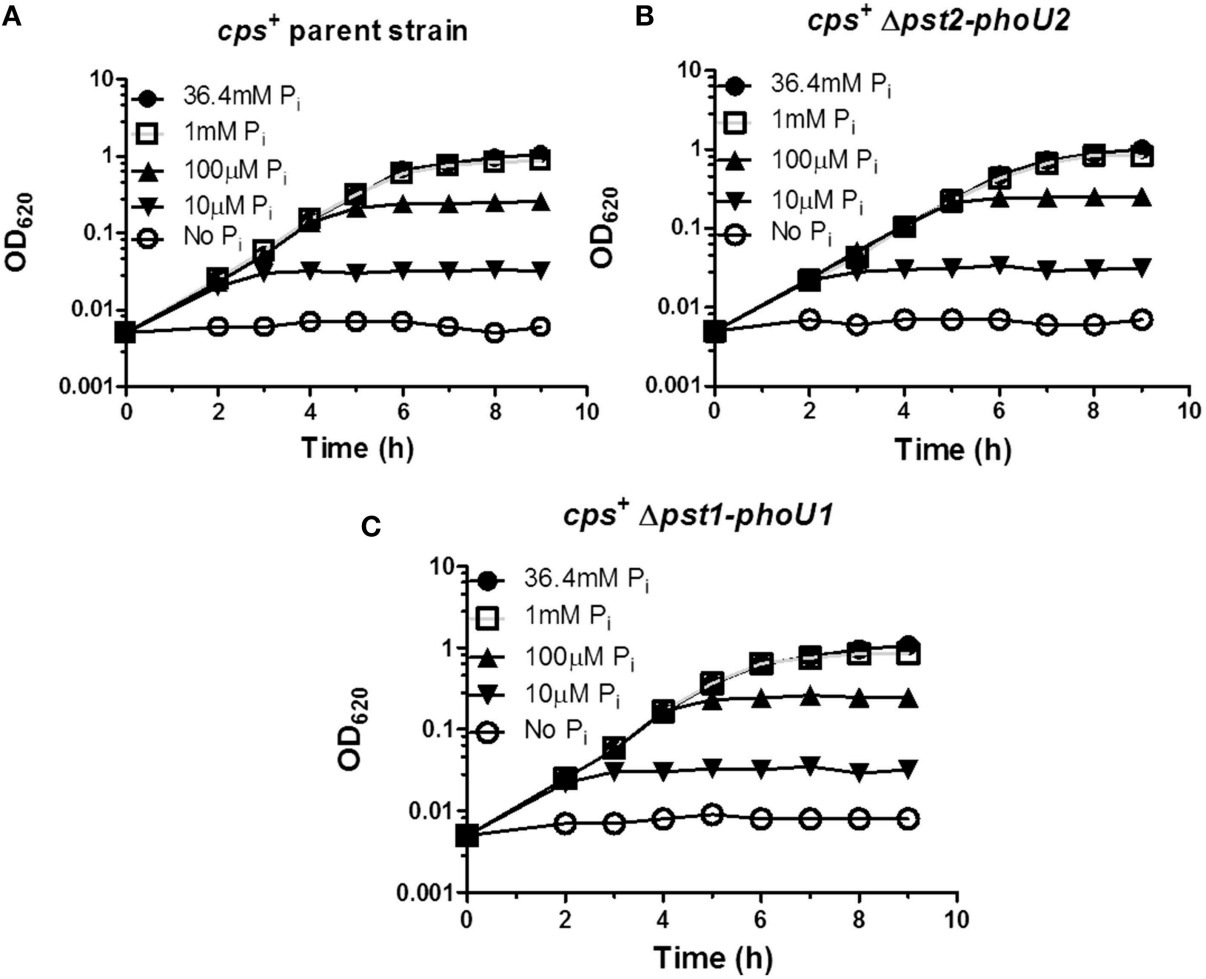
**Deletion of the *pst1-phoU1* or *pst2-phoU2* operon does not affect growth under low P_i_ conditions in mCDM media**. **(A)** Representative growth curves of encapsulated parent strain (IU1781) in mCDM with 36.4, 1 mM, 100, 10 μM, and no P_i_. **(B)** Representative growth curves of a Δ*pst2-phoU2* mutant (IU6550) in mCDM with 36.4, 1 mM, 100, 10 μM, and no P_i_. **(C)** Representative growth curves of a Δ*pst1-phoU1* mutant (IU6830) in mCDM with 36.4, 1 mM, 100, 10 μM, and no P_i_. mCDM with different concentrations of P_i_ was prepared as described in Materials and Methods. Growth curves were determined at least 3X independently.

We next examined the roles of the Pst1 and Pst2 transporters and their regulators (Figure 1) under low P_i_ culture conditions. A previous study used the semi-defined C+Y medium as a low P_i_ condition (Novak et al., 1999). However, encapsulated strains grew in C+Y broth without P_i_ addition (Figure S6), and direct chemical assay (see Materials and Methods; Katewa and Katyare, 2003) showed that C+Y broth (with no added P_i_) contains ≈1.5 mM P_i_ (Figure S7). Therefore, a modified chemically defined medium (mCDM) was used to study low P_i_ culture conditions (see Materials and Methods; Carvalho et al., 2013). The growth yield of the wild-type encapsulated strain was dependent on P_i_ concentration below 1 mM P_i_, with growth detectable down to ≈10 μM and no growth without P_i_ addition (Figure 6A). Growth rates and yields of the Δ*pst2-phoU2* and Δ*pst1*-*phoU1* mutants were similar to those of the parent strain (Figures 6B,C), implying that a functional Pst1 or Pst2 transporter is sufficient for optimal growth under low P_i_ conditions.

**Figure 7:**
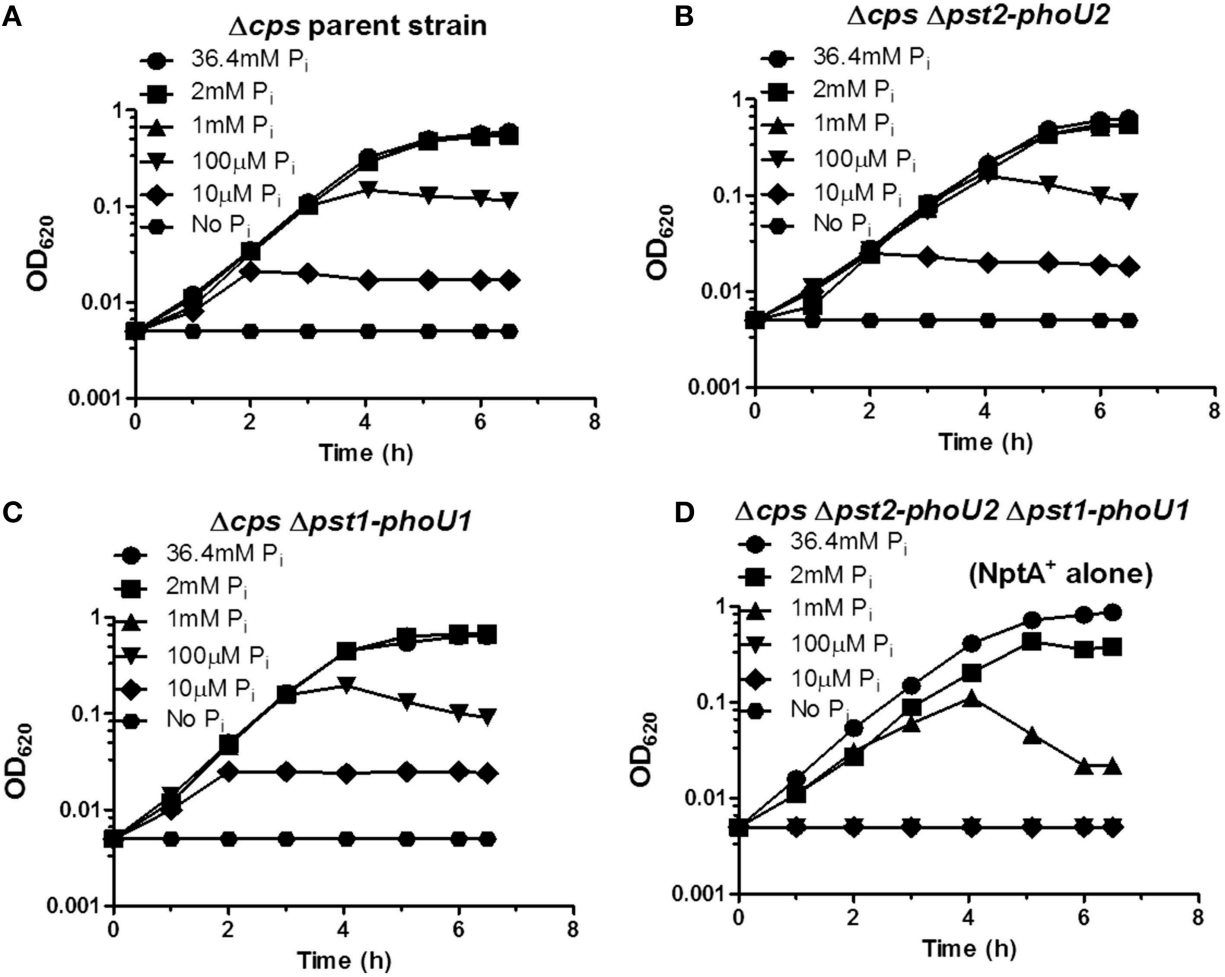
**The NptA Na^+^/P_i_ co-transporter alone cannot provide sufficient P_i_ for long-term growth in mCDM containing moderately high (1 mM) and lower P_i_ concentrations**. **(A)** Representative growth curves of unencapsulated parent strain Δ*cps* (IU1945) in mCDM with 36.4, 2, 1 mM, 100, 10 μM, and no P_i_. **(B)** Representative growth curves of unencapsulated Δ*cps* Δ*pst2-phoU2* mutant (K583) in mCDM with 36.4, 2, 1 mM, 100, 10 μM, and no P_i_. **(C)** Representative growth curves of unencapsulated Δ*cps* Δ*pst1-phoU1* mutant (E595) in mCDM with 36.4, 2, 1 mM, 100, 10 μM, and no P_i_. **(D)** Representative growth curves of unencapsulated Δ*cps* Δ*pst2-phoU2* Δ*pst1-phoU1* mutant (IU5774), which only contains the NptA Na^+^/P_i_ transporter, in mCDM with 36.4, 2, 1 mM, 100, 10 μM, and no P_i_. Growth curves were determined at least 3X independently.

We used unencapsulated (Δ*cps*) mutants to determine the effects of P_i_ concentration when both the Pst1 and Pst2 transporters were absent. Similar to the encapsulated strains (Figure 6), growth yield of the parent, Δ*pst1*-*phoU1*, and Δ*pst2*-Δ*phoU2* single mutants decreased below 1 mM P_i_ and was still detectable at 10 μM P_i_ (Figures 7A–C). By contrast, the growth of the Δ*pst2-phoU2* Δ*pst1-phoU1* double mutant was not fully supported even by 2 mM P_i_, and growth yield showed apparent autolysis in 1 mM P_i_ and no growth with ≥100 μM P_i_ (Figure 7D). We conclude that in low P_i_ conditions, the NptA transporter alone is not sufficient for growth of D39-derived *S. pneumoniae*, consistent with NptA acting as a low-affinity P_i_ transporter compared to Pst1 or Pst2.

### Low P_i_ induces *pst1* operon transcript amount >100X via phosphorylation of the PnpR~P RR

We determined the effect of low P_i_ concentration on *pst1* operon expression in the encapsulated D39 strain. Strains were grown in high-P_i_ (36.4 mM) mCDM, washed, and resuspended in mCDM containing high (36.4 mM) or low (10 μM) P_i_ (see Materials and Methods). After 30 min, samples were taken for qRT-PCR using 16S rRNA as the normalization standard (see Materials and Methods). In all strains tested, P_i_ limitation to 10 μM induced relative *pst1* transcript amount by >100X compared to the wild-type parent strain in high (36.4 mM) P_i_ (Table 4). Similar to strains grown in BHI (Table 1), Δ*phoU2* and Δ*phoU2* Δ*phoU1* mutants showed ≈25X induction of *pst1* operon transcript amount when grown in mCDM containing high P_i_ (Table 4), whereas a Δ*phoU1* mutant showed no increase (Table 4). In these mutants, reduction of P_i_ concentration to 10 μM induced *pst1* transcript amounts by an additional ≈5–100X (Table 4). P_i_ limitation of a Δ*pnpRS* TCS mutant showed no increase in relative *pst1* operon transcript amounts (data not shown), indicating a dependence on the PnpRS TCS for induction of *pst1* operon transcription in low P_i_ media. This conclusion was confirmed by Phos-tag SDS PAGE, which showed that phosphorylation of the PnpR RR (PnpR~P) went from < 1% in mCDM containing 36.4 mM P_i_ to ≈80% following a shift to mCDM lacking P_i_ for 40 min (Figure S8A). We conclude that transcription of pneumococcal *pst1* transporter operon is strongly induced by the PnpR~P RR in low P_i_ conditions.

**Table 4:** **Relative transcript amounts of the *pst1* operon in different mutants in mCDM containing high and low P_i_ concentrations^a^**.

**Strains^b^**	**P_i_ concentration in mCDM**	**Relative transcript amount of *pstS1* operon^c^**
Parent strain (IU1781)	36.4 mM	≡1
	10 μM	209.7 ± 37.9 (*n* = 6) (^***^)^*d*^
Δ*phoU2* (IU6375)	36.4 mM	24.1 ± 3.9 (*n* = 3) (^***^)
	10 μM	201.6 ± 47.6 (*n* = 3) (^***^)
Δ*phoU1* (IU6377)	36.4 mM	1.4 ± 0.1 (*n* = 3) (ns)
	10 μM	145.4 ± 25.3 (*n* = 4) (^***^)
Δ*phoU2* Δ*phoU1* (IU6499)	36.4 mM	28.3 ± 0.5 (*n* = 3) (^***^)
	10 μM	136.3 ± 12.6 (*n* = 3) (^***^)

a *RNA preparation and qRT-PCR from strains in mCDM containing high and low P_i_ concentrations were performed as described in Materials and Methods*.

b *Strains were markerless deletion mutants derived from encapsulated parent strain IU1781 (see Table S1)*.

c *Relative pstS1 gene transcript amount was used to represent pst1 operon expression*.

d ^***^P < 0.001; ns, not significant. P-values were calculated by an unpaired t-test in GraphPad Prism.

### Deletion of *pst1-phoU1* and *pst2-phoU2* reduces the rate of P_i_ uptake by ≈50% in an unencapsulated strain

**Figure 8:**
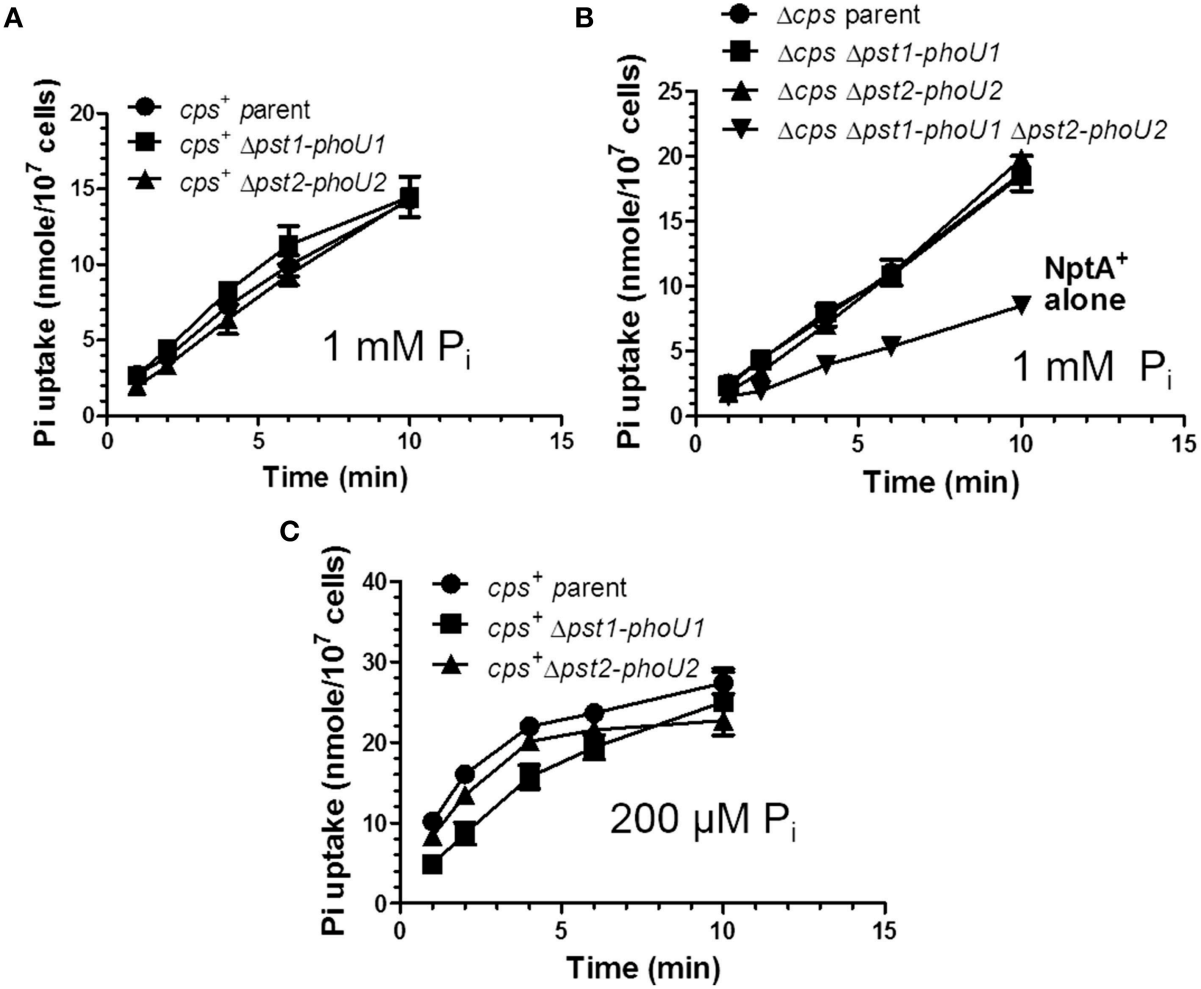
**The Pst1 or Pst2 transporter is sufficient for P_i_ uptake under moderately high P_i_ conditions (1 mM), but the Pst1 transporter is required for optimal P_i_ uptake under low P_i_ conditions (200 μM) following P_i_ limitation**. **(A)** P_i_ uptake assays of encapsulated parent strain (IU1781) and Δ*pst2-phoU2* (IU6550) and Δ*pst1-phoU1* (IU6830) mutants in 1 mM total ^32^P_i_ in mCDM (*n* = 3). P_i_ uptake was determined as the initial rate of ^32^P_i_ incorporation into cells in mCDM in pulse-labeling experiments described in Materials and Methods. See Tables S7 for initial rates of P_i_ uptake (*n* = 3). **(B)** P_i_ uptake assays of unencapsulated parent strain Δ*cps* (IU1945) and Δ*cps* Δ*pst2-phoU2* (K583), Δ*cps* Δ*pst1-phoU1* (E595), and Δ*cps* Δ*pst1-phoU1* Δ*pst2-phoU2* (IU5774) mutants in 1 mM P_i_ mCDM. See Tables S7 for initial rates of P_i_ uptake (*n* = 3). **(C)** P_i_ uptake assay of encapsulated parent strain (IU1781) and Δ*pst2-phoU2* (IU6550) and Δ*pst1-phoU1* (IU6830) mutants in 200 μM total P_i_ mCDM following P_i_ starvation for 1 h as described in Materials and Methods (*n* = 3). Initial rates of Pi uptake were determined with the first min of labeling and are listed in Table S8 (*n* = 3).

Previously, the relative rate of P_i_ uptake was reported to be reduced by ≈30% in a Δ*pstB1* mutant of laboratory strain R6x grown in C+Y medium (no added P_i_) (Novak et al., 1999), which turns out to contain a relatively high (1.5 mM) P_i_ content (above; Figure S7). To the contrary, ^32^P_i_ uptake experiments in encapsulated and unencapsulated D39 mutants lacking the Pst1 (Δ*pst1-phoU1*) or Pst2 (Δ*pst2-phoU2*) transporter showed linear rates of ^32^P_i_ uptake for at least 10 min that were similar to those of the wild-type parent strains in mCDM containing 1 mM total P_i_ (Figures 8A,B, Table S7). R6x is a derivative of R6 (Tiraby and Fox, 1973), which is an unencapsulated derivative of D39 (Lanie et al., 2007). The difference between this and the previous result (Novak et al., 1999) may partly reflect the large number of mutations in the R6 laboratory strain compared to the D39 strain progenitor genetic background (Lanie et al., 2007). In contrast to the single mutants, the unencapsulated Δ*pst1-phoU1* Δ*pst2-phoU2* double mutant showed a significant drop (≈50%) in the rate of P_i_ uptake compared to the wild-type parent strain or the single mutants in mCDM containing 1 mM P_i_ (Figure 8B, Table S7) or in C+Y broth (data not shown). This result implicates both the Pst1 and Pst2 transporters in P_i_ uptake in *S. pneumoniae* D39.

### P_i_ uptake is reduced in a Δ*pst1-phoU1* mutant limited for total P_i_

To determine P_i_ uptake under low P_i_ conditions, we incubated encapsulated parent, Δ*pst1-phoU1*, or Δ*pst2-phoU2* mutants in mCDM lacking P_i_ for 1 h, and then added ^32^P_i_ (total [P_i_] = 200 μM), and sampled P_i_ uptake with time (Figure 8C). Unlike the constant rate of ^32^P uptake observed at 1 mM total P_i_ (Figures 8A,B), P_i_ uptake leveled off after about 5 min in 200 μM P_i_ (Figure 8C). The initial rate of P_i_ uptake by the encapsulated parent strain was markedly greater (≈8X) in 200 μM P_i_ than in 1 mM P_i_ (Figures 8A,C, Tables S7, S8). The initial rate of P_i_ uptake was comparable for the parent strain and the Δ*pst2-phoU2* mutant (Figure 8C, Table S8). In contrast, the initial rate of P_i_ uptake was reduced by ≈2X in the Δ*pst1-phoU1* mutant compared to the parent or Δ*pst2-phoU2* mutant (Figure 8C, Table S8). These results support a primary role for the Pst1 transporter in low-P_i_ conditions.

## Discussion

In this paper we demonstrate for the first time that regulation of the two evolutionarily distinct Pst P_i_ transport systems is linked in several ways in the human commensal and pathogen, *S. pneumoniae*. Niches encountered by pneumococcus in human hosts contain different P_i_ concentrations, including nasal fluid (≈5 mM), saliva (≈1 mM), and serum (≈1 mM) (Bansal, 1990; Wilson, 2005, 2008). Several aspects of this regulatory network are unusual, compared to the usual negative regulation of P_i_ uptake even at moderate P_i_ concentrations in other bacterial species (Figure 1; Hulett, 1993; Hsieh and Wanner, 2010; Botella et al., 2011). The *pst1-phoU1* and *pst2*-*phoU2* operons are completely separated in the pneumococcal chromosome, and although upstream of the *pst1-phoU1* operon, the *pnpRS* operon, which encodes the PnpRS TCS, is independently expressed and not autoregulated (Table 1, Figure 1). Deletion of gene *spd_1614*, which encodes a third putative PhoU-like protein, did not lead to growth phenotypes and was not studied further (data not shown).

Expression of the Pst2 transporter is constitutive under the conditions tested here, including media containing high P_i_ concentrations (Table 1, Table S6, and Figure S3). However, Pst2 uptake of P_i_ is likely negatively regulated by the PhoU2 protein, whose absence causes a drop in growth yield and increased antibiotic sensitivity in mutants growing in high P_i_ medium (Tables S4, S5, Figures 1, 2, and Figure S3). In addition, PhoU2 negatively regulates the transcription activation of the *pst1-phoU1* operon by the phosphorylated PnpR~P RR, such that a Δ*phoU2* deletion mutation leads to a substantial increase in *pst1-phoU1* transcript amounts (Table 1, Figures 1, 3, and Figure S4). The PhoU1 protein, which shares only 34% amino acid identities with PhoU2, does not play a reciprocal role in negatively regulating *pst1-phoU1* transcription (Table 1, lines 3, 11, and 14), but PhoU1 likely negatively regulates P_i_ uptake by the Pst1, but not the Pst2, P_i_ transporter (Figures 1, 4, 5). Thus, the PhoU2 protein can regulate both PnpR phosphorylation level and Pst2 transporter function, similar to the PhoU homolog in *E. coli* (Hsieh and Wanner, 2010; Gardner et al., 2014, 2015). In contrast, PhoU1 function is restricted to regulating transporter but not TCS function, similar to the PhoU in *C. crescentus* (Lubin et al., 2016). *E. coli* PhoU interacts with the PAS domain of the PhoR HK (Gardner et al., 2014, 2015). However, the pneumococcal PnpS HK lacks a recognizable PAS domain, and it remains to be determined whether PhoU2 regulation of PnpR~P levels is through direct interactions with the PnpS HK. Likewise, it is unknown whether PhoU2 interacts with the PstB1 subunit of the Pst1 transporter to exert control over *pst1-phoU1* operon transcription, by analogy to control in *E. coli* (Gardner et al., 2014, 2015). Interactions of PhoU1 or PhoU2 with subunits of the Pst1 or Pst2 transporters also remain to be determined.

The requirement of a functional Pst1 or Pst2 for growth of the encapsulated serotype 2 strain provides a biological rationale for the constitutive expression and function of the Pst2 transporter at high P_i_ concentrations (Table 2) and why *S. pneumoniae* maintains dual P_i_ uptake systems. Capsule is one of the most important factors required for pneumococcal colonization, carriage, and virulence in its human host (Briles et al., 1992; Morona et al., 2004, 2006; Bentley et al., 2006; Hyams et al., 2010). Expression of the Pst1 transporter is strongly induced by low P_i_ conditions (Table 4), and lack of PhoU1 does not change *pst1-phoU1* operon expression in high P_i_ media (Tables 2, 4). These results, and a reduced rate of P_i_ uptake by mutants lacking Pst1 (Figure 8C, Table S8) indicate that Pst1 functions mainly at low P_i_ concentrations. However, encapsulated *S. pneumoniae* strains require high-affinity P_i_ transport even in high-P_i_ media (Table 2), and this P_i_ uptake cannot be provided by the low-affinity NptA Na^+^/P_i_ cotransporter that replaces Pst1 and Pst2 in unencapsulated strains (Figure 8B, Table S7). The reason underlying this link between Pst-mediated P_i_ transport and the maintenance of capsule is not currently clear. A recent report suggests that low P_i_ conditions induce capsule biosynthesis in *M. tuberculosis* (van de Weerd et al., 2016). Overexpression of capsule could alter the metabolism of *S. pneumoniae* thereby inhibiting the growth of *cps*^+^ Δ*pst1* Δ*pst2* mutants and leading to the appearance of spontaneous *cps* mutants (Table 2). However, starvation of the wild-type D39 *cps*^+^ strain for P_i_ for 1 h did not reveal a qualitative change in capsule amount in the Quellung reaction (data not shown). Moreover, pneumococcal capsule biosynthesis is positively regulated by phosphorylation of regulatory protein CpsD (Yother, 2011), and this protein phosphorylation would likely be reduced during P_i_ limitation.

The regulatory pathways that mediate capsule induction in *M. tuberculosis* involve sigma factors and poly-P_i_ kinases (van de Weerd et al., 2016) that are absent from *S. pneumoniae*. As mentioned in the Introduction, Δ*phoU* mutants of other bacteria accumulate poly-P_i_ (Morohoshi et al., 2002; Hirota et al., 2013; Wang et al., 2013; de Almeida et al., 2015; Lubin et al., 2016), possibly because high intracellular Pi concentration disrupt metabolic homeostasis leading to defects in growth accompanied by general antibiotic sensitivity. RNA-Seq results reported here indicate that the pneumococcal Pho regulon contains a limited number of recognizable genes involved in P_i_ accumulation (Table S6). Notably, *S. pneumoniae* serotype 2 strain D39 as well as some other serotype strains, such as TIGR4, lack recognizable homologs of poly-P_i_ kinases Ppk1 and Ppk2 (Zhang et al., 2002) and Mg^2+^-dependent poly-P_i_ exopolyphosphatase Ppx (Akiyama et al., 1993), but do encode a degradative Mn^2+^-dependent, inorganic pyrophosphatase (PpaC) (Lanie et al., 2007); therefore, it is unclear whether these *S. pneumoniae* strains produces poly-P_i_. DAPI staining experiments that revealed poly-P_i_ accumulation in *C. crescentus* Δ*phoU* mutants (Lubin et al., 2016) were inconclusive and did not indicate any differences in staining of the *phoU2*^+^ parent and Δ*phoU2* mutant in high-P_i_ BHI broth (data not shown). Similarly, DAPI-based assays of extracts did not show any difference between the parent and Δ*phoU2* mutant indicative of different poly-Pi amounts (data not shown).

Taken together, our results suggest that *S. pneumoniae* may maintain the regulated Pst1 and constitutive Pst2 P_i_ transport systems as a failsafe to ensure capsule biosynthesis is maintained during variations in P_i_ conditions. Coordination between the two Pst P_i_ transport systems is coordinated by the PhoU2 protein that modulates transcription of the *pst1-phoU1* operon by the PnpRS TCS and separately regulates the function of the Pst2 transporter. Most bacteria encode a single Pst-type P_i_ transporter as part of a Pho regulon that encode numerous phosphate accumulation and sparing proteins (Hulett, 1993; Wanner, 1996; Hsieh and Wanner, 2010). The limited Pho regulon of *S. pneumoniae* may reflect restriction to the human host, where P_i_ is the primary source of phosphorus (Wilson, 2008). Even among the *Streptococcus*, dual Pst systems are limited to only a few species, including *S. pneumoniae, S. pseudopneumoniae, S. dysgalactiae, S. equi* (Group C)*, S. porcinus, S. agalactiae* (Group B), and *S. equinus*, and are absent from the major Viridans and A Groups. Outside of the *Streptococcus*, multiple Pst P_i_ transporters have only been reported in a very limited number of bacteria, including *Synechocystis sp*. PCC 6803 and *M. tuberculosis* (Braibant et al., 1996; Suzuki et al., 2004; Burut-Archanai et al., 2011; Tischler et al., 2013). In *Synechocystis*, low P_i_ conditions activate expression of both of the Pst P_i_ transporters that are present (Suzuki et al., 2004), whereas in *M. tuberculosis one* Pst transporter is activated by low P_i_ and at least one other Pst transporter seems to be constitutively expressed, like Pst2 in *S. pneumoniae* (Tischler et al., 2013).

## Author contributions

JZ, DS, KW, MW contributed to the conception and design of the work; JZ, DS carried out acquisition and analysis of data; JZ, DS, KW, MW interpreted data for the work; JZ, MW drafted and wrote the final version of the paper; JZ, DS, KW, MW approved the final version to be published.

### Conflict of interest statement

The authors declare that the research was conducted in the absence of any commercial or financial relationships that could be construed as a potential conflict of interest.
