# Supplementary material for: Physiological Roles of the Dual Phosphate Transporter Systems in Low and High Phosphate Conditions and in Capsule Maintenance of Streptococcus pneumoniae D39
Source: Front Cell Infect Microbiol. 2016 Jun 20;6:63. doi: 10.3389/fcimb.2016.00063 (PMC4913102; doi:10.3389/fcimb.2016.00063)
Supplement: Supplementary file 1 [file Presentation1.PDF]

SUPPLEMENTAL INFORMATION (SI)

Physiological Roles of the Dual Phosphate Transporter Systems In Low and High Phosphate Conditions and in Capsule Maintenance of *Streptococcus pneumoniae* D39

Jiaqi J. Zheng, Dhriti Sinha, Kyle J. Wayne, and Malcolm E. Winkler

Department of Biology, Indiana University Bloomington, Bloomington, IN 47405, USA

Table S1. *Streptococcus pneumoniae* strains used in this study
Table S2. Oligonucleotide primers used to construct mutants (order follows Table S1)
Table S3. Oligonucleotide primers used for qRT-PCR
Table S4. Growth yields and doubling times of encapsulated parent strain and mutants grown in BHI broth (≈18 mM P_i)
Table S5. A Δ*phoU2* mutant is more sensitive than the parent strain to different classes of antibiotics
Table S6. RNA-Seq results for Δ*phoU2::kanrpsL*$^+$ single and Δ*phoU2::kanrpsL*$^+$ Δ*phoU1::*Pc-*erm* double mutants
Table S7. Initial rates of P_i uptake in mCDM containing a moderately high P_i concentration (1 mM)
Table S8. Initial rates of P_i uptake in the first minute in low P_i condition (200 μM) following 1 h of P_i starvation

SUPPLEMENTAL FIGURE LEGENDS: **Fig. S1-S8**

REFERENCES TO SUPPLEMENTAL INFORMATION

Table S1. *Streptococcus pneumoniae* strains used in this study.

Strain number	Genotype (description)[a]	Antibiotic resistance[b]	Reference or source
K174	D39 Δ*cps* Δ*pnpS*:: P_c-[*kan*^{R}-*rpsL*$^+$] (IU1945 transformed with Δ*pnpS* :: P_c-[*kan*^{R}-*rpsL*$^+$] amplicon)	Kan^R	This study
K176	D39 Δ*cps* Δ*pstC1*:: P_c-[*kan*^{R}-*rpsL*$^+$] (IU1945 transformed with Δ*pstC1* :: P_c-[*kan*^{R}-*rpsL*$^+$] amplicon)	Kan^R	This study
K178	D39 Δ*cps* Δ*pstA1*:: P_c-[*kan*^{R}-*rpsL*$^+$] (IU1945 transformed with Δ*pstA1* :: P_c-[*kan*^{R}-*rpsL*$^+$] amplicon)	Kan^R	This study
K200	D39 Δ*cps* Δ*pstA2*:: P_c-[*kan*^{R}-*rpsL*$^+$] (IU1945 transformed with Δ*pstA2* :: P_c-[*kan*^{R}-*rpsL*$^+$] amplicon)	Kan^R	This study
K202	D39 Δ*cps* Δ*pstC2*:: P_c-[*kan*^{R}-*rpsL*$^+$] (IU1945 transformed with Δ*pstC2* :: P_c-[*kan*^{R}-*rpsL*$^+$] amplicon)	Kan^R	This study
K236	D39 Δ*cps* Δ*pstS1*:: P_c-[*kan*^{R}-*rpsL*$^+$] (IU1945 transformed with Δ*pstS1* :: P_c-[*kan*^{R}-*rpsL*$^+$] amplicon)	Kan^R	This study
K485	D39 Δ*cps* Δ*pstS2*:: P_c-[*kan*^{R}-*rpsL*$^+$] (IU1945 transformed with Δ*pstS2* :: P_c-[*kan*^{R}-*rpsL*$^+$] amplicon)	Kan^R	This study
K522	D39 Δ*cps* Δ*pnpR*:: P_c-[*kan*^{R}-*rpsL*$^+$] (IU1945 transformed with Δ*pnpR*:: P_c-[*kan*^{R}-*rpsL*$^+$] amplicon)	Kan^R	This study
K577	D39 Δ*cps* Δ*pnpRS*:: P_c-[*kan*^{R}-*rpsL*$^+$] (IU1945 transformed with Δ*pnpRS*:: P_c-[*kan*^{R}-*rpsL*$^+$] amplicon)	Kan^R	This study
K579	D39 Δ*cps* Δ*phoU2*:: P_c-[*kan*^{R}-*rpsL*$^+$] (IU1945 transformed with Δ*phoU2* :: P_c-[*kan*^{R}-*rpsL*$^+$] amplicon)	Kan^R	This study
K581	D39 Δ*cps* Δ*pstB2*^{1}:: P_c-[*kan*^{R}-*rpsL*$^+$] (IU1945 transformed with Δ *pstB2*^{1}:: P_c-[*kan*^{R}-*rpsL*$^+$] amplicon)	Kan^R	This study
K583	D39 Δ*cps* Δ*pst2-phoU2* :: P_c-[*kan*^{R}-*rpsL*$^+$] (IU1945 transformed with Δ*pst2-phoU2* :: P_c-[*kan*^{R}-*rpsL*$^+$] amplicon)	Kan^R	This study
K591	D39 Δ*cps* Δ*phoU1*:: P_c-[*kan*^{R}-*rpsL*$^+$] (IU1945 transformed with Δ*phoU2* :: P_c-[*kan*^{R}-*rpsL*$^+$] amplicon)	Kan^R	This study
K593	D39 Δ*cps* Δ*pnpRS-pst1-phoU1* :: P_c-[*kan*^{R}-*rpsL*$^+$] (IU1945 transformed with Δ*pnpRS-pst1-phoU1* :: P_c-[*kan*^{R}-*rpsL*$^+$] amplicon)	Kan^R	This study
K595	D39 Δ*cps* Δ*pst1- phoU1* :: P_c-[*kan*^{R}-	Kan^R	This study

	rpsL^{+}] (IU1945 transformed with Δ*pst1-phoU1* :: P_c-[*kan*^{R}-*rpsL*$^{+}$] amplicon)		
K665	D39 Δ*cps* Δ*pstB1*:: P_c-[*kan*^{R}-*rpsL*$^{+}$] (IU1945 transformed with Δ*pstB1*:: P_c-[*kan*^{R}-*rpsL*$^{+}$] amplicon)	Kan^{R}	This study
K667	D39 Δ*cps* Δ*pst1*:: P_c-[*kan*^{R}-*rpsL*$^{+}$] (IU1945 transformed with Δ*pst1*:: P_c-[*kan*^{R}-*rpsL*$^{+}$] amplicon)	Kan^{R}	This study
K669	D39 Δ*cps* Δ*pstC1-pstB1*:: P_c-[*kan*^{R}-*rpsL*$^{+}$] (IU1945 transformed with Δ*pstC1-pstB1*:: P_c-[*kan*^{R}-*rpsL*$^{+}$] amplicon)	Kan^{R}	This study
K671	D39 Δ*cps* Δ*pstB2*^{2}:: P_c-[*kan*^{R}-*rpsL*$^{+}$] (IU1945 transformed with Δ*pstB2*^{2}:: P_c-[*kan*^{R}-*rpsL*$^{+}$] amplicon)	Kan^{R}	This study
K673	D39 Δ*cps* Δ*pst2*:: P_c-[*kan*^{R}-*rpsL*$^{+}$] (IU1945 transformed with Δ*pst2*:: P_c-[*kan*^{R}-*rpsL*$^{+}$] amplicon)	Kan^{R}	This study
K675	D39 Δ*cps* Δ*pstC2-pstB2*^{2}:: P_c-[*kan*^{R}-*rpsL*$^{+}$] (IU1945 transformed with Δ*pstC2-pstB2*^{2}:: P_c-[*kan*^{R}-*rpsL*$^{+}$] amplicon)	Kan^{R}	This study
K723	D39 Δ*cps* Δ*spd0443*:: P_c-[*kan*^{R}-*rpsL*$^{+}$] (IU1945 transformed with Δ*spd0443*:: P_c-[*kan*^{R}-*rpsL*$^{+}$] amplicon)	Kan^{R}	This study
E577	D39 Δ*cps* Δ*pnpRS*:: P_c-[erm^{R}] (IU1945 transformed with Δ*pnpRS*:: P_c-[erm^{R}] amplicon)	Erm^{R}	This study
E579	D39 Δ*cps* Δ*phoU2*:: P_c-[erm^{R}] (IU1945 transformed with Δ*phoU2*:: P_c-[erm^{R}] amplicon)	Erm^{R}	This study
E583	D39 Δ*cps* Δ*pst2-phoU2*:: P_c-[erm^{R}] (IU1945 transformed with Δ*pst2-phoU2*:: P_c-[erm^{R}] amplicon)	Erm^{R}	This study
E591	D39 Δ*cps* Δ*phoU1*:: P_c-[erm^{R}] (IU1945 transformed with Δ*phoU1*:: P_c-[erm^{R}] amplicon)	Erm^{R}	This study
E593	D39 Δ*cps* Δ*pnpRS-pst1-phoU1*:: P_c-[erm^{R}] (IU1945 transformed with Δ*pnpRS-pst1-phoU1*::P_c-[erm^{R}] amplicon)	Erm^{R}	This study
E595	D39 Δ*cps* Δ*pst1- phoU1*:: P_c-[erm^{R}] (IU1945 transformed with Δ*pst1-phoU1*:: P_c-[erm^{R}] amplicon)	Erm^{R}	This study
E665	D39 Δ*cps* Δ*pstB1*:: P_c-[*Erm*^{R}] (IU1945 transformed with Δ*pstB1*:: P_c-[Erm^{R}] amplicon)	Erm^{R}	This study

E667	D39 Δ*cps* Δ*pst1*:: P_c-[*Erm*^{R}] (IU1945 transformed with Δ*pst1*:: P_c-[*Erm*^{R}] amplicon)	Erm^R	This study
E669	D39 Δ*cps* Δ*pstC1-pstB1*:: P_c-[*Erm*^{R}] (IU1945 transformed with Δ*pstC1-pstB1*:: P_c-[*Erm*^{R}] amplicon)	Erm^R	This study
E671	D39 Δ*cps* Δ*pstB2*^{2}:: P_c-[*Erm*^{R}] (IU1945 transformed with Δ *pstB2*^{2}::P_c-[*Erm*^{R}]amplicon)	Erm^R	This study
E673	D39 Δ*cps* Δ*pst2*::P_c-[*Erm*^{R}] (IU1945 transformed with Δ*pst2*:: P_c-[*Erm*^{R}] amplicon)	Erm^R	This study
E675	D39 Δ*cps* Δ*pstC2-pstB2*^{2}::P_c-[*Erm*^{R}] (IU1945 transformed with Δ*pstC2-pstB2*^{2}:: P_c-[*Erm*^{R}] amplicon)	Erm^R	This study
E723	D39 Δ*cps* Δ*spd0443*::P_c-[*Erm*^{R}] (IU1945 transformed with Δ*spd0443*:: P_c-[*Erm*^{R}] amplicon)	Erm^R	This study
IU1690	D39 (Single colony isolate of serotype 2 strain encapsulated D39 NCTC 7466)	None	NCTC 7466; Lanie *et al.*, 2007
IU1781	D39 *rpsL1* (IU1690 transformed with *pulA-rpsL1-rpsG-fusA* amplicon)	Str^R	Ramos-Montañez *et al.*, 2008
IU1824	D39 *rpsL1* Δ*cps*	Str^R	Lanie *et al.*, 2007
IU1945	D39 Δ*cps*	None	Lanie *et al.*, 2007
IU3309	D39 *rpsL1* Δ*cps2E*	Str^R	Ramos-Montañez *et al.*, 2010
IU5774	D39 Δ*cps* Δ*pst2- phoU2*:: P_c-[*kan*^{R}-*rpsL*$^+$] Δ*pst1-phoU1*:: P_c-[erm^R] (E595 transformed with Δ*pst2-phoU2*:: P_c-[*kan*^{R}-*rpsL*$^+$] amplicon)	Kan^R Erm^R	This study
IU5776	D39 Δ*cps* Δ*pst2-phoU2* :: P_c-[*kan*^{R}-*rpsL*$^+$] Δ*pnpRS-pst1-phoU1*:: P_c-[erm^R] (E593 transformed with Δ*pst2- phoU2* :: P_c-[*kan*^{R}-*rpsL*$^+$] amplicon)	Kan^R Erm^R	This study
IU6121	D39 *rpsL1* Δ*pnpR*:: P_c-[*kan*^{R}-*rpsL*$^+$] (IU1781 transformed with Δ*pnpR* :: P_c-[*kan*^{R}-*rpsL*$^+$] amplicon from K522)	Str^S Kan^R	This study
IU6123	D39 *rpsL1* Δ*pnpS*:: P_c-[*kan*^{R}-*rpsL*$^+$] (IU1781 transformed with Δ*pnpS* :: P_c-[*kan*^{R}-*rpsL*$^+$] amplicon from K174)	Str^R Kan^R	This study
IU6125	D39 *rpsL1* Δ*pstS1*:: P_c-[*kan*^{R}-*rpsL*$^+$] (IU1781 transformed with Δ*pstS1* :: P_c-[*kan*^{R}-*rpsL*$^+$] amplicon from K236)	Str^S Kan^R	This study

IU6127	D39 *rpsL1* Δ*pstC1*:: P_c-[*kan*^{R}-*rpsL*$^+$] (IU1781 transformed with Δ*pstC1*:: P_c-[*kan*^{R}-*rpsL*$^+$] amplicon from K176)	Str^S Kan^R	This study
IU6129	D39 *rpsL1* Δ*pstA1*:: P_c-[*kan*^{R}-*rpsL*$^+$] (IU1781 transformed with Δ*pstA1*:: P_c-[*kan*^{R}-*rpsL*$^+$] amplicon from K178)	Str^S Kan^R	This study
IU6131	D39 *rpsL1* Δ*phoU1*:: P_c-[*kan*^{R}-*rpsL*$^+$] (IU1781 transformed with Δ*phoU1*:: P_c-[*kan*^{R}-*rpsL*$^+$] amplicon from K591)	Str^S Kan^R	This study
IU6133	D39 *rpsL1* Δ*pnpRS-pst1-phoU1* :: P_c-[*kan*^{R}-*rpsL*$^+$] (IU1781 transformed with Δ*pnpRS-pst1-phoU1* :: P_c-[*kan*^{R}-*rpsL*$^+$] amplicon from K593)	Str^S Kan^R	This study
IU6135	D39 *rpsL1* Δ*pnpRS*:: P_c-[*kan*^{R}-*rpsL*$^+$] (IU1781 transformed with Δ*pnpRS*:: P_c-[*kan*^{R}-*rpsL*$^+$] amplicon from K577)	Str^S Kan^R	This study
IU6137	D39 *rpsL1* Δ*pst1- phoU1*:: P_c-[*kan*^{R}-*rpsL*$^+$] (IU1781 transformed with Δ*pst1-phoU1*:: P_c-[*kan*^{R}-*rpsL*$^+$] amplicon from K595)	Str^S Kan^R	This study
IU6139	D39 *rpsL1* Δ*phoU2* :: P_c-[*kan*^{R}-*rpsL*$^+$] (IU1781 transformed with Δ*phoU2* :: P_c-[*kan*^{R}-*rpsL*$^+$] amplicon from K579)	Str^S Kan^R	This study
IU6141	D39 *rpsL1* Δ*pstB2*^{1} :: P_c-[*kan*^{R}-*rpsL*$^+$] (IU1781 transformed with Δ *pstB2*^{1} :: P_c-[*kan*^{R}-*rpsL*$^+$] amplicon from K581)	Str^S Kan^R	This study
IU6143	D39 *rpsL1* Δ*pstA2* :: P_c-[*kan*^{R}-*rpsL*$^+$] (IU1781 transformed with Δ*pstA2* :: P_c-[*kan*^{R}-*rpsL*$^+$] amplicon from K200)	Str^S Kan^R	This study
IU6145	D39 *rpsL1* Δ*pstC2* :: P_c-[*kan*^{R}-*rpsL*$^+$] (IU1781 transformed with Δ*pstC2* :: P_c-[*kan*^{R}-*rpsL*$^+$] amplicon from K202)	Str^S Kan^R	This study
IU6147	D39 *rpsL1* Δ*pstS2* :: P_c-[*kan*^{R}-*rpsL*$^+$] (IU1781 transformed with Δ*pstS2* :: P_c-[*kan*^{R}-*rpsL*$^+$] amplicon from K485)	Str^S Kan^R	This study
IU6149	D39 *rpsL1* Δ*pst2-phoU2* :: P_c-[*kan*^{R}-*rpsL*$^+$] (IU1781 transformed with Δ*pst2-phoU2* :: P_c-[*kan*^{R}-*rpsL*$^+$] amplicon from K583)	Str^S Kan^R	This study
IU6173	D39 *rpsL1* Δ*phoU2*:: P_c-[*kan*^{R}-	Str^S Erm^R	This study

	rpsL$^+$] Δ*phoU1*:: P_c-[*Erm*^{R}] (IU6139 transformed with Δ*phoU1*:: P_c-[*Erm*^{R}] amplicon from E591)	Kan^R	
IU6183	D39 *rpsL1* Δ*phoU2*:: P_c-[*Erm*^{R}] (IU1781 transformed with Δ*phoU2*:: P_c-[*Erm*^{R}] amplicon from E579)	Erm^R	This study
IU6185	D39 *rpsL1* Δ*phoU1*:: P_c-[*Erm*^{R}] (IU1781 transformed with Δ*phoU1*:: P_c-[*Erm*^{R}] amplicon from E591)	Erm^R	This study
IU6187	D39 *rpsL1* Δ*pnpRS-pst1-phoU1*:: P_c-[*Erm*^{R}] (IU1781 transformed with Δ*pnpR-pst1-phoU1*:: P_c-[*Erm*^{R}] amplicon from E593)	Erm^R	This study
IU6189	D39 *rpsL1* Δ*pnpRS*:: P_c-[*Erm*^{R}] (IU1781 transformed with Δ*pnpRS*:: P_c-[*Erm*^{R}] amplicon from E577)	Erm^R	This study
IU6191	D39 *rpsL1* Δ*pst1-phoU1*:: P_c-[*Erm*^{R}] (IU1781 transformed with Δ*pst1-phoU1*:: P_c-[*Erm*^{R}] amplicon from E595)	Erm^R	This study
IU6197	D39 *rpsL1* Δ *phoU1*:: P_c-[*Kan*^{R}-*rpsL*$^+$] Δ *phoU2*:: P_c-[*Erm*^{R}] (IU6131transformed with Δ *phoU2*:: P_c-[*Erm*^{R}] amplicon from E579)	Str^S Erm^R Kan^R	This study
IU6208	D39 *rpsL1* Δ*pst2- phoU2*:: P_c-[*Erm*^{R}] (IU1781 transformed with Δ*pst2-phoU2* :: P_c-[*Erm*^{R}] amplicon from E583)	Erm^R	This study
IU6279	D39 *rpsL1* Δ*pstB1*:: P_c-[*kan*^{R}-*rpsL*$^+$] (IU1781 transformed with Δ*pstB1*:: P_c-[*kan*^{R}-*rpsL*$^+$] amplicon from K665)	Str^S Kan^R	This study
IU6281	D39 *rpsL1* Δ*pst1*:: P_c-[*kan*^{R}-*rpsL*$^+$] (IU1781 transformed with Δ*pst1*:: P_c-[*kan*^{R}-*rpsL*$^+$] amplicon from K667)	Str^S Kan^R	This study
IU6283	D39 *rpsL1* Δ*pstC1-pstB1*:: P_c-[*kan*^{R}-*rpsL*$^+$] (IU1781 transformed with Δ*pstC1- pstB1*:: P_c-[*kan*^{R}-*rpsL*$^+$] amplicon from K669)	Str^S Kan^R	This study
IU6285	D39 *rpsL1* Δ*pstB2*^{2}:: P_c-[*kan*^{R}-*rpsL*$^+$] (IU1781 transformed with Δ*pstB2*^{2}:: P_c-[*kan*^{R}-*rpsL*$^+$] amplicon from K671)	Str^S Kan^R	This study
IU6287	D39 *rpsL1* Δ*pst2*:: P_c-[*kan*^{R}-*rpsL*$^+$] (IU1781 transformed with Δ*pst2*:: P_c-[*kan*^{R}-*rpsL*$^+$] amplicon from K673)	Str^S Kan^R	This study
IU6289	D39 *rpsL1* Δ*pstC2-pstB2*^{2}:: P_c-[*kan*^{R}-*rpsL*$^+$] (IU1781 transformed	Str^S Kan^R	This study

	with Δ*pstC2-pstB2*[2]:: P_c-[*kan*^{R}-*rpsL*$^+$] amplicon from K675)		
IU6356	D39 *rpsL1 pnpR-L-FLAG*[3]-P_c-[*Erm*^{R}] (IU1781 transformed *pnpR-L-FLAG*[3]-P_c-[*Erm*^{R}] amplicon)	Str^R Erm^R	This study
IU6375	D39 *rpsL1 ΔphoU2* (IU6139 transformed *ΔphoU2* amplicon)	Str^R Kan^S	This study
IU6377	D39 *rpsL1 ΔphoU1* (IU6131 transformed *ΔphoU1* amplicon)	Str^R Kan^S	This study
IU6379	D39 *rpsL1 ΔpnpR* (IU6121 transformed *ΔpnpR* amplicon)	Str^R Kan^S	This study
IU6381	D39 *rpsL1 ΔpnpRS* (IU6135 transformed *ΔpnpRS* amplicon)	Str^R Kan^S	This study
IU6389	D39 *rpsL1* Δ*pnpS*:: P_c-[*Erm*^{R}] (IU1781 transformed with Δ*pnpS*:: P_c-[*Erm*^{R}] amplicon from E188)	Str^R Erm^R	This study
IU6397	D39 *rpsL1* Δ*phoU2 bgaA*::*kan-T1T2*-P_{ftsA}-*phoU2* (IU6375 transformed with *bgaA*::*kan-T1T2*-P_{ftsA}-*phoU2* amplicon)	Str^R Kan^R	This study
IU6407	D39 *rpsL1* Δ*pnpRS::* P_c-[*Erm*^{R}] Δ*pst2-phoU2*:: P_c-[*kan*^{R}-*rpsL*$^+$] Δ*cps* (E577 transformed with Δ*pst2-phoU2*:: P_c-[*kan*^{R}-*rpsL*$^+$] amplicon from K583)	Str^S Kan^R Erm^R	This study
IU6409	D39 *rpsL1* Δ*pst1-phoU2*:: P_c-[*Erm*^{R}] Δ*pst2-phoU2*:: P_c-[*kan*^{R}-*rpsL*$^+$] Δ*cps* (E595 transformed with Δ*pst2-phoU2*:: P_c-[*kan*^{R}-*rpsL*$^+$] amplicon from K583)	Str^S Kan^R Erm^R	This study
IU6411	D39 *rpsL1* Δ*pnpRS-pst1-phoU1*:: P_c-[*Erm*^{R}] Δ*pst2-phoU2*:: P_c-[*kan*^{R}-*rpsL*$^+$] Δ*cps* (E593 transformed with Δ*pst2-phoU2*:: P_c-[*kan*^{R}-*rpsL*$^+$] amplicon from K583)	Str^S Kan^R Erm^R	This study
IU6413	D39 *rpsL1* Δ*pst1-phoU1*::P_c-[*Erm*^{R}] Δ*pst2-phoU2*::P_c-[*kan*^{R}-*rpsL*$^+$] suppressor (IU6191 transformed with Δ*pst2-phoU2*:: P_c-[*kan*^{R}-*rpsL*$^+$] amplicon from K583) (**This strain has lost capsule based on Quellung reaction**!)	Str^S Kan^R Erm^R	This study
IU6480	D39 *rpsL1* Δ*phoU2* Δ*phoU1*:: P_c-[*kan*^{R}-*rpsL*$^+$] (IU6375 transformed with Δ*phoU1*:: P_c-[*kan*^{R}-*rpsL*$^+$] amplicon from K591)	Str^S Kan^R	This study
IU6482	D39 *rpsL1* Δ*phoU2* Δ*pstS1*:: P_c-[*kan*^{R}-*rpsL*$^+$] (IU6375 transformed with Δ*pstS1*:: P_c-[*kan*^{R}-*rpsL*$^+$] amplicon from K236)	Str^S Kan^R	This study

IU6484	D39 *rpsL1* Δ*phoU2* Δ*pstC1*:: P_c-[*kan*^{R}-*rpsL*$^+$] (IU6375 transformed with Δ*pstC1*:: P_c-[*kan*^{R}-*rpsL*$^+$] amplicon from K176)	Str^S Kan^R	This study
IU6486	D39 *rpsL1* Δ*phoU2* Δ*pstB1*:: P_c-[*kan*^{R}-*rpsL*$^+$] (IU6375 transformed with Δ*pstB1*:: P_c-[*kan*^{R}-*rpsL*$^+$] amplicon from K665)	Str^S Kan^R	This study
IU6488	D39 *rpsL1* Δ*phoU2* Δ*pst1-phoU1*:: P_c-[*kan*^{R}-*rpsL*$^+$] (IU6375 transformed with Δ*pst1-phoU1*:: P_c-[*kan*^{R}-*rpsL*$^+$] amplicon from K595)	Str^S Kan^R	This study
IU6496	D39 *rpsL1* Δ*pnpS* (IU6123 transformed with Δ*pnpS* amplicon)	Str^R Kan^S	This study
IU6499	D39 *rpsL1* Δ*phoU2* Δ*phoU1* (IU6480 transformed with Δ*phoU1*amplicon)	Str^R Kan^S	This study
IU6514	D39 *rpsL1* Δ*phoU2* Δ*pstS2*:: P_c-[*kan*^{R}-*rpsL*$^+$] (IU6375 transformed with Δ*pstS2*:: P_c-[*kan*^{R}-*rpsL*$^+$] amplicon from K485)	Str^S Kan^R	This study
IU6516	D39 *rpsL1* Δ*phoU2* Δ*pstC2*:: P_c-[*kan*^{R}-*rpsL*$^+$] (IU6375 transformed with Δ*pstC2*:: P_c-[*kan*^{R}-*rpsL*$^+$] amplicon from K202)	Str^S Kan^R	This study
IU6518	D39 *rpsL1* Δ*phoU2* Δ*pstB2*^{1}:: P_c-[*kan*^{R}-*rpsL*$^+$] (IU6375 transformed with Δ*pstB2*^{1}:: P_c-[*kan*^{R}-*rpsL*$^+$] amplicon from K581)	Str^S Kan^R	This study
IU6520	D39 *rpsL1* Δ*phoU2* Δ*pstB2*^{2}:: P_c-[*kan*^{R}-*rpsL*$^+$] (IU6375 transformed with Δ*phoU2* Δ*pstB2*^{2}:: P_c-[*kan*^{R}-*rpsL*$^+$] amplicon)	Str^S Kan^R	This study
IU6523	D39 *rpsL1* Δ*phoU2* Δ*pstA2*::P_c-[*Erm*^{R}] (IU6375 transformed with Δ*pstA2*::P_c-[*Erm*^{R}] amplicon from E213)	Str^R Kan^S Erm^R	This study
IU6525-2	D39 *rpsL1* Δ*phoU2* Δ*pnpRS*:: P_c-[*kan*^{R}-*rpsL*$^+$] (IU6375 transformed with Δ*pstRS*:: P_c-[*kan*^{R}-*rpsL*$^+$] amplicon from K577)	Str^S Kan^R	This study
IU6527	D39 *rpsL1* Δ*phoU2* Δ*pnpR*:: P_c-[*kan*^{R}-*rpsL*$^+$] (IU6375 transformed with Δ*pnpR*:: P_c-[*kan*^{R}-*rpsL*$^+$] amplicon from K522)	Str^S Kan^R	This study
IU6529	D39 *rpsL1* Δ*phoU2* Δ*pnpS*:: P_c-[*kan*^{R}-*rpsL*$^+$] (IU6375 transformed with Δ*pnpS*:: P_c-[*kan*^{R}-*rpsL*$^+$] amplicon from K174)	Str^S Kan^R	This study
IU6531	D39 *rpsL1* Δ*phoU2* Δ*pst1*:: P_c-	Str^S Kan^R	This study

	[*kan*^{R}-*rpsL*$^+$] (IU6375 transformed with Δ*pst1*:: P_c-[*kan*^{R}-*rpsL*$^+$] amplicon from K667)		
IU6533	D39 *rpsL1* Δ*phoU2* Δ*pst2*::P_c-[*kan*^{R}-*rpsL*$^+$] (IU6375 transformed with Δ*pst2*:: P_c-[*kan*^{R}-*rpsL*$^+$] amplicon from K673)	Str^S Kan^R	This study
IU6550	D39 *rpsL1* Δ*pst2-phoU2* (IU6149 transformed with Δ*pst2-phoU2* amplicon)	Str^R Kan^S	This study
IU6561	D39 *rpsL1* Δ*pst2-phoU2*:: P_c-[*Erm*^{R}] Δ*pnpRS*:: P_c-[*kan*^{R}-*rpsL*$^+$] Δcps (E583 transformed with Δ*pnpRS*:: P_c-[*kan*^{R}-*rpsL*$^+$] amplicon from K577)	Str^S Kan^R Erm^R	This study
IU6573	D39 *rpsL1* Δ*phoU2* Δ*pnpR* (IU6527 transformed with Δ*pnpR* amplicon)	Str^R Kan^S	This study
IU6575	D39 *rpsL1* Δ*phoU2* Δ*pnpRS* (IU6527 transformed with Δ*pnpRS* amplicon)	Str^R Kan^S	This study
IU6583	D39 *rpsL1* Δ*phoU2*:: P_c-[*kan*^{R}-*rpsL*$^+$] Δ*pnpS* (IU6496 transformed with Δ*phoU2*:: P_c-[*kan*^{R}-*rpsL*$^+$] amplicon from K579)	Str^S Kan^R	This study
IU6595	D39 *rpsL1* Δ*phoU2* Δ*pnpS* (IU6583 transformed with Δ*phoU2* amplicon)	Str^R Kan^S	This study
IU6597	D39 *rpsL1* Δ*pst2-phoU2* Δ*phoU1*:: P_c-[*kan*^{R}-*rpsL*$^+$] (IU6550 transformed with Δ*phoU1*:: P_c-[*kan*^{R}-*rpsL*$^+$] amplicon from K591)	Str^S Kan^R	This study
IU6599	D39 *rpsL1* Δ*pst2*:: P_c-[*kan*^{R}-*rpsL*$^+$] Δ*phoU1* (IU6377 transformed with Δ*pst2*:: P_c-[*kan*^{R}-*rpsL*$^+$] amplicon from K673)	Str^S Kan^R	This study
IU6608	D39 *rpsL1* Δ*pst2* Δ*phoU1* (IU6599 transformed with Δ*pst2* amplicon)	Str^R Kan^S	This study
IU6610	D39 *rpsL1* Δ*pst2* (IU6287 transformed with Δ*pst2* amplicon)	Str^R Kan^S	This study
IU6612	D39 *rpsL1* Δ*pst2-phoU2* Δ*phoU1* (IU6597 transformed with Δ*phoU1* amplicon)	Str^R Kan^S	This study
IU6638	D39 *rpsL1* Δ*pst1* (IU6281 transformed with Δ*pst1* amplicon)	Str^R Kan^S	This study
IU6660	D39 *rpsL1* Δ*pst1* Δ*phoU2*:: P_c-[*kan*^{R}-*rpsL*$^+$] (IU6638 transformed with Δ*phoU2*:: P_c-[*kan*^{R}-*rpsL*$^+$] amplicon from K579)	Str^S Kan^R	This study
IU6664	D39 *rpsL1* Δ*pst1* Δ*phoU2* (IU6660 transformed with Δ*phoU2* amplicon)	Str^R Kan^S	This study

IU6682	D39 *rpsL1* Δ*phoU2 pnpR-L-FLAG*^{3}-P$_{c}$-[*Erm*^{R}] (IU6375 transformed with *pnpR-L-FLAG*^{3}- P$_{c}$-[*Erm*^{R}] amplicon)	Str^{R} Kan^{S} Erm^{R}	This study
IU6684	D39 *rpsL1* Δ*phoU2 pnpR-F* (IU6527 transformed with *pnpR-F* amplicon)	Str^{R} Kan^{S}	This study
IU6687	D39 *rpsL1* Δ*phoU2 pnpR-L-FLAG*^{3} (IU6527 transformed with *pnpR-L-FLAG*^{3} amplicon)	Str^{R} Kan^{S}	This study
IU6689	D39 *rpsL1 pnpR-L-FLAG*^{3} (IU6121 transformed with *pnpR-L-FLAG*^{3} amplicon)	Str^{R} Kan^{S}	This study
IU6707	D39 *rpsL1* Δ*pst2*:: P$_{c}$-[*kan*^{R}-*rpsL*$^{+}$] *pnpR-L-FLAG*^{3} (IU6689 transformed with Δ*pst2*:: P$_{c}$-[*kan*^{R}-*rpsL*$^{+}$] amplicon from K673)	Str^{S} Kan^{R}	This study
IU6709	D39 *rpsL1* Δ*pst2-phoU2*:: P$_{c}$-[*kan*^{R}-*rpsL*$^{+}$] *pnpR-L-FLAG*^{3} (IU6689 transformed with Δ*pst2-phoU2*:: P$_{c}$-[*kan*^{R}-*rpsL*$^{+}$] amplicon from K583)	Str^{S} Kan^{R}	This study
IU6730	D39 *rpsL1* Δ*pst2 pnpR-L-FLAG*^{3} (IU6707 transformed with Δ*pst2* amplicon)	Str^{R} Kan^{S}	This study
IU6732	D39 *rpsL1* Δ*pst2-phoU2 pnpR-L-FLAG*^{3} (IU6709 transformed with Δ*pst2-phoU2* amplicon)	Str^{R} Kan^{S}	This study
IU6820	D39 *rpsL1* Δ*phoU1* Δ*pnpR*::P$_{c}$-[*kan*^{R}-*rpsL*$^{+}$] (IU6377 transformed with Δ*pnpR*:: P$_{c}$-[*kan*^{R}-*rpsL*$^{+}$] amplicon from K522)	Str^{S} Kan^{R}	This study
IU6822	D39 *rpsL1* Δ*cps* Δ*pst1*:: P$_{c}$-[*kan*^{R}-*rpsL*$^{+}$] (IU1824 transformed with Δ*pst1*:: P$_{c}$-[*kan*^{R}-*rpsL*$^{+}$] amplicon from K667)	Str^{S} Kan^{R}	This study
IU6824	D39 *rpsL1* Δ*cps* Δ*pst2*:: P$_{c}$-[*kan*^{R}-*rpsL*$^{+}$] (IU1824 transformed with Δ*pst2*:: P$_{c}$-[*kan*^{R}-*rpsL*$^{+}$] amplicon from K673)	Str^{S} Kan^{R}	This study
IU6826	D39 *rpsL1* Δ*cps2E* Δ*pst1*:: P$_{c}$-[*kan*^{R}-*rpsL*$^{+}$] (IU3309 transformed with Δ*pst1*:: P$_{c}$-[*kan*^{R}-*rpsL*$^{+}$] amplicon from K667)	Str^{S} Kan^{R}	This study
IU6828	D39 *rpsL1* Δ*cps2E* Δ*pst2*:: P$_{c}$-[*kan*^{R}-*rpsL*$^{+}$] (IU3309 transformed with Δ*pst2*:: P$_{c}$-[*kan*^{R}-*rpsL*$^{+}$] amplicon from K673)	Str^{S} Kan^{R}	This study
IU6830	D39 *rpsL1* Δ*pst1-phoU1* (IU6137 transformed with *pst1-phoU1* amplicon)	Str^{R} Kan^{S}	This study

IU6846	D39 *rpsL1* Δ*cps* Δ*pst1*:: P_c-[*kan*^{R}-*rpsL*$^+$] Δ*pst2*:: P_c-[*Erm*^{R}] (IU6822 transformed with Δ*pst2*:: P_c-[*Erm*^{R}] amplicon from E673)	Str^S Kan^R Erm^R	This study
IU6848	D39 *rpsL1* Δ*cps* Δ*pst2*:: P_c-[*kan*^{R}-*rpsL*$^+$] Δ*pst1*:: P_c-[*Erm*^{R}] (IU6824 transformed with Δ*pst1*:: P_c-[*Erm*^{R}] amplicon from E667)	Str^S Kan^R Erm^R	This study
IU6850	D39 *rpsL1* Δ*cps2E* Δ*pst1*:: P_c-[*kan*^{R}-*rpsL*$^+$] Δ*pst2*:: P_c-[*Erm*^{R}] (IU6826 transformed with Δ*pst2*:: P_c-[*Erm*^{R}] amplicon from E673)	Str^S Kan^R Erm^R	This study
IU6852	D39 *rpsL1* Δ*cps2E* Δ*pst2*:: P_c-[*kan*^{R}-*rpsL*$^+$] Δ*pst1*:: P_c-[*Erm*^{R}] (IU6828 transformed with Δ*pst1*:: P_c-[*Erm*^{R}] amplicon from E667)	Str^S Kan^R Erm^R	This study
IU6854	D39 *rpsL1* Δ*phoU2* Δ*pst1-phoU1* (IU6488 transformed with Δ*pst1-phoU1* amplicon)	Str^R Kan^S	This study
IU6868	D39 *rpsL1* Δ*phoU1*::P_c-[*kan*^{R}-*rpsL*$^+$] *pnpR-L-FLAG^3* (IU6689 transformed with Δ*phoU1*:: P_c-[*kan*^{R}-*rpsL*$^+$] amplicon from K591)	Str^S Kan^R	This study
IU6876	D39 *rpsL1* Δ*phoU1* *pnpR-L-FLAG^3* (IU6868 transformed with Δ*phoU1* amplicon)	Str^R Kan^S	This study
IU6904	D39 *rpsL1* Δ*phoU2* *pnpR-L-FLAG^3* *bgaA*::*kan-T1T2-*P_{ftsA}*-phoU2* (IU6687 transformed with *bgaA*::*kan-T1T2-PftsA-phoU2* amplicon)	Str^R Kan^R	This study
IU6905	D39 *rpsL1* Δ*cps2E* Δ*pst1* Δ*pst2*:: P_c-[*Erm*^{R}] (IU6850 transformed with Δ*pst1* amplicon)	Str^R Kan^S Erm^R	This study
IU6907	D39 *rpsL1* Δ*cps2E* Δ*pst2* Δ*pst1*:: P_c-[*Erm*^{R}] (IU6852 transformed with Δ*pst2* amplicon)	Str^R Kan^S Erm^R	This study
IU6911	D39 *rpsL1* Δ*phoU2* Δ*phoU1*::P_c-[*kan*^{R}-*rpsL*$^+$] *pnpR-L-FLAG^3* (IU6687 transformed with Δ*phoU1*:: P_c-[*kan*^{R}-*rpsL*$^+$] amplicon from K591)	Str^S Kan^R	This study
IU6913	D39 *rpsL1* Δ*pst2* Δ*phoU1*::P_c-[*kan*^{R}-*rpsL*$^+$] *pnpR-L-FLAG^3* (IU6730 transformed with Δ*phoU1*:: P_c-[*kan*^{R}-*rpsL*$^+$] amplicon from K591)	Str^S Kan^R	This study
IU6915	D39 *rpsL1* Δ*pst2-phoU2* Δ*phoU1*::P_c-[*kan*^{R}-*rpsL*$^+$] *pnpR-L-FLAG^3* (IU6732 transformed with Δ*phoU1*:: P_c-[*kan*^{R}-*rpsL*$^+$] amplicon	Str^S Kan^R	This study

	from K591)		
IU6935	D39 *rpsL1* Δ*phoU2* Δ*phoU1 pnpR-L-FLAG*[3] (IU6911 transformed with Δ*phoU1* amplicon)	Str^R Kan^S	This study
IU6937	D39 *rpsL1* Δ*pst2* Δ*phoU1 pnpR-L-FLAG*[3] (IU6913 transformed with Δ*pst2* amplicon)	Str^R Kan^S	This study
IU6940	D39 *rpsL1* Δ*pst2-phoU2* Δ*phoU1 pnpR-L-FLAG*[3] (IU6915 transformed with Δ*pst2-phoU2* amplicon)	Str^R Kan^S	This study
IU7002	D39 *rpsL1* Δ*cps2E* Δ*pst1* Δ*pst2*::P$_c$-[*kan*^{R}-*rpsL*$^+$] (IU6905 transformed with Δ*pst2*::P$_c$-[*kan*^{R}-*rpsL*$^+$] amplicon from K673)	Str^S Kan^R	This study
IU7004	D39 *rpsL1* Δ*cps2E* Δ*pst2* Δ*pst1*::P$_c$-[*kan*^{R}-*rpsL*$^+$] (IU6907 transformed with Δ*pst1*::P$_c$-[*kan*^{R}-*rpsL*$^+$] amplicon from K723)	Str^S Kan^R	This study
IU7024	D39 *rpsL1* Δ*cps2E* Δ*pst1* Δ*pst2* (IU7002 transformed with Δ*pst2* amplicon)	Str^R Kan^S	This study
IU7026	D39 *rpsL1* Δ*cps* Δ*pst1* Δ*pst2*:: P$_c$-[*Erm*^{R}] (IU6846 transformed with Δ*pst1* amplicon)	Str^R Kan^S Erm^R	This study
IU7038	D39 *rpsL1* Δ*cps* Δ*pst2* Δ*pst1*:: P$_c$-[*Erm*^{R}] (IU6848 transformed with Δ*pst2* amplicon)	Str^R Kan^S Erm^R	This study
IU7040	D39 *rpsL1* Δ*spd0443*:: P$_c$-[*kan*^{R}-*rpsL*$^+$] (IU1781 transformed with Δ*spd0443*:: P$_c$-[*kan*^{R}-*rpsL*$^+$] amplicon from K723)	Str^S Kan^R	This study
IU7042	D39 *rpsL1* Δ*spd0443*::P$_c$-[*Erm*^{R}] (IU1781 transformed with Δ*spd0443*:: P$_c$-[*Erm*^{R}] amplicon from E723)	Str^R Erm^R	This study
IU7044	D39 *rpsL1* Δ*pst2-phoU2* Δ*spd0443*::P$_c$-[*Erm*^{R}] (IU6550 transformed with Δ*spd0443*::P$_c$-[*Erm*^{R}] amplicon from E723)	Str^R Erm^R	This study
IU7046	D39 *rpsL1* Δ*pst1-phoU1* Δ*spd0443*::P$_c$-[*Erm*^{R}] (IU6830 transformed with Δ*spd0443*::P$_c$-[*Erm*^{R}] amplicon from E723)	Str^R Erm^R	This study
IU7050	D39 *rpsL1* Δ*cps* Δ*pst1* Δ*pst2*::P$_c$-[*kan*^{R}-*rpsL*$^+$] (IU7026 transformed with Δ*pst2*::P$_c$-[*kan*^{R}-*rpsL*$^+$] amplicon from K673)	Str^S Kan^R	This study
IU7052	D39 *rpsL1* Δ*cps* Δ*pst2* Δ*pst1*::P$_c$-[*kan*^{R}-*rpsL*$^+$] (IU7038 transformed	Str^S Kan^R	This study

	with Δ*pst1*::P_c-[*kan*^{R}-*rpsL*$^+$] amplicon from K723)		
IU7080	D39 Δ*pst1- phoU1*:: P_c-[*kan*^{R}-*rpsL*$^+$] (IU1690 transformed with Δ*pst1-phoU1* :: P_c-[*kan*^{R}-*rpsL*$^+$] amplicon from K595)	Kan^R	This study
IU7082	D39 Δ*pst2- phoU2*:: P_c-[*kan*^{R}-*rpsL*$^+$] (IU1690 transformed with Δ*pst2-phoU2* :: P_c-[*kan*^{R}-*rpsL*$^+$] amplicon from K583)	Kan^R	This study
IU7084	D39 Δ*pst1*:: P_c-[*kan*^{R}-*rpsL*$^+$] (IU1690 transformed with Δ*pst1*:: P_c-[*kan*^{R}-*rpsL*$^+$] amplicon from K667)	Kan^R	This study
IU7086	D39 Δ*pst2*:: P_c-[*kan*^{R}-*rpsL*$^+$] (IU1690 transformed with Δ*pst2*:: P_c-[*kan*^{R}-*rpsL*$^+$] amplicon from K673)	Kan^R	This study
IU7088	D39 Δ*pnpRS*:: P_c-[*kan*^{R}-*rpsL*$^+$] (IU1690 transformed with Δ*pnpRS* :: P_c-[*kan*^{R}-*rpsL*$^+$] amplicon from 577)	Kan^R	This study
IU7090	D39 Δ*pst1- phoU1*:: P_c-[*kan*^{R}-*rpsL*$^+$] (IU1690 transformed with Δ*pst1-phoU1* :: P_c-[*kan*^{R}-*rpsL*$^+$] amplicon)	Kan^R	This study
IU7130	D39 *rpsL1* Δ*cps* Δ*pst1* Δ*pst2* (IU7050 transformed with Δ*pst2* amplicon)	Str^R Kan^S	This study
IU7311	D39 *rpsL1* Δ*spd0443* (IU7040 transformed with Δ*spd0443* amplicon)	Str^R Kan^S	This study

[a]Primers used to synthesize fusion amplicons are listed in Table S2. FLAG-tag fusions ((C)-FLAG) were made to the carboxyl-ends (C) of reading frames. The amino acid sequence for the FLAG epitope is DYKDDDDK (Wayne et al.; Ramos-Montanez et al., 2008; Wayne et al., 2010). aa = amino acids.

[b]Antibiotic resistance markers: Kan^R, kanamycin; Str^R, streptomycin, Erm^R, erythromycin. Concentrations of antibiotics used for *S. pneumoniae* strains: 250 µg Str per mL; 250 µg Kan per mL; 0.3 µg Erm per mL.

Table S2. Oligonucleotide primers used to construct mutants (order follows Table S1)

Primer	Sequence (5' to 3')	Template[a]	Amplicon Product
**For construction of K174 (Δ*cps* Δ*pnpS*::P_c-[*kan*^{R}-*rpsL*$^+$])**			
P448	ACTACGAAGCAGTCACTTTAGGCCTGGACA	D39	5' flanking fragment
P450	CATTATCCATTAAAAATCAAACGGATCCTA TGCAATCAGAATGAGGCTAACACTTAG		
Kan rpsL forward	TAGGATCCGTTTGATTTTTAATGGATAATG	P_c-[*kan*^{R}-*rpsL*$^+$] cassette[b]	P_c-[*kan*^{R}-*rpsL*$^+$]
Kan rpsL reverse	GGGCCCCTTTCCTTATGCTTTTG		
P451	CAAAAGCATAAGGAAAGGGGCCCCTTGGC AGAGGCAGTTGCTTCAC	D39	3' flanking fragment
P449	GTGACCACTTGTTGACCTTGTTTGGAGTGG		
**For construction of K176 (Δ*cps* Δ*pstC1*::P_c-[*kan*^{R}-*rpsL*$^+$])**			
P452	TCAGCTGCTTCCAAACAGTCAGCTTCAGGA	D39	5' flanking fragment
P454	CATTATCCATTAAAAATCAAACGGATCCTA TGTTGCACTCATGAAAAAAATTGCC		
Kan rpsL forward	TAGGATCCGTTTGATTTTTAATGGATAATG	P_c-[*kan*^{R}-*rpsL*$^+$] cassette[c]	P_c-[*kan*^{R}-*rpsL*$^+$]
Kan rpsL reverse	GGGCCCCTTTCCTTATGCTTTTG		
P455	CAAAAGCATAAGGAAAGGGGCCCCTTATTC TCTTGATTAATGCCTACTTTGCCT	D39	3' flanking fragment
P453	ACGGCCTGAAGACATGAGACTATTTGGCGT		
**For construction of K178 (Δ*cps* Δ*pstA1*::P_c-[*kan*^{R}-*rpsL*$^+$])**			
P457	AAGAAGGCAAGTAGGGAGGTGTCGTATCTC	D39	5' flanking fragment
P459	CATTATCCATTAAAAATCAAACGGATCCTA AGAGCCAAAGGTTAAAGCTGAAAAACA		
Kan rpsL forward	TAGGATCCGTTTGATTTTTAATGGATAATG	P_c-[*kan*^{R}-*rpsL*$^+$] cassette[c]	P_c-[*kan*^{R}-*rpsL*$^+$]
Kan rpsL reverse	GGGCCCCTTTCCTTATGCTTTTG		
P460	CAAAAGCATAAGGAAAGGGGCCCTTAATG ATAAATACTCTATCAAGCTTATTATCTCGAA	D39	3' flanking fragment
P458	CCCATTTGGTGTAACTGTTCTTCGTCAGGG		
**For construction of K200 (Δ*cps* Δ*pstA2*::P_c-[*kan*^{R}-*rpsL*$^+$])**			
P501	TCTCAGCCCTTATCGCAACACCCTTTGCTA	D39	5' flanking fragment
P503	CATTATCCATTAAAAATCAAACGGATCCTA GATGCTAGCAATCGTATAGAGGACAGC		
Kan rpsL forward	TAGGATCCGTTTGATTTTTAATGGATAATG	P_c-[*kan*^{R}-*rpsL*$^+$] cassette[c]	P_c-[*kan*^{R}-*rpsL*$^+$]
Kan rpsL reverse	GGGCCCCTTTCCTTATGCTTTTG		
P504	CAAAAGCATAAGGAAAGGGGCCCTTTAACT TTGGAGCTCGTAAGTTCGGA	D39	3' flanking fragment
P502	TTCCCTGATCCAGATGGACCAATCAAGGCT		

**For construction of K202 (Δ*cps* Δ*pstC2*::P_c-[*kan*^{R}-*rpsL*$^+$])**			
P448	ACTACGAAGCAGTCACTTTAGGCCTGGACA	D39	5’ flanking fragment
P450	CATTATCCATTAAAAATCAAACGGATCCTA TGCAATCAGAATGAGGCTAACACTTAG		
Kan rpsL forward	TAGGATCCGTTTGATTTTTAATGGATAATG	P_c-[*kan*^{R}-*rpsL*$^+$] cassette[c]	P_c-[*kan*^{R}-*rpsL*$^+$]
Kan rpsL reverse	GGGCCCCTTTCCTTATGCTTTTG		
P451	CAAAAGCATAAGGAAAGGGGCCCCTTGGC AGAGGCAGTTGCTTCAC	D39	3’ flanking fragment
P449	GTGACCACTTGTTGACCTTGTTTGGAGTGG		
**For construction of K236 (Δ*cps* Δ*pstS1*::P_c-[*kan*^{R}-*rpsL*$^+$])**			
P567	ACAGCTAAAGGTGCAGGTGCCAGATGATGT	D39	5’ flanking fragment
P569	CATTATCCATTAAAAATCAAACGGATCCTA AACAAGCCCAAATCCTGATAAGCCAA		
Kan rpsL forward	TAGGATCCGTTTGATTTTTAATGGATAATG	P_c-[*kan*^{R}-*rpsL*$^+$] cassette[c]	P_c-[*kan*^{R}-*rpsL*$^+$]
Kan rpsL reverse	GGGCCCCTTTCCTTATGCTTTTG		
P570	CAAAAGCATAAGGAAAGGGGCCCATGGCT GAACTTGCAGACGTTTTTAGTG	D39	3’ flanking fragment
P568	AGTCCACTTGGAATAATCGGCTGGTTGCCT		
**For construction of K485 (Δ*cps* Δ*pstS2*::P_c-[*kan*^{R}-*rpsL*$^+$])**			
P1055	ACAAGTTGCCTTGTGGCAGGAATTTGCCCA	D39	5’ flanking fragment
P1057	CATTATCCATTAAAAATCAAACGGATCCTA TAAACAAGCCGTCAGCCAAAAAGC		
Kan rpsL forward	TAGGATCCGTTTGATTTTTAATGGATAATG	P_c-[*kan*^{R}-*rpsL*$^+$] cassette[c]	P_c-[*kan*^{R}-*rpsL*$^+$]
Kan rpsL reverse	GGGCCCCTTTCCTTATGCTTTTG		
P1058	CAAAAGCATAAGGAAAGGGGCCCGTCAAA GGATTGAAGTATATTCCGATTAAGG	D39	3’ flanking fragment
P1056	AAAGCTGAATGCCAATCCCACCACCTGCTT		
**For construction of K522 (Δ*cps* Δ*pnpR*::P_c-[*kan*^{R}-*rpsL*$^+$])**			
P1127	AGCGGCTTGAGTTTGCTGGATTTACGTGGA	D39	5’ flanking fragment
P1129	CATTATCCATTAAAAATCAAACGGATCCTA GTAGTCAAGCAATTTCAGAATGTGTTCTTC		
Kan rpsL forward	TAGGATCCGTTTGATTTTTAATGGATAATG	P_c-[*kan*^{R}-*rpsL*$^+$] cassette[c]	P_c-[*kan*^{R}-*rpsL*$^+$]
Kan rpsL reverse	GGGCCCCTTTCCTTATGCTTTTG		
P1130	CAAAAGCATAAGGAAAGGGGCCCAAGTTG AGGGAAAAAATTGAAGACAATCC	D39	3’ flanking fragment
P1128	ACATCATCTGGCACCTGCACCTTTAGCTGT		
**For construction of K577 (Δ*cps* Δ*pnpRS*::P_c-[*kan*^{R}-*rpsL*$^+$])**			
P1127	AGCGGCTTGAGTTTGCTGGATTTACGTGGA	D39	5’ flanking fragment
P1129	CATTATCCATTAAAAATCAAACGGATCCTA GTAGTCAAGCAATTTCAGAATGTGTTCTTC		

Kan rpsL forward	TAGGATCCGTTTGATTTTTAATGGATAATG	P_c-[*kan*^{R}-*rpsL*$^+$] cassette[c]	P_c-[*kan*^{R}-*rpsL*$^+$]
Kan rpsL reverse	GGGCCCCTTTCCTTATGCTTTTG		
P451	CAAAAGCATAAGGAAAGGGGCCCCTTGGC AGAGGCAGTTGCTTCAC	D39	3' flanking fragment
P449	GTGACCACTTGTTGACCTTGTTTGGAGTGG		
**For construction of K579 (Δ*cps* Δ*phoU2*::P_c-[*kan*^{R}-*rpsL*$^+$])**			
P798	ACCAGTTCTACGCTATGGGACAAGAAGTGC	D39	5' flanking fragment
P800	CATTATCCATTAAAAATCAAACGGATCCTA CTGAGAACCATTAGAACCTGACTGGCA		
Kan rpsL forward	TAGGATCCGTTTGATTTTTAATGGATAATG	P_c-[*kan*^{R}-*rpsL*$^+$] cassette[c]	P_c-[*kan*^{R}-*rpsL*$^+$]
Kan rpsL reverse	GGGCCCCTTTCCTTATGCTTTTG		
P801	CAAAAGCATAAGGAAAGGGGCCCGAAGGA ACTTACCAAAGCTATCTTGAAAAAG	D39	3' flanking fragment
P799	ACAAGCCAAGGAAATCTTGGTGGAACAGCC		
**For construction of K581 (Δ*cps* Δ*pstB2*[1]::P_c-[*kan*^{R}-*rpsL*$^+$])**			
P1261	GGGAATTGGGAACACTGTCATGGGAACTGT	D39	5' flanking fragment
P1263	CATTATCCATTAAAAATCAAACGGATCCTA GGCTACTTTTTCTTCAGGAAAGGTAAGG		
Kan rpsL forward	TAGGATCCGTTTGATTTTTAATGGATAATG	P_c-[*kan*^{R}-*rpsL*$^+$] cassette[c]	P_c-[*kan*^{R}-*rpsL*$^+$]
Kan rpsL reverse	GGGCCCCTTTCCTTATGCTTTTG		
P1264	CAAAAGCATAAGGAAAGGGGCCCATTTTCC AAAATGCCAAGCTACAGTC	D39	3' flanking fragment
P1262	TGGAACGGGTTACCAGAAGCATGGTGTACT		
**For construction of K583 (Δ*cps* Δ*pst2-phoU2*::P_c-[*kan*^{R}-*rpsL*$^+$])**			
P1055	ACAAGTTGCCTTGTGGCAGGAATTTGCCCA	D39	5' flanking fragment
P1057	CATTATCCATTAAAAATCAAACGGATCCTA TAAACAAGCCGTCAGCCAAAAAGC		
Kan rpsL forward	TAGGATCCGTTTGATTTTTAATGGATAATG	P_c-[*kan*^{R}-*rpsL*$^+$] cassette[c]	P_c-[*kan*^{R}-*rpsL*$^+$]
Kan rpsL reverse	GGGCCCCTTTCCTTATGCTTTTG		
P1058	CAAAAGCATAAGGAAAGGGGCCCGTCAAA GGATTGAAGTATATTCCGATTAAGG	D39	3' flanking fragment
P1056	AAAGCTGAATGCCAATCCCACCACCTGCTT		
**For construction of K591 (Δ*cps* Δ*phoU1*::P_c-[*kan*^{R}-*rpsL*$^+$])**			
P1265	ACGCCAAATAGTCTCATGTCTTCAGGCCGT	D39	5' flanking fragment
P1267	CATTATCCATTAAAAATCAAACGGATCCTA CCCTAGTCCTAAAAAGGATTGTTCTAATTC		
Kan rpsL forward	TAGGATCCGTTTGATTTTTAATGGATAATG	P_c-[*kan*^{R}-*rpsL*$^+$] cassette[c]	P_c-[*kan*^{R}-*rpsL*$^+$]
Kan rpsL reverse	GGGCCCCTTTCCTTATGCTTTTG		
P1268	CAAAAGCATAAGGAAAGGGGCCCGCTAAC	D39	3' flanking

	ATTTGTGAACGCCTAGTCTACC		fragment
P1266	GGAAGTTCTTGTAGGTTGCGAGAGTTTGGC GA		
**For construction of K593 (Δ*cps* Δ*pnpRS-pst1- phoU1*::P_c-[*kan*^{R}-*rpsL*$^+$])**			
P1127	AGCGGCTTGAGTTTGCTGGATTTACGTGGA	D39	5' flanking fragment
P1129	CATTATCCATTAAAAATCAAACGGATCCTA GTAGTCAAGCAATTTCAGAATGTGTTCTTC		
Kan rpsL forward	TAGGATCCGTTTGATTTTTAATGGATAATG	P_c-[*kan*^{R}-*rpsL*$^+$] cassette[c]	P_c-[*kan*^{R}-*rpsL*$^+$]
Kan rpsL reverse	GGGCCCCTTTCCTTATGCTTTTG		
P1268	CAAAAGCATAAGGAAAGGGGCCCGCTAAC ATTTGTGAACGCCTAGTCTACC	D39	3' flanking fragment
P1266	GGAAGTTCTTGTAGGTTGCGAGAGTTTGGC GA		
**For construction of K595 (Δ*cps* Δ*pst1- phoU1*::P_c-[*kan*^{R}-*rpsL*$^+$])**			
P567	ACAGCTAAAGGTGCAGGTGCCAGATGATGT	D39	5' flanking fragment
P569	CATTATCCATTAAAAATCAAACGGATCCTA AACAAGCCCAAATCCTGATAAGCCAA		
Kan rpsL forward	TAGGATCCGTTTGATTTTTAATGGATAATG	P_c-[*kan*^{R}-*rpsL*$^+$] cassette[c]	P_c-[*kan*^{R}-*rpsL*$^+$]
Kan rpsL reverse	GGGCCCCTTTCCTTATGCTTTTG		
P1406	CAAAAGCATAAGGAAAGGGGCCCACTGAA AAAGTTTTAATTCCTGACGATGTTC	D39	3' flanking fragment
P458	CCCATTTGGTGTAACTGTTCTTCGTCAGGG		
**For construction of K665 (Δ*cps* Δ*pstB1*::P_c-[*kan*^{R}-*rpsL*$^+$])**			
P1403	TCTGGAAATGGCTTACGCATCAGGTCAGCA	D39	5' flanking fragment
P1405	CATTATCCATTAAAAATCAAACGGATCCTA TTTTAAGGCTTGAAAATCCCCGTAA		
Kan rpsL forward	TAGGATCCGTTTGATTTTTAATGGATAATG	P_c-[*kan*^{R}-*rpsL*$^+$] cassette[c]	P_c-[*kan*^{R}-*rpsL*$^+$]
Kan rpsL reverse	GGGCCCCTTTCCTTATGCTTTTG		
P1406	CAAAAGCATAAGGAAAGGGGCCCGAAGAC TATATTTCAGGACGGTTCGGA	D39	3' flanking fragment
P1404	AGCCAGACAGCCCATCATCATACGTACTTC A		
**For construction of K667 (Δ*cps* Δ*pst1*::P_c-[*kan*^{R}-*rpsL*$^+$])**			
P567	ACAGCTAAAGGTGCAGGTGCCAGATGATGT	D39	5' flanking fragment
P569	CATTATCCATTAAAAATCAAACGGATCCTA AACAAGCCCAAATCCTGATAAGCCAA		
Kan rpsL forward	TAGGATCCGTTTGATTTTTAATGGATAATG	P_c-[*kan*^{R}-*rpsL*$^+$] cassette[c]	P_c-[*kan*^{R}-*rpsL*$^+$]
Kan rpsL reverse	GGGCCCCTTTCCTTATGCTTTTG		
P1406	CAAAAGCATAAGGAAAGGGGCCCGAAGAC TATATTTCAGGACGGTTCGGA	D39	3' flanking fragment
P1404	AGCCAGACAGCCCATCATCATACGTACTTC		

	A		
**For construction of K669 (Δ*cps* Δ*pstC1-pstB1*::P$_c$-[*kan*^{R}-*rpsL*$^+$])**			
P452	TCAGCTGCTTCCAAACAGTCAGCTTCAGGA	D39	5' flanking fragment
P454	CATTATCCATTAAAAATCAAACGGATCCTA TGTTGCACTCATGAAAAAAATTGCC		
Kan rpsL forward	TAGGATCCGTTTGATTTTTAATGGATAATG	P$_c$-[*kan*^{R}-*rpsL*$^+$] cassette[c]	P$_c$-[*kan*^{R}-*rpsL*$^+$]
Kan rpsL reverse	GGGCCCCTTTCCTTATGCTTTTG		
P1406	CAAAAGCATAAGGAAAGGGGCCCGAAGAC TATATTTCAGGACGGTTCGGA	D39	3' flanking fragment
P1404	AGCCAGACAGCCCATCATCATACGTACTTC A		
**For construction of K671 (Δ*cps* Δ*pstB2*^{2}::P$_c$-[*kan*^{R}-*rpsL*$^+$])**			
P1407	AACCATTGTATCAGCAGGTTCTGCCGCTGT	D39	5' flanking fragment
P1409	CATTATCCATTAAAAATCAAACGGATCCTA GTCTGATACCTGTAAAATCGCATCTGTCA		
Kan rpsL forward	TAGGATCCGTTTGATTTTTAATGGATAATG	P$_c$-[*kan*^{R}-*rpsL*$^+$] cassette[c]	P$_c$-[*kan*^{R}-*rpsL*$^+$]
Kan rpsL reverse	GGGCCCCTTTCCTTATGCTTTTG		
P1410	AACGTCCAAAAGCATAAGGAAAGGGGCCC AAGACTATATTACAGGAAAATTTGGATAA	D39	3' flanking fragment
P1408	ATGTCTGCACCGACTACCTGGTTCTTTCCA		
**For construction of K673 (Δ*cps* Δ*pst2*::P$_c$-[*kan*^{R}-*rpsL*$^+$])**			
P1055	ACAAGTTGCCTTGTGGCAGGAATTTGCCCA	D39	5' flanking fragment
P1057	CATTATCCATTAAAAATCAAACGGATCCTA TAAACAAGCCGTCAGCCAAAAAGC		
Kan rpsL forward	TAGGATCCGTTTGATTTTTAATGGATAATG	P$_c$-[*kan*^{R}-*rpsL*$^+$] cassette[c]	P$_c$-[*kan*^{R}-*rpsL*$^+$]
Kan rpsL reverse	GGGCCCCTTTCCTTATGCTTTTG		
P1410	AACGTCCAAAAGCATAAGGAAAGGGGCCC AAGACTATATTACAGGAAAATTTGGATAA	D39	3' flanking fragment
P1408	ATGTCTGCACCGACTACCTGGTTCTTTCCA		
**For construction of K675 (Δ*cps* ΔpstC2-*pstB2*^{2}::P$_c$-[*kan*^{R}-*rpsL*$^+$])**			
P497	TTGCAACCCTTGGTTGAAGTAGCGGCAGAT	D39	5' flanking fragment
P499	CATTATCCATTAAAAATCAAACGGATCCTA CTCCAGACGAGAATTCTTTGATGGAA		
Kan rpsL forward	TAGGATCCGTTTGATTTTTAATGGATAATG	P$_c$-[*kan*^{R}-*rpsL*$^+$] cassette[c]	P$_c$-[*kan*^{R}-*rpsL*$^+$]
Kan rpsL reverse	GGGCCCCTTTCCTTATGCTTTTG		
P1410	AACGTCCAAAAGCATAAGGAAAGGGGCCC AAGACTATATTACAGGAAAATTTGGATAA	D39	3' flanking fragment
P1408	ATGTCTGCACCGACTACCTGGTTCTTTCCA		
**For construction of K723 (Δ*cps* Δ*spd0443*::P$_c$-[*kan*^{R}-*rpsL*$^+$])**			
P1465	TGAACTTTTGCTTCCTTGCTAGCTTTTTCA	D39	5' flanking fragment
P1467	CATTATCCATTAAAAATCAAACGGATCCTA		

	TAAGAATAGCCCCAGACCACCTAAAAAGTG		
Kan rpsL forward	TAGGATCCGTTTGATTTTTAATGGATAATG	P_c-[*kan*^{R}-*rpsL*$^+$] cassette^c	P_c-[*kan*^{R}-*rpsL*$^+$]
Kan rpsL reverse	GGGCCCCTTTCCTTATGCTTTTG		
P1468	AAACGTCCAAAAGCATAAGGAAAGGGGCC CACTCGTGTATCAGACCACGCTATGAACCT T	D39	3' flanking fragment
P1466	TGCCTTTGTTACTGCAGCGGTTGAGAACAG		
**For construction of E577 (Δ*cps* Δ*pnpRS*::P_c-[*Erm*^{R}])**			
P1127	AGCGGCTTGAGTTTGCTGGATTTACGTGGA	D39	5' flanking fragment
P1129	CATTATCCATTAAAAATCAAACGGATCCTA GTAGTCAAGCAATTTCAGAATGTGTTCTTC		
Erm forward	TAGGATCCGTTTGATTTTTAATGGATAATG	P_c-[Erm^R] cassette^c	P_c-[*Erm*^{R}]
Erm reverse	GGGCCCCTTTCCTTATGCTTTTG		
P451	CAAAAGCATAAGGAAAGGGGCCCCTTGGC AGAGGCAGTTGCTTCAC	D39	3' flanking fragment
P449	GTGACCACTTGTTGACCTTGTTTGGAGTGG		
**For construction of E579 (Δ*cps* Δ*phoU2*::P_c-[Erm^R])**			
P798	ACCAGTTCTACGCTATGGGACAAGAAGTGC	D39	5' flanking fragment
P800	CATTATCCATTAAAAATCAAACGGATCCTA CTGAGAACCATTAGAACCTGACTGGCA		
Erm forward	TAGGATCCGTTTGATTTTTAATGGATAATG	P_c-[Erm^R] cassette^c	P_c-[*Erm*^{R}]
Erm reverse	GGGCCCCTTTCCTTATGCTTTTG		
P801	CAAAAGCATAAGGAAAGGGGCCCGAAGGA ACTTACCAAAGCTATCTTGAAAAAG	D39	3' flanking fragment
P799	ACAAGCCAAGGAAATCTTGGTGGAACAGCC		
**For construction of E583 (Δ*cps* Δ*pst2-phoU2*::P_c-[*Erm*^{R}])**			
P1055	ACAAGTTGCCTTGTGGCAGGAATTTGCCCA	D39	5' flanking fragment
P1057	CATTATCCATTAAAAATCAAACGGATCCTA TAAACAAGCCGTCAGCCAAAAAGC		
Erm forward	TAGGATCCGTTTGATTTTTAATGGATAATG	P_c-[Erm^R] cassette^c	P_c-[*Erm*^{R}]
Erm reverse	GGGCCCCTTTCCTTATGCTTTTG		
P1058	CAAAAGCATAAGGAAAGGGGCCCGTCAAA GGATTGAAGTATATTCCGATTAAGG	D39	3' flanking fragment
P1056	AAAGCTGAATGCCAATCCCACCACCTGCTT		
**For construction of E591 (Δ*cps* Δ*phoU1*::P_c-[*Erm*^{R}])**			
P1265	ACGCCAAATAGTCTCATGTCTTCAGGCCGT	D39	5' flanking fragment
P1267	CATTATCCATTAAAAATCAAACGGATCCTA CCCTAGTCCTAAAAAGGATTGTTCTAATTC		
Erm forward	TAGGATCCGTTTGATTTTTAATGGATAATG	P_c-[Erm^R] cassette^c	P_c-[*Erm*^{R}]
Erm	GGGCCCCTTTCCTTATGCTTTTG		

reverse			
P1268	CAAAAGCATAAGGAAAGGGGCCCGCTAAC ATTTGTGAACGCCTAGTCTACC	D39	3’ flanking fragment
P1266	GGAAGTTCTTGTAGGTTGCGAGAGTTTGGC GA		
**For construction of E593 (Δ*cps* Δ*pnpRS-pst1-phoU1*::P$_c$-[*Erm*^{R}])**			
P1127	AGCGGCTTGAGTTTGCTGGATTTACGTGGA	D39	5’ flanking fragment
P1129	CATTATCCATTAAAAATCAAACGGATCCTA GTAGTCAAGCAATTTCAGAATGTGTTCTTC		
Erm forward	TAGGATCCGTTTGATTTTTAATGGATAATG	P$_c$-[Erm^R] cassette[c]	P$_c$-[*Erm*^{R}]
Erm reverse	GGGCCCCTTTCCTTATGCTTTTG		
P1268	CAAAAGCATAAGGAAAGGGGCCCGCTAAC ATTTGTGAACGCCTAGTCTACC	D39	3’ flanking fragment
P1266	GGAAGTTCTTGTAGGTTGCGAGAGTTTGGC GA		
**For construction of E595 (Δ*cps* Δ*pst1-phoU1*::P$_c$-[Erm^R])**			
P567	ACAGCTAAAGGTGCAGGTGCCAGATGATGT	D39	5’ flanking fragment
P569	CATTATCCATTAAAAATCAAACGGATCCTA AACAAGCCCAAATCCTGATAAGCCAA		
Erm forward	TAGGATCCGTTTGATTTTTAATGGATAATG	P$_c$-[Erm^R] cassette[c]	P$_c$-[*Erm*^{R}]
Erm reverse	GGGCCCCTTTCCTTATGCTTTTG		
P1406	CAAAAGCATAAGGAAAGGGGCCCACTGAA AAAGTTTTAATTCCTGACGATGTTC	D39	3’ flanking fragment
P458	CCCATTTGGTGTAACTGTTCTTCGTCAGGG		
**For construction of E665 (Δ*cps* Δ*pstB1*::P$_c$-[*Erm*^{R}])**			
P1403	TCTGGAAATGGCTTACGCATCAGGTCAGCA	D39	5’ flanking fragment
P1405	CATTATCCATTAAAAATCAAACGGATCCTA TTTTAAGGCTTGAAAATCCCCGTAA		
Erm forward	TAGGATCCGTTTGATTTTTAATGGATAATG	P$_c$-[*Erm*^{R}] cassette[c]	P$_c$-[*Erm*^{R}]
Erm reverse	GGGCCCCTTTCCTTATGCTTTTG		
P1406	CAAAAGCATAAGGAAAGGGGCCCGAAGAC TATATTTCAGGACGGTTCGGA	D39	3’ flanking fragment
P1404	AGCCAGACAGCCCATCATCATACGTACTTC A		
**For construction of E667 (Δ*cps* Δ*pst1*::P$_c$-[Erm^R-])**			
P567	ACAGCTAAAGGTGCAGGTGCCAGATGATGT	D39	5’ flanking fragment
P569	CATTATCCATTAAAAATCAAACGGATCCTA AACAAGCCCAAATCCTGATAAGCCAA		
Erm forward	TAGGATCCGTTTGATTTTTAATGGATAATG	P$_c$-[*Erm*^{R}] cassette[c]	P$_c$-[*Erm*^{R}]
Erm reverse	GGGCCCCTTTCCTTATGCTTTTG		
P1406	CAAAAGCATAAGGAAAGGGGCCCGAAGAC	D39	3’ flanking

	TATATTTCAGGACGGTTCGGA		fragment
P1404	AGCCAGACAGCCCATCATCATACGTACTTCA		
**For construction of E669 (Δ*cps* Δ*pstC1-pstB1*::P_c-[*Erm*^{R}])**			
P452	TCAGCTGCTTCCAAACAGTCAGCTTCAGGA	D39	5’ flanking fragment
P454	CATTATCCATTAAAAATCAAACGGATCCTATGTTGCACTCATGAAAAAAATTGCC		
Erm forward	TAGGATCCGTTTGATTTTTAATGGATAATG	P_c-[*Erm*^{R}] cassette[c]	P_c-[*Erm*^{R}]
Erm reverse	GGGCCCCTTTCCTTATGCTTTTG		
P1406	CAAAAGCATAAGGAAAGGGGCCCGAAGACTATATTTCAGGACGGTTCGGA	D39	3’ flanking fragment
P1404	AGCCAGACAGCCCATCATCATACGTACTTCA		
**For construction of E671 (Δ*cps* Δ*pstB2*^{2}::P_c-[*Erm*^{R}])**			
P1407	AACCATTGTATCAGCAGGTTCTGCCGCTGT	D39	5’ flanking fragment
P1409	CATTATCCATTAAAAATCAAACGGATCCTAGTCTGATACCTGTAAAATCGCATCTGTCA		
Erm forward	TAGGATCCGTTTGATTTTTAATGGATAATG	P_c-[*Erm*^{R}] cassette[c]	P_c-[*Erm*^{R}]
Erm reverse	GGGCCCCTTTCCTTATGCTTTTG		
P1410	AACGTCCAAAAGCATAAGGAAAGGGGCCCAAGACTATATTACAGGAAAATTTGGATAA	D39	3’ flanking fragment
P1408	ATGTCTGCACCGACTACCTGGTTCTTTCCA		
**For construction of E673 (Δ*cps* Δ*pst2*::P_c-[Erm^R])**			
P1055	ACAAGTTGCCTTGTGGCAGGAATTTGCCCA	D39	5’ flanking fragment
P1057	CATTATCCATTAAAAATCAAACGGATCCTATAAACAAGCCGTCAGCCAAAAAGC		
Erm forward	TAGGATCCGTTTGATTTTTAATGGATAATG	P_c-[*Erm*^{R}] cassette[c]	P_c-[*Erm*^{R}]
Erm reverse	GGGCCCCTTTCCTTATGCTTTTG		
P1410	AACGTCCAAAAGCATAAGGAAAGGGGCCCAAGACTATATTACAGGAAAATTTGGATAA	D39	3’ flanking fragment
P1408	ATGTCTGCACCGACTACCTGGTTCTTTCCA		
**For construction of E675 (Δ*cps* Δ*pstC2-pstB2*^{2}::P_c-[*Erm*^{R}])**			
P497	TTGCAACCCTTGGTTGAAGTAGCGGCAGAT	D39	5’ flanking fragment
P499	CATTATCCATTAAAAATCAAACGGATCCTACTCCAGACGAGAATTCTTTGATGGAA		
Erm forward	TAGGATCCGTTTGATTTTTAATGGATAATG	P_c-[*Erm*^{R}] cassette[c]	P_c-[*Erm*^{R}]
Erm reverse	GGGCCCCTTTCCTTATGCTTTTG		
P1410	AACGTCCAAAAGCATAAGGAAAGGGGCCCAAGACTATATTACAGGAAAATTTGGATAA	D39	3’ flanking fragment
P1408	ATGTCTGCACCGACTACCTGGTTCTTTCCA		
**For construction of E723 (Δ*cps* Δ*spd0443*::P_c-[*Erm*^{R}])**			

P1465	TGAACTTTTGCTTCCTTGCTAGCTTTTTCA	D39	5' flanking fragment
P1467	CATTATCCATTAAAAATCAAACGGATCCTA TAAGAATAGCCCCAGACCACCTAAAAAGTG		
Erm forward	TAGGATCCGTTTGATTTTTAATGGATAATG	P_c-[*Erm*^{R}] cassette[c]	P_c-[*Erm*^{R}]
Erm reverse	GGGCCCCTTTCCTTATGCTTTTG		
P1468	AAACGTCCAAAAGCATAAGGAAAGGGGCC CACTCGTGTATCAGACCACGCTATGAACCT T	D39	3' flanking fragment
P1466	TGCCTTTGTTACTGCAGCGGTTGAGAACAG		
**For construction of IU6356 (*pnpR-L-FLAG*^{3}-P_c-[*Erm*^{R}])**			
P448	ACTACGAAGCAGTCACTTTAGGCCTGGACA	D39	5' flanking fragment
JQ17	GCGGAGCCAGCGGAACCTAACTCCTTGAAC TTATAACCATAACCCCG		
JQ19	GGTTATGGTTATAAGTTCAAGGAGTTAGGT TCCGCTGGCTCCGCT	IU4052	L-F^3-Pcerm
JQ20	AAATTGAAGGTAGCGTTTCATTTATTTCCTC CCGTTAAATAATAGATAACTATTAAAAAT		
JQ18	AGTTATCTATTATTTAACGGGAGGAAATAA ATGAAACGCTACCTTCAATTTTGGC	D39	3' flanking fragment
P1128	ACATCATCTGGCACCTGCACCTTTAGCTGT		
**For construction of IU6377 (Δ*phoU1*)**			
P1265	ACGCCAAATAGTCTCATGTCTTCAGGCCGT	D39	5' flanking fragment
JQ11	ACTAGGCGTTCACAAATGTTAGCCCCTAGT CCTAAAAAGGATTGTTCTAATTC		
JQ12	GAACAATCCTTTTTAGGACTAGGGGCTAAC ATTTGTGAACGCCTAGTCTACC	D39	3' flanking fragment
P1266	GGAAGTTCTTGTAGGTTGCGAGAGTTTGGC GA		
**For construction of IU6379 (Δ*pnpR*)**			
P1127	AGCGGCTTGAGTTTGCTGGATTTACGTGGA	D39	5' flanking fragment
JQ13	GTCTTCAATTTTTTCCCTCAACTTGTAGTCA AGCAATTTCAGAATGTGTTCTTC		
JQ14	ACACATTCTGAAATTGCTTGACTACAAGTT GAGGGAAAAAATTGAAGACAATC	D39	3' flanking fragment
P1128	ACATCATCTGGCACCTGCACCTTTAGCTGT		
**For construction of IU6381 (Δ*pnpRS*)**			
P1127	AGCGGCTTGAGTTTGCTGGATTTACGTGGA	D39	5' flanking fragment
JQ15	GCAACTGCCTCTGCCAAGGTAGTCAAGCAA TTTCAGAATGTGTTCTTC		
JQ16	ACACATTCTGAAATTGCTTGACTACCTTGGC AGAGGCAGTTGCTTCA	D39	3' flanking fragment
P1128	ACATCATCTGGCACCTGCACCTTTAGCTGT		
**For construction of IU6397 (Δ*phoU2 bgaA*::*kan-T1T2-*P_{ftsA}*-phoU2*)**			
P146	TGGCCATTCATCGCTGGTCGTGCTGAAAT	D39	5' flanking fragment
JQ23	TCTTCTTCAAATTGAGATCGTAACATTACAT CGCTTCCTCTCTATCTTCCAAGT		

JQ25	GGAAGATAGAGAGGAAGCGATGTAATGTT ACGATCTCAATTTGAAGAAGATTTAGAG	IU5295	bga::kan-T1T2-PftsA-phoU2 cassette
JQ26	AACTGGTTTATGAGAAAGTAAGTTCTTTTAT AGTTCGACAATCTTACCTGTTTCAAAGTA		
JQ24	GAAACAGGTAAGATTGTCGAACTATAAAAG AACTTACTTTCTCATAAACCAGTTGCTG	D39	3' flanking fragment
CS121	GCTTTCTTGAGGCAATTCACTTGGTGC		
**For construction of IU6496 (Δ*pnpS*)**			
P448	AGTCCCTGCAATGGTCAAAGCAACGGGTAA	D39	5' flanking fragment
JQ47	TGAAGCAACTGCCTCTGCCAAGTGCAATCA GAATGAGGCTAACACTTAGATT		
JQ48	AAGTGTTAGCCTCATTCTGATTGCACTTGGC AGAGGCAGTTGCTTCACGATTTTT	D39	3' flanking fragment
P449	AGGTCGCCTACCTTGACTTGTTCCAAAGGA		
**For construction of IU6550 (Δ*pst2-phoU2*)**			
P1055	ACAAGTTGCCTTGTGGCAGGAATTTGCCCA	D39	5' flanking fragment
JQ49	CAACCCATTCACAGATATTTTTAGCTAAAC AAGCCGTCAGCCAAAAAGCGATAAG		
JQ50	TTTTGGCTGACGGCTTGTTTAGCTAAAAATA TCTGTGAATGGGTTGTCTAC	D39	3' flanking fragment
P1258	CAGCCTGCAATTCATTGACTGCTTCACCCA		
**For construction of IU6610 (Δ*pst2*)**			
P1055	ACAAGTTGCCTTGTGGCAGGAATTTGCCCA	D39	5' flanking fragment
JQ55	CGTTTCCTTGTGTTGGGGATCAAGGAACAT TAAACAAGCCGTCAGCCAAAAAGCGATAA G		
JQ56	CTTATCGCTTTTTGGCTGACGGCTTGTTTAA TGTTCCTTGATCCCCAACACAAGGAAACG	D39	3' flanking fragment
P1408	ATGTCTGCACCGACTACCTGGTTCTTTCCA		
**For construction of IU6638 (Δ*pst1*)**			
P567	ACAGCTAAAGGTGCAGGTGCCAGATGATGT	D39	5' flanking fragment
JQ57	TTATCCGAACCGTCCTGAAATATAGTCTTCA ACAAGCCCAAATCCTGATAAGCCAATGGC		
JQ58	GCCATTGGCTTATCAGGATTTGGGCTTGTTG AAGACTATATTTCAGGACGGTTCGGATAA	D39	3' flanking fragment
P458	CCCATTTGGTGTAACTGTTCTTCGTCAGGG		
**For construction of IU6689 (*pnpR*-L-FLAG^3)**			
P448	ACTACGAAGCAGTCACTTTAGGCCTGGACA	D39	5' flanking fragment
JQ65	AAAATTGAAGGTAGCGTTTCATTTATTTATC ATCATCATCTTTATAATCTTTATCATCAT		
JQ66	AAAGATTATAAAGATGATGATGATAAATAA ATGAAACGCTACCTTCAATTTTGGCTAGTC	D39	3' flanking fragment
P1128	ACATCATCTGGCACCTGCACCTTTAGCTGT		
**For construction of IU6830 (Δ*pst1-phoU1*)**			
P567	ACAGCTAAAGGTGCAGGTGCCAGATGATGT	D39	5' flanking fragment
JQ69	TAGGTAGACTAGGCGTTCACAAATGTTAGC AACAAGCCCAAATCCTGATAAGCCAATGGC		

JQ70	GCCATTGGCTTATCAGGATTTGGGCTTGTTG CTAACATTTGTGAACGCCTAGTCTACCTA	D39	3’ flanking fragment
P1266	GGAAGTTCTTGTAGGTTGCGAGAGTTTGGC GA		
**For construction of IU6830 (Δ*spd0443*)**			
P1465	TGAACTTTTGCTTCCTTGCTAGCTTTTTCA	D39	5’ flanking fragment
JQ79	AAGGTTCATAGCGTGGTCTGATACACGAGT TAAGAATAGCCCCAGACCACCTAAAAAGTG		
JQ80	CACTTTTTAGGTGGTCTGGGGCTATTCTTAA CTCGTGTATCAGACCACGCTATGAACCTT	D39	3’ flanking fragment
P1466	TGCCTTTGTTACTGCAGCGGTTGAGAACAG		

[a]Genomic DNA of indicated *S. pneumoniae* strains was used as templates for PCR reactions, except for P_c-[*kan*^{R}-*rpsL*$^+$] cassettes. aa = amino acids.

[b]P_c-[*kan*^{R}-*rpsL*$^+$] cassette PCR product was generated using primers Kan rpsL forward and Kan rpsL reverse, and a lysate from strain IU1885 (Wayne *et al*., 2010).

Table S3. Oligonucleotide primers used for qRT-PCR

Prime	Sequence (5' to 3')	Gene name
KW096	CCAAACAGTCAGCTTCAGGAACGA	*pstS1*
KW097	AGATCCCAAGGAGATGTAGCCGAT	
JQ33	TAGCTGGCTTGGCTCTGATTGTCA	*pstS2*
JQ34	TGCATGGCAGACTGACCTTCCATA	
JQ39	GGAAGGCCTTAGCTTTGGCAGAAA	*pnpR*
JQ40	TTGACACGCGCCAGCAATTCTCTA	
KK387	CAGCAGTAGGGAATCTTCGGCAAT	16s rRNA
KK388	TACGCCCAATAAATCCGGACAACG	
KK489	AAAGGTCGTGGTGGTAAGGGAATG	*gyrA*
KK490	GCATCTTGATCCAGGCGCATTACT	

Table S4. Growth yields and doubling times of encapsulated parent strain and mutants grown in BHI broth (≈18 mM P_i)

Strain[a]	Growth yield (maximum OD_{620})[b]	Doubling time (min^{-1})[c]
Parent strain (IU1781)	0.99 ± 0.01 (n=11)	44.1 ± 1.4 (n=11) (ns)
Δ*phoU2* (IU6375)	0.72 ± 0.03 (n=11) (***)	46.0 ± 1.9 (n=11) (ns)
Δ*phoU2* // *phoU2*$^+$(IU6397)	0.92 ± 0.02 (n=3) (ns)	44.6 ± 2.4 (n=3) (ns)
Δ*phoU1* (IU6377)	1.04 ± 0.01 (n=3) (ns)	38.9 ± 0.7 (n=3) (ns)
Δ*phoU2* Δ*phoU1* (IU6499)	0.56 ± 0.02 (n=8) (***)	55.1 ± 3.6 (n=8) (**)
Δ*pst2-phoU2* (IU6550)	0.97 ± 0.01 (n=5) (ns)	42.2 ± 2.0 (n=5) (ns)
Δ*pst2* (IU6610)	0.98 ± 0.01 (n=2) (ns)	48.1 ± 0.2 (n=2) (ns)
Δ*pnpRS* (IU6381)	0.92 (n=1)	49.7 (n=1)
Δ*pst1* (IU6638)	0.98 ± 0.04 (n=2) (ns)	43.7 ± 3.2 (n=2) (ns)
Δ*pst1-phoU1* (IU6830)	1.02 ± 0.01 (n=2) (ns)	41.1 ± 0.1 (n=2) (ns)
Δ*phoU2* Δ*pst1* (IU6664)	0.83 ± 0.03 (n=3) (***)	42.7 ± 5.5 (n=3) (ns)
Δ*phoU2* Δ*pst1-phoU1* (IU6854)	0.73 ± 0.02 (n=3) (***)	42.4 ± 1.6 (n=3) (ns)
Δ*pst2-phoU2* Δ*phoU1* (IU6612)	0.77 ± 0.01 (n=6) (***)	42.9 ± 2.4 (n=6) (ns)

[a]Strains contained markerless deletions in encapsulated parent strain (IU1781).

[b]The highest OD_{620} reached during stationary phase was used as the growth yield. P-values were determined by unpaired t tests compared to the parent strain (IU1781). ***, P<0.001; ns: not significant. The P-value of the Δ*pnpRS* mutant is not available, because n=1.

[c]Doubling times were determined for growth during the exponential phase. P-values were determined by unpaired t tests compared to the parent strain (IU1781). **, P<0.01.

Table S5. A Δ*phoU2* mutant is more sensitive than the parent strain to different classes of antibiotics[a]

Class	Antibiotic[b]	Inhibition zone diameter (mm)		P value[c]
		WT (IU1781)	Δ*phoU*2 (IU6375)	
β-lactam	cefotaxime	39.2 ± 0.7 (n=4)	49.8 ± 1.6 (n=4)	***
	cefazolin	32.3 ± 1.6 (n=4)	40.0 ± 1.1 (n=4)	**
	cefamandole	28.8 ± 0.9 (n=4)	36.3 ± 0.5 (n=4)	***
	ceftazidime	29.5 ± 1.6 (n=4)	38.8 ± 0.6 (n=4)	**
	amdinocillin	18.8 ± 1.0 (n=4)	27.5 ± 0.9 (n=4)	***
Glycopeptides inhibitor	vancomycin	19.5 ± 0.6 (n=4)	24.8 ± 0.3 (n=4)	***
Protein synthesis inhibitor	gentamicin	15.3 ± 1.3 (n=4)	19.5 ± 1.0 (n=4)	*
	tetracycline	26.0 ± 0.9 (n=4)	31.0 ± 0.4 (n=4)	**

[a]Disk diffusion assays were performed as described in *Materials and Methods*.

[b]Antibiotic disks were at the following concentrations: cefotaxime (30 μg); cefazolin (30 μg); cefamandole (30 μg); ceftazidime (30 μg); amdinocillin (10 μg); vancomycin (30 μg); gentamicin (120 μg); tetracycline (30 μg).

[c]*, P<0.05; **, P<0.01; ***, P<0.001. P-values were calculated by unpaired t test in GraphPad Prism compared to the parent strain for each antibiotic.

Table S6. RNA-Seq results for Δ*phoU2::kanrpsL*$^{+}$ single and Δ*phoU2::kanrpsL*$^{+}$ Δ*phoU1::*Pc-*erm* double mutants[a]

Δ*phoU2::kanrpsL*$^{+}$ mutant relative to a wild-type *CEP::kanrpsL*$^{+}$ strain (IU 3116)

SPD Number	Gene name	Gene description	Fold change	P value
**Increased relative transcript amounts in Δ*phoU2::kanrpsL*$^{+}$ mutant**				
0373	-	conserved hypothetical protein	+3.6	8.097E-08
0540	-	putative amino acid ABC transporter, amino acid-binding protein	+2.9	4.812E-09
1898	-	hypothetical protein	+3.1	1.559E-02
1899	-	glutamine amidotransferase	+3.3	5.079E-06
**1910**	***pstS1***	**phosphate ABC transporter, phosphate-binding protein**	**+17.5**	**5.296E-30**
**1911**	***pstC1***	**phosphate ABC transporter, membrane channel protein**	**+19.2**	**1.583E-21**
**1912**	***pstA1***	**phosphate ABC transporter, membrane channel protein**	**+19.6**	**1.608E-17**
**1913**	***pstB1***	**phosphate ABC transporter, ATPase**	**+21.7**	**3.529E-19**
**1914**	***phoU1***	**phosphate transport system regulatory protein PhoU**	**+23.8**	**1.437E-31**
2037	*cysK*	cysteine synthase	+2.3	2.508E-03
**Decreased relative transcript amounts in Δ*phoU2::kanrpsL*$^{+}$mutant**				
0104	-	LysM domain protein (WalRK regulon)	-2.9	6.484E-03
0447	-	transcriptional regulator, MerR family	-2.0	4.945E-02
0451		putative type I restriction-modification system, S subunit	-4.0	1.972E-07
0745	-	conserved hypothetical protein	-1.8	4.589E-02
**1227**	***phoU2***	**phosphate transport system regulatory protein PhoU** (deleted in mutant)	**-30.6**	**3.636E-55**
1488	-	ROK family protein	-2.1	3.284E-02
1801	-	ABC transporter, ATP-binding protein	-2.5	1.207E-02
1802	-	hypothetical protein	-2.1	1.754E-02
1874	-	LysM domain protein (WalRK regulon)	-4.1	1.277E-03
**2011**	***glpF***	**glycerol uptake facilitator protein**	**-3.6**	**2.429E-03**

**2012**	***glpO***	**hypothetical protein**	**-4.7**	**7.479E-08**
**2013**	***glpK***	**glycerol kinase**	**-5.3**	**1.222E-07**

Δ*phoU2::kanrpsL*⁺ Δ*phoU1::*Pc-*erm* mutant relative to a wild-type *CEP::kanrpsL*⁺ strain (IU 3116)

SPD Number	Gene name	Description	Fold change	P value
**Increased relative transcript amounts in Δ*phoU2::kanrpsL*⁺ Δ*phoU1::Pcerm* mutant**				
0112	-	conserved hypothetical protein	+1.8	4.454E-02
0167	*ribB*	3,4-dihydroxy-2-butanone 4-phosphate synthase/GTP cyclohydrolase II	+2.3	8.320E-03
0373	-	conserved hypothetical protein	+4.6	2.195E-10
0458	*hrcA*	heat-inducible transcription repressor	+1.9	4.456E-02
0461	*dnaJ*	chaperone protein	+2.0	3.131E-02
0540	-	putative amino acid ABC transporter, amino acid-binding protein	+2.8	8.627E-08
1898	-	hypothetical protein	+3.1	2.798E-02
1899	-	glutamine amidotransferase, class-I	+3.7	1.088E-06
**1910**	***pstS1***	**phosphate ABC transporter, phosphate-binding protein**	**+23.3**	**2.579E-35**
**1911**	***pstC1***	**phosphate ABC transporter, membrane channel protein**	**+27.0**	**6.988E-26**
**1912**	***pstA1***	**phosphate ABC transporter, membrane channel protein**	**+28.6**	**1.525E-21**
**1913**	***pstB1***	**phosphate ABC transporter, ATPase**	**+27.0**	**1.684E-21**
**1914**	***phoU1***	**phosphate transport system regulatory protein PhoU** (partially deleted in mutant)	**+6.0**	**1.227E-09**
1916	-	transcriptional regulator	+2.7	2.126E-03
1917	-	hypothetical protein	+5.5	3.451E-06
1965	*pcpA*	choline binding protein	+2.0	3.113E-02
2037	*cysK*	cysteine synthase	+2.2	2.660E-02
Decreased relative transcript amounts in ΔphoU2::kanrpsL⁺ ΔphoU1::Pcerm mutant				
0104	-	LysM domain protein	-2.9	4.237E-03

0169	*ribD*	riboflavin biosynthesis protein RibD	-2.0	4.514E-02
0447	-	transcriptional regulator, MerR family	-2.4	1.956E-03
0448	*glnA*	glutamine synthetase, type I	-2.3	1.600E-02
0449	-	hypothetical protein	-2.8	1.177E-02
0451	-	putative type I restriction-modification system, S subunit	-5.0	2.984E-11
1086	*mutY*	A/G-specific adenine glycosylase	-1.8	4.716E-02
1098	-	amino acid ABC transporter, amino acid-binding protein/permease protein	-2.4	5.124E-03
1099	-	amino acid ABC transporter, ATP-binding protein	-2.1	1.043E-02
1165	-	conserved hypothetical protein	-2.4	2.697E-02
1166	-	conserved hypothetical protein	-2.6	9.809E-03
1167	-	ABC transporter, ATP-binding protein	-2.3	4.297E-02
**1227**	***phoU2***	**putative phosphate transport system regulatory protein PhoU** (deleted in mutant)	**-36.6**	**1.692E-61**
1291	-	conserved hypothetical protein	-1.9	1.426E-02
1292	*ogt*	methylated-DNA--protein-cysteine S-methyltransferase	-1.9	3.079E-02
1360	-	hypothetical protein	-2.0	1.129E-02
1488	-	ROK family protein	-2.2	5.983E-03
1489	-	putative N-acetylneuraminate lyase	-2.4	3.606E-03
1490	-	hypothetical protein	-2.1	1.841E-03
1491	-	hypothetical protein	-2.0	4.295E-02
1492	-	conserved hypothetical protein	-2.2	5.694E-03
1493	-	sugar ABC transporter, permease protein	-2.2	4.777E-03
1494	-	sugar ABC transporter, permease protein	-2.5	1.525E-03
1587	-	putative transcriptional activator	-2.1	5.534E-03
1801	-	ABC transporter, ATP-binding protein	-2.5	7.764E-03

1802	-	hypothetical protein	-2.5	6.201E-04
1803	-	hypothetical protein	-2.3	4.456E-02
1874	-	LysM domain protein	-3.8	1.728E-03
**2011**	***glpF***	**glycerol uptake facilitator protein**	**-4.1**	**2.239E-04**
**2012**	***glpO***	**hypothetical protein**	**-5.3**	**1.980E-08**
**2013**	***glpK***	**glycerol kinase**	**-7.3**	**2.694E-10**
2014	-	conserved domain protein	-2.1	1.603E-10
2068	-	serine protease	-2.9	3.785E-02
2069	-	spoJ protein	-3.2	2.497E-02

[a]RNA preparation and RNA-Seq analyses were performed as described in *Materials and Methods*. RNA was prepared from cultures of strains IU6139 (Δ*phoU2*::*kanrpsL*$^+$) and IU6173 (Δ*phoU2*::*kanrpsL*$^+$ Δ*phoU1*::Pc-*erm*) grown exponentially in BHI media to OD_{620} to ≈0.15-0.2. Fold changes and false discovery rates (FDR) are based on three independent biological replicates. Cut-off limits were 1.8-fold and an FDR value<0.05. Genes belonging to the *pst1*, *pst2*, and *glpK* operons are shown in bold. RNA-Seq data were deposited in the NCBI GEO database under accession number GSE80637.

Table S7. Initial rates of P_i uptake in mCDM containing a moderately high P_i concentration (1 mM)[a]

Strain	Initial rate of P_i uptake (nmol/10^7 cells/min)[b]	P-value[c]
Encapsulated strain background		
Parent strain	1.3 ± 0.1 (n=3)	-
Δ*pst1-phoU1*	1.3 ± 0.1 (n=3)	ns
Δ*pst2-phoU2*	1.4 ± 0.1 (n=3)	ns
Unencapsulated strain background		
Parent strain Δ*cps*	1.8 ± 0.1 (n=3)	-
Δ*pst1-phoU1* Δ*cps*	1.8 ± 0.0 (n=3)	ns
Δ*pst2-phoU2* Δ*cps*	2.0 ± 0.1 (n=3)	ns
Δ*pst1-phoU1* Δ*pst2-phoU2* Δ*cps*	0.8 ± 0.0 (n=3)	***

[a]P_i uptake rates in high P_i condition were calculated based on the results from Fig. 8A and 8B.

[b]P_i uptake showed linear rates for 10 min (Fig. 8A and B). Rates of P_i uptake were calculated by the equation $P_{10}/10$, where P_{10} = P_i amount in cells at the 10 min.

[c]***, P<0.001; ns, not significant. P-values were calculated by unpaired t tests in GraphPad Prism compared to parent strains.

Table S8. Initial rates of P_i uptake in the first minute in low P_i condition (200 μM) following 1 h of P_i starvation[a]

Strain	Initial rate in first min (nmol/10^7 cells/min)[b]	P value[c]
Parent strain	10.2 ± 0.3 (n=3)	-
Δ*pst1-phoU1*	4.9 ± 0.2 (n=3)	***
Δ*pst2-phoU2*	8.4 ± 0.5 (n=3)	*

[a]P_i uptake rates in low P_i condition were calculated based on the result from Fig. 8C. Bacteria were starved for P_i in mCDM for 1 h before $^{32}P_i$ was added to a final concentration of 200 μM as described in *Materials and Methods*.

[b]The P_i uptake curves were not linear beyond 1 min and decreased with time. Initial rates were calculated for P_i uptake within the first minute after adding $^{32}P_i$.

[c]*, P<0.05; ***, P<0.001. P-values were calculated by an unpaired t test in GraphPad Prism compared to the parent strain.

SUPPLEMENTAL FIGURE LEGENDS

Fig. S1. Deletion of *pnpRS*, *pst1*, *pst2*, or *phoU1* has no effect on growth and β-lactam antibiotic sensitivity. (A) Representative growth curves of parent strain (IU1781) and Δ*phoU1* (IU6377), Δ*pnpRS* (IU6381), Δ*pst2* (IU6610), and Δ*pst1* (IU6638) mutants in BHI broth, which contains a high concentration of P_i (≈18 mM). (B) Cefotaxime sensitivity assays for encapsulated parent strain (IU1781) and Δ*phoU1* (IU6377), Δ*pnpRS* (IU6381), Δ*pst2* (IU6610), and Δ*pst1* (IU6638) mutants. See Fig. 2, *Materials and Methods*, and the text for additional details.

Fig. S2. RNA-Seq analysis identifies *pnpRS*, *pst1* and *pst2* as three distinct operons in *S. pneumoniae* D39. RNA samples (in triplicate) were prepared from wild-type D39 strain IU1781 for stranded RNA-Seq analysis that was run on the Illumina HiSeq2000 platform. The raw sequencing reads were adapter trimmed and mapped to the *S. pneumoniae* D39 (RefSeq NC_008533) genome. The three sets of tracks in blue represent the mapped reads from the three replicates of wild-type strain D39. Each set of tracks is comprised of reads corresponding to the "plus" (i.e., top) strand and the "minus" (i.e., bottom) strand; thus reflecting the direction of gene transcription. The difference in the mean expression values between two or more consecutive genes on the same strand was used to determine whether or not they comprised the same or different operons. Figure S2A shows that *pnpRS* (consisting of genes *spd_1908* (*pnpR*) and *spd_1909* (*pnpS*)) and the *pst1* locus (consisting of genes *pstS1*, *pstC1*, *pstA1*, *pstB1* and *phoU1*; *spd_2010* to *spd_2014*) are in two separate operons. Figure S2B similarly identifies the *pst2* locus (consisting of genes *pstS2, pstC2, pstA2,* $pstB2^1$, $pstB2^2$, and *phoU2*; *spd_1232* to *spd_1227*) as a single multigene operon.

Fig. S3. The protein amount of PstS2 does not change in a Δ*phoU2* mutant. Western blot for encapsulated parent strain (IU1781, lane 2), which lacks an HA epitope tag, a strain expressing *pstS2-HA* from its normal chromosomal locus (IU8421, lane 4), and *pstS2-HA* Δ*phoU2* (IU8423, lane 6). Proteins were prepared as described in *Materials and Methods*. 20 μg of protein were loaded in each lane for SDS-PAGE. Western blotting was done using anti-HA antibody as primary antibody as described in *Materials and Methods*. Quantitation showed the bands in the two strains to be the same within experimental error.

Fig. S4. Heat control for Phos-tag experiment in Fig. 3. (A) The Western blot from Fig. 3A is shown again along with the luminescence scale used to quantitate band intensities of PnpR-L-F^3~P and PnpR-L-F^3 in unheated samples from the $phoU^+$ (IU6689) and Δ*phoU2* (IU 6686) strains. (B) For this Western blot, the samples were heated before SDS-PAGE. In the heated control, the PnpR-L-F^3~P band has disappeared, consistent with the instability of the Asp~P bond in PnpR-L-F^3~P (Wayne et al., 2012). See *Materials and Methods* and the text for additional details.

Fig. S5. In unencapsulated strains, deletion mutants lacking the *pnpRS*, *pst1-phoU1*, and *pst2-phoU2* operons grow comparable to wild-type parent strain in BHI broth, which contains a high P_i concentration (≈ 18 mM). Representative growth curves are shown of the unencapsulated Δ*cps* parent strain (IU1945), Δ*cps* Δ*pnpRS* Δ*pst2-phoU2* (IU5772), Δ*cps* Δ*pst1-phoU1* Δ*pst2-phoU2* (IU5774), and Δ*cps* Δ*pnpRS-pst1-phoU1* Δ*pst2-phoU2* (IU5776) grown in BHI broth as described in *Material and Methods*. Growth experiments were repeated independently at least 3X for each strain with similar results.

Fig. S6. Parent and mutant strains grow in the C+Y broth without P_i addition. Representative growth curves of the encapsulated parent strain (IU1781) and Δ*phoU2* (IU6375), Δ*pst2* (IU6610), and Δ*pst1* (IU6638) mutants in C+Y broth containing P_i added to 48.8 mM and lacking added P_i. Cells were cultured in 3 mL C+Y with added 48.8 mM P_i overnight. The next day, cultures at OD_{620} = 0.1-0.3 were collected and washed twice with C+Y (no added P_i). Cell pellets were resuspended in 3 mL C+Y (no added P_i) and diluted in 5 mL C+Y media containing 48.4 mM P_i or lacking added P_i to OD_{620} ≈0.002. Growth experiments were repeated independently at least 3X for each strain with similar results. Growth curves were recorded as described in *Materials and Methods*.

Fig. S7. C+Y broth (no added P_i) contains ≈1.5 mM P_i. A colorimetric method was used to determine the P_i concentration in C+Y broth (no added P_i) as described in *Materials and Methods*. 2.5, 5.0, 10.0, 20.0, and 40.0 μM P_i KH_2PO_4 solutions were used generate the standard curve for the colorimetric assay (black dots). C+Y medium (no added P_i) was diluted 1:100 for P_i determination (red dot). The P_i concentration in the diluted C+Y broth ≈15 µM, indicating that the concentration of P_i in C+Y broth (no added P_i) ≈1.5 mM.

Fig. S8. Low P_i conditions increase PnpR~P RR phosphorylation up to ≈80% in *phoU*$^+$ and Δ*phoU2* strains. Phos-tag SDS-PAGE analysis was performed as described in Fig. 3 and S4 and *Materials and Methods*. (A) Phosphorylated PnpR~P and unphosphorylated PnpR in a *phoU2*$^+$ strain (IU6689) or Δ*phoU2* mutant (IU6687) expressing PnpR-FLAG^3 from its normal chromosomal locus (see Fig. 1; Table S1). The two strains were grown as indicated in mCDM containing 36.4mM P_i and shifted to mCDM containing 36.4 mM P_i or lacking added P_i for 40 min as described in *Materials and Methods*. The relative PnpR~P amount increased significantly in the Δ*phoU2* mutant to ≈33% in the high P_i medium, as expected from Fig. 3 and S4. Starvation for P_i increased the relative amount of PnpR~P to PnpR to ≈80% in the *phoU2*$^+$ strain and the Δ*phoU2* mutant. The parent wild-type (WT) control strain (IU1781) did not show any signal in the Western blot, because it does not express a protein fused to a FLAG-tag. (B) Heating control. The PnpR~P detected in the Δ*phoU2* mutant and the *phoU2*$^+$ and Δ*phoU2* strains starved for P_i disappeared upon heating due to instability of the Asp~P bond in PnpR~P (see Fig. S4).

REFERENCES TO SUPPLEMENTAL INFORMATION

Lanie, J.A., Ng, W.L., Kazmierczak, K.M., Andrzejewski, T.M., Davidsen, T.M., Wayne, K.J., et al. (2007). Genome sequence of Avery's virulent serotype 2 strain D39 of *Streptococcus pneumoniae* and comparison with that of unencapsulated laboratory strain R6. *J Bacteriol* 189**,** 38-51.

Ramos-Montanez, S., Kazmierczak, K.M., Hentchel, K.L., and Winkler, M.E. (2010). Instability of *ackA* (acetate kinase) mutations and their effects on acetyl phosphate and ATP amounts in *Streptococcus pneumoniae* D39. *J Bacteriol* 192**,** 6390-6400.

Ramos-Montanez, S., Tsui, H.C., Wayne, K.J., Morris, J.L., Peters, L.E., Zhang, F., et al. (2008). Polymorphism and regulation of the *spxB* (pyruvate oxidase) virulence factor gene by a CBS-HotDog domain protein (SpxR) in serotype 2 *Streptococcus pneumoniae*. *Mol Microbiol* 67**,** 729-746.

Wayne, K.J., Sham, L.T., Tsui, H.C., Gutu, A.D., Barendt, S.M., Keen, S.K., et al. (2010). Localization and cellular amounts of the WalRKJ (VicRKX) two-component regulatory system proteins in serotype 2 *Streptococcus pneumoniae*. *J Bacteriol* 192**,** 4388-4394.

Wayne, K.J., Li, S., Kazmierczak, K.M., Tsui, H.C., and Winkler, M.E. (2012). Involvement of WalK (VicK) phosphatase activity in setting WalR (VicR) response regulator phosphorylation level and limiting cross-talk in *Streptococcus pneumoniae* D39 cells. *Mol Microbiol* 86**,** 645-660.

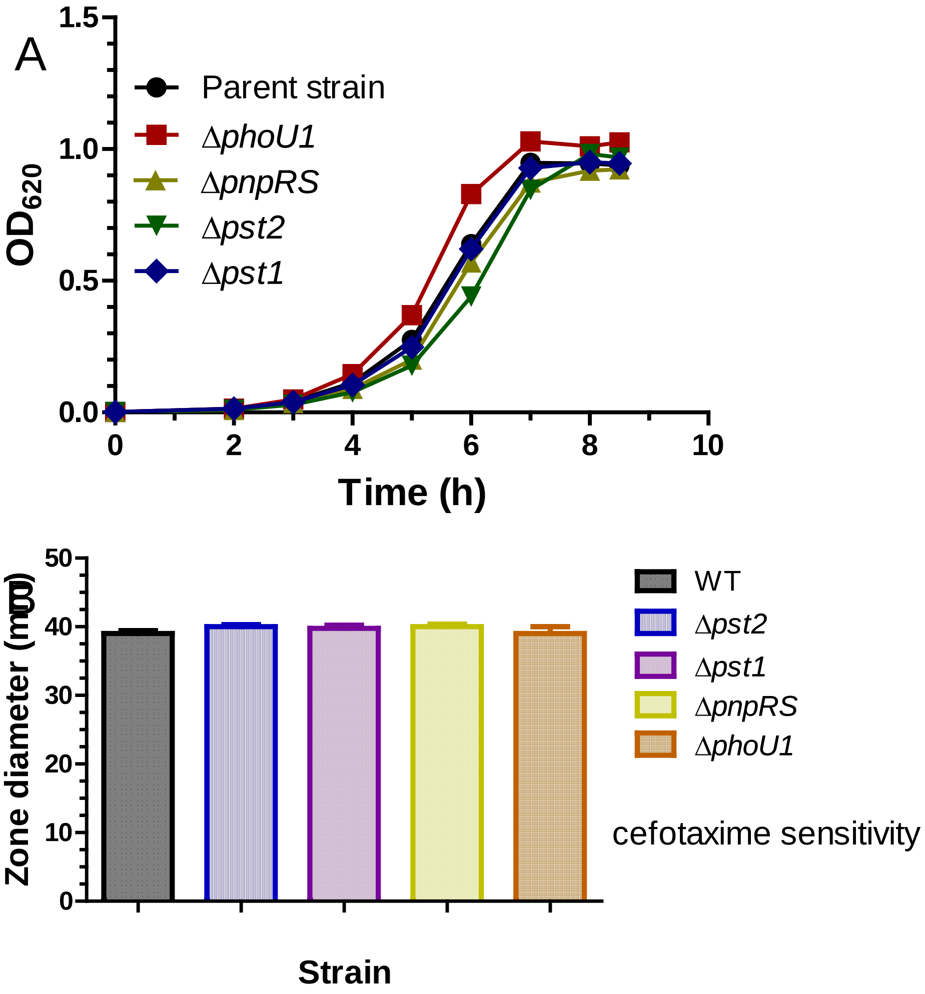
A
1.5
1.0
0.5
0.0
OD620
0
2
4
6
8
10
Time (h)
Parent strain
ΔphoU1
ΔpnpRS
Δpst2
Δpst1
50
40
30
20
10
0
Zone diameter (mm)
Strain
WT
Δpst2
Δpst1
ΔpnpRS
ΔphoU1
cefotaxime sensitivity

Fig. S1

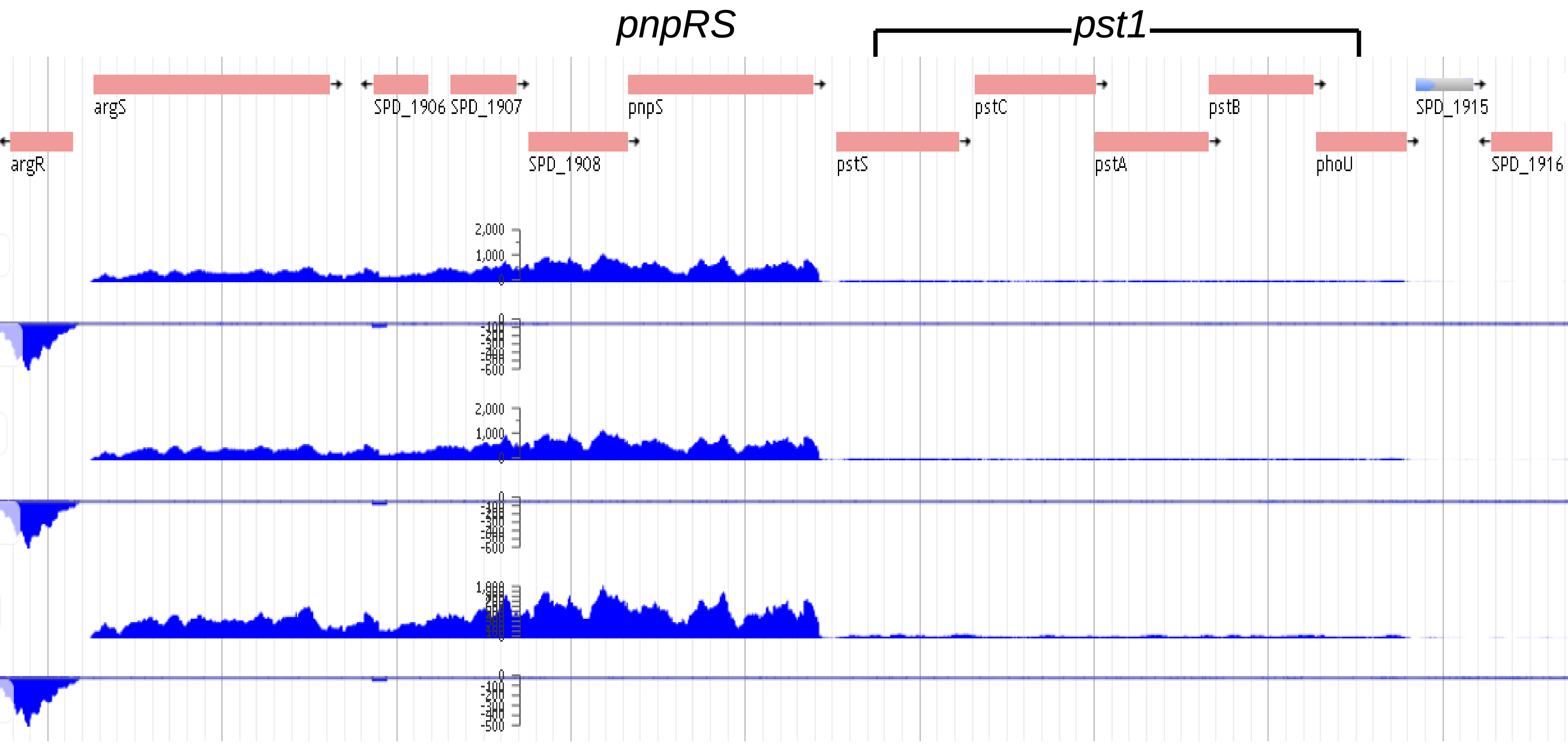
pnpRS
pst1
argS
SPD_1906
SPD_1907
pnpS
pstC
pstB
SPD_1915
argR
SPD_1908
pstS
pstA
phoU
SPD_1916
2,000
1,000
0
-600
-500

Fig. S2A

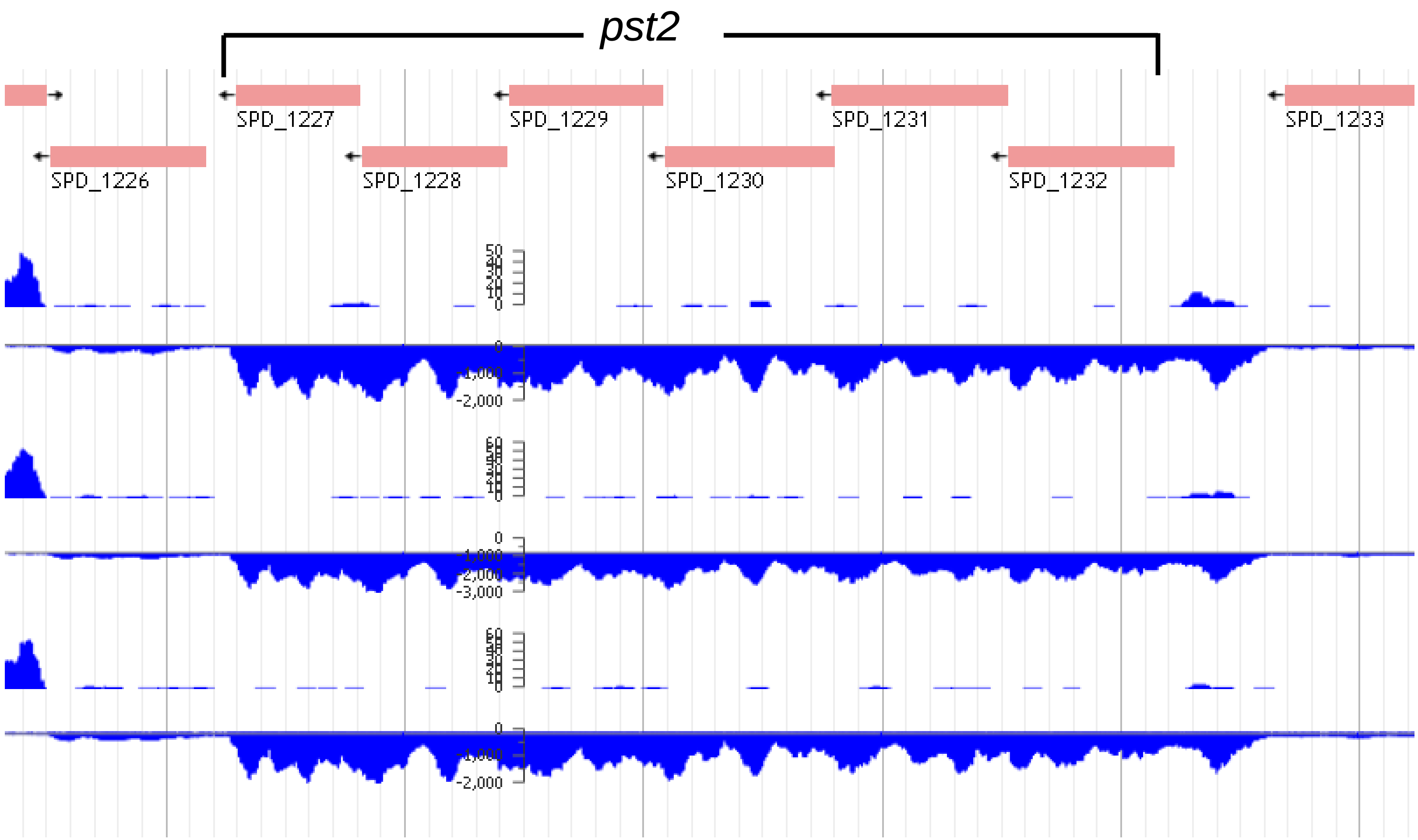

pst2
SPD_1226
SPD_1227
SPD_1228
SPD_1229
SPD_1230
SPD_1231
SPD_1232
SPD_1233

Fig. S2B

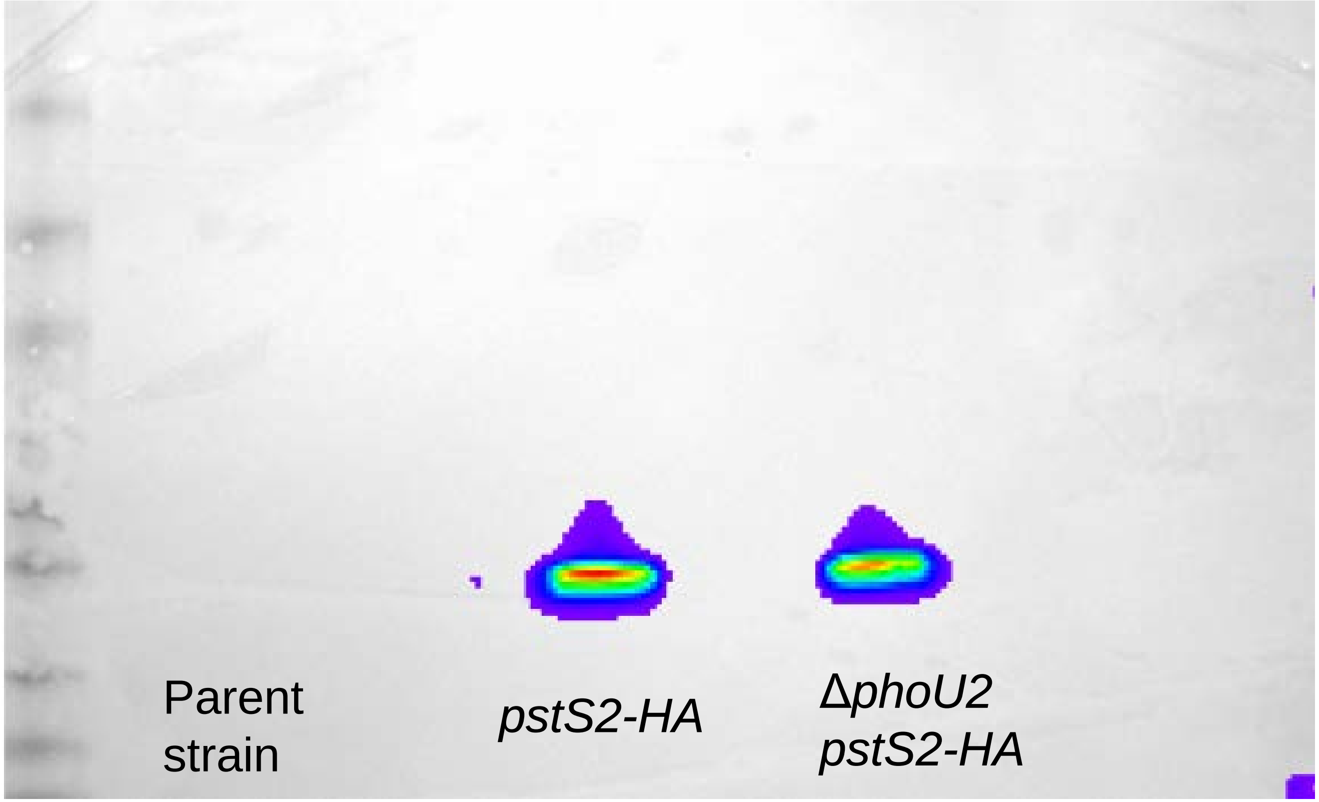

Fig. S3

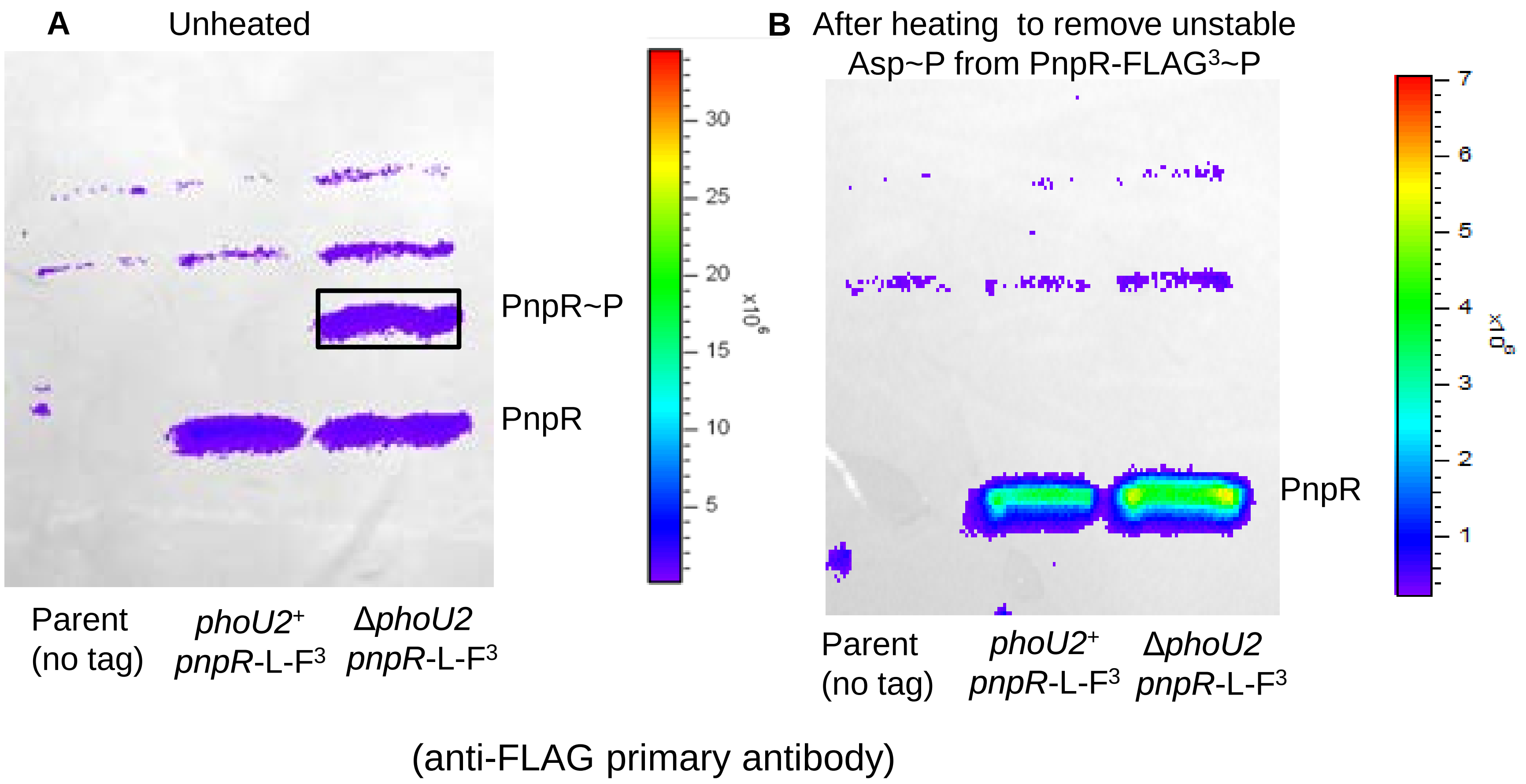

A
Unheated
PnpR~P
PnpR
30
25
20
15
10
5
x10^6
Parent
(no tag)
phoU2+
pnpR-L-F3
ΔphoU2
pnpR-L-F3
B
After heating to remove unstable
Asp~P from PnpR-FLAG3~P
PnpR
7
6
5
4
3
2
1
x10^6
Parent
(no tag)
phoU2+
pnpR-L-F3
ΔphoU2
pnpR-L-F3
(anti-FLAG primary antibody)

Fig. S4

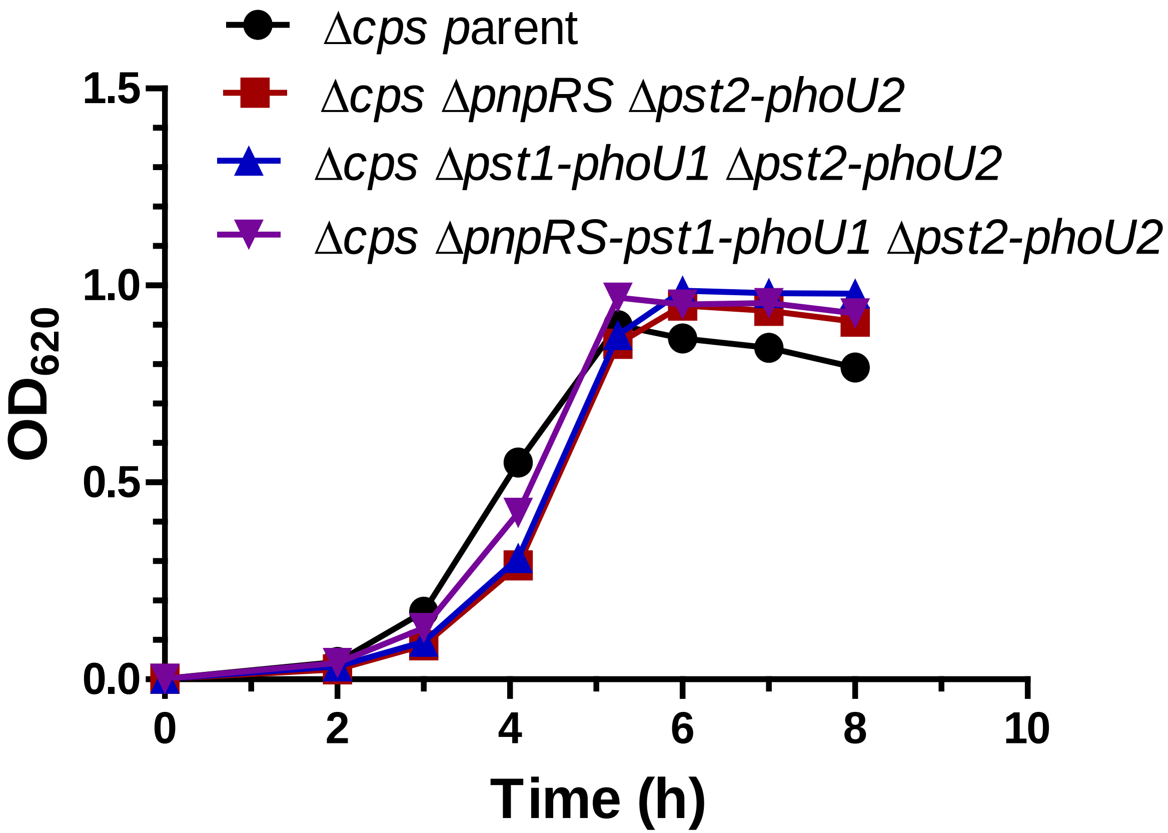
Δcps parent
Δcps ΔpnpRS Δpst2-phoU2
Δcps Δpst1-phoU1 Δpst2-phoU2
Δcps ΔpnpRS-pst1-phoU1 Δpst2-phoU2
1.5
1.0
0.5
0.0
OD620
0
2
4
6
8
10
Time (h)

Fig. S5

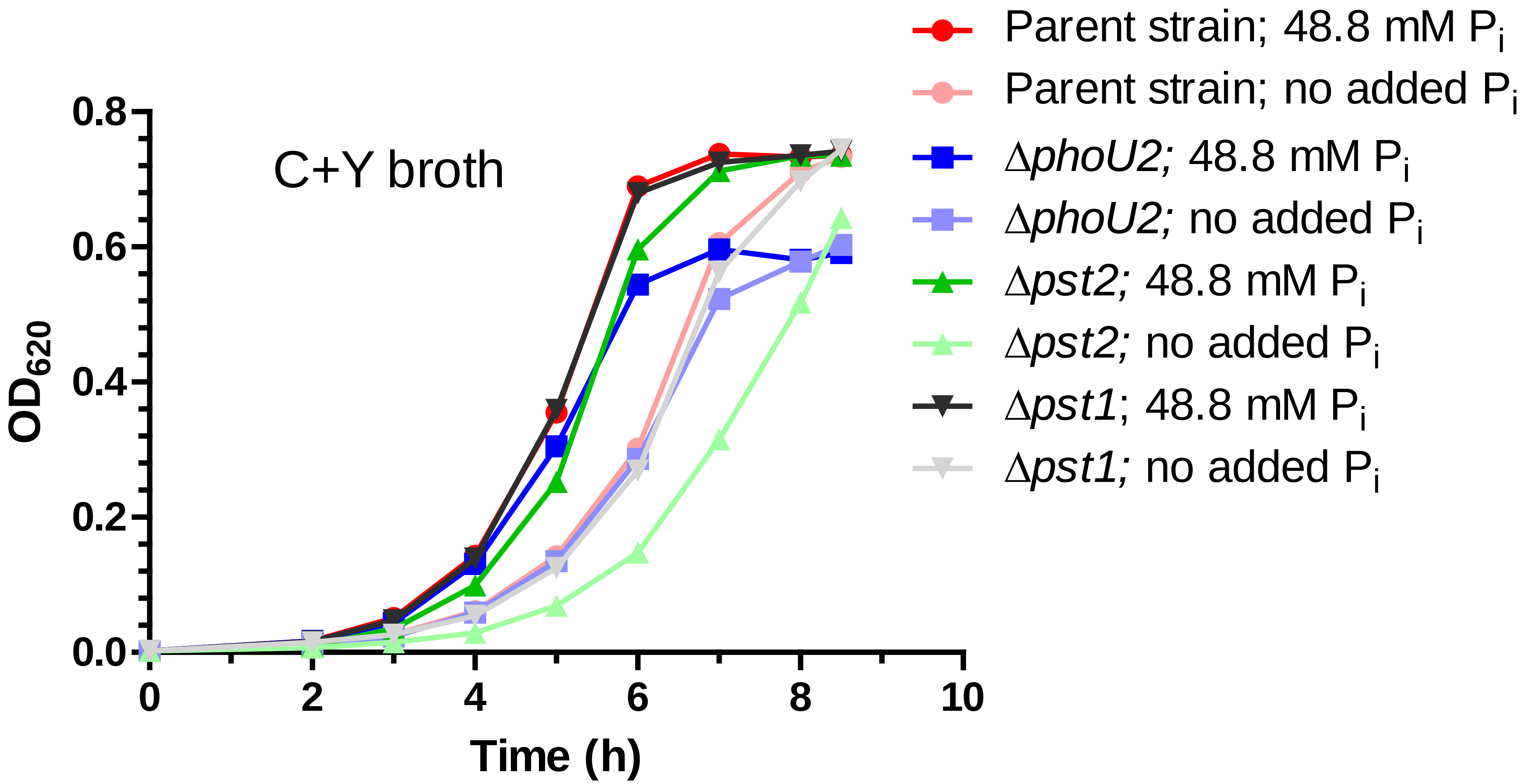
C+Y broth
OD620
0.0
0.2
0.4
0.6
0.8
0
2
4
6
8
10
Time (h)
Parent strain; 48.8 mM Pi
Parent strain; no added Pi
ΔphoU2; 48.8 mM Pi
ΔphoU2; no added Pi
Δpst2; 48.8 mM Pi
Δpst2; no added Pi
Δpst1; 48.8 mM Pi
Δpst1; no added Pi

Fig. S6

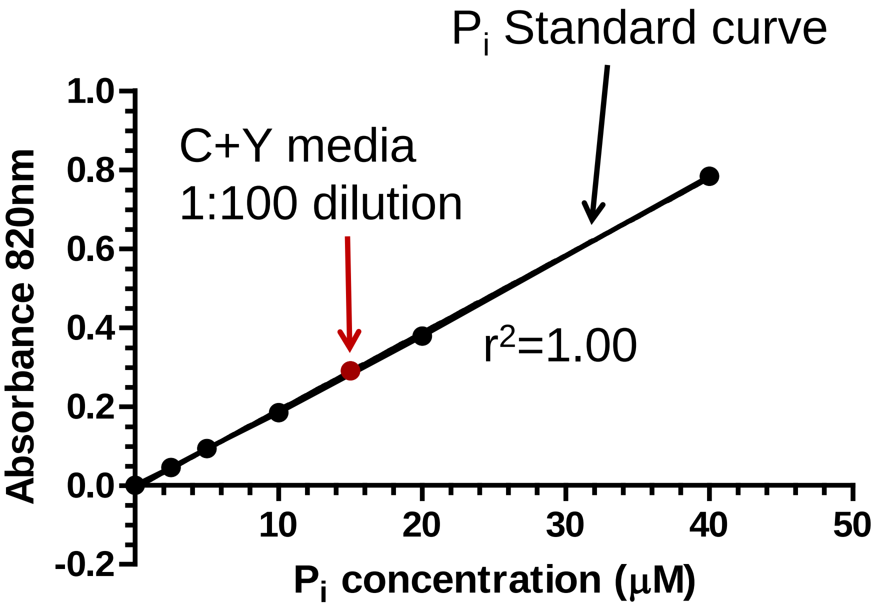
Pi Standard curve
C+Y media
1:100 dilution
r2=1.00
Absorbance 820nm
1.0
0.8
0.6
0.4
0.2
0.0
-0.2
10
20
30
40
50
Pi concentration (μM)

Fig. S7

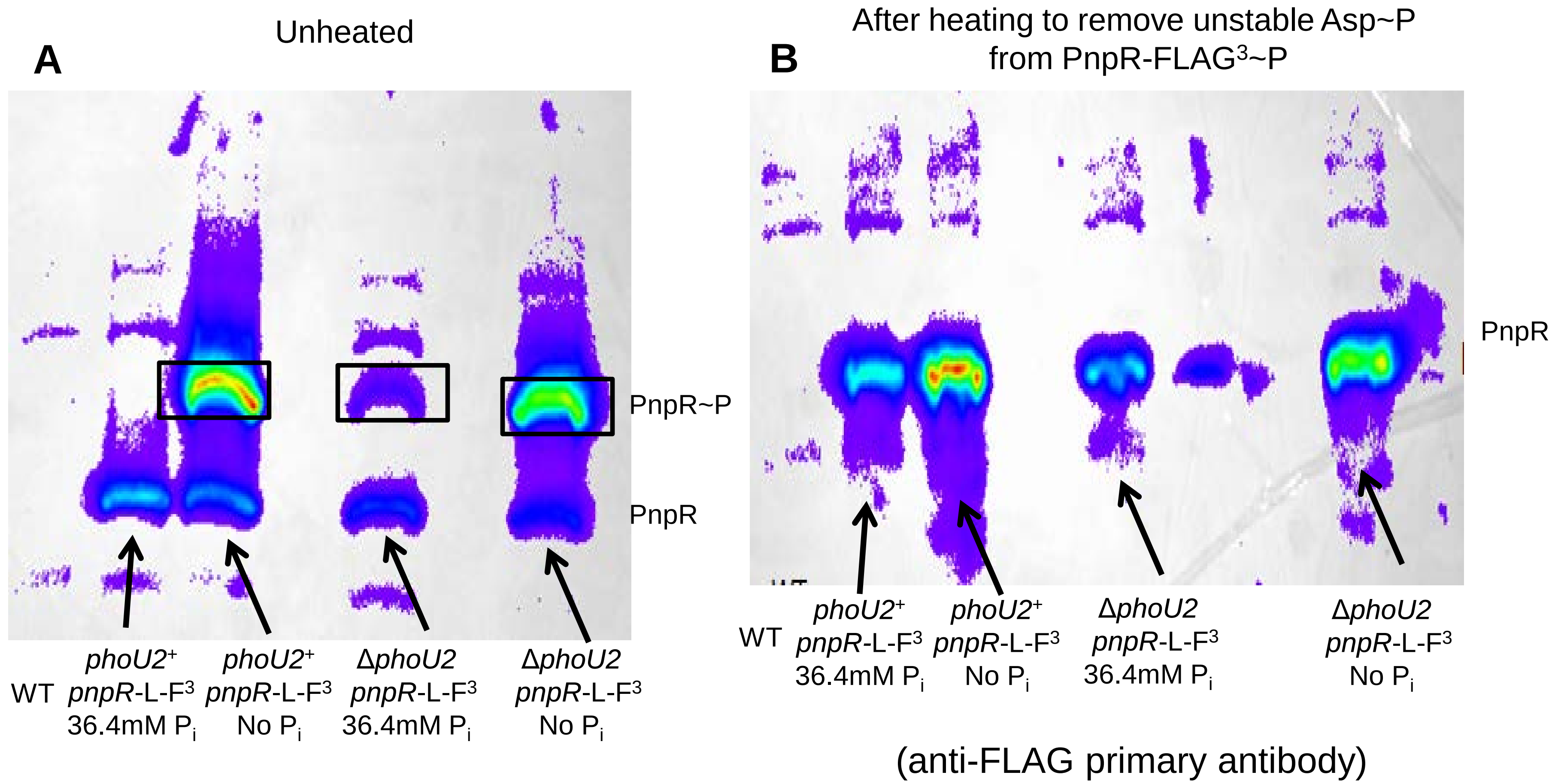

Percentage of PnpR~P amount (n=1)

phoU2$^+$ 36.4mM P_i: 0.4%

phoU2$^+$ No P_i: 80%

Δ*phoU2* 36.4mM P_i: 33%

Δ*phoU2* No P_i: 83%

Fig. S8
